# Advances in platinum-based cancer therapy: overcoming platinum resistance through rational combinatorial strategies

**DOI:** 10.1007/s12032-025-02812-3

**Published:** 2025-06-16

**Authors:** Nur Aininie Yusoh, Haslina Ahmad, Katherine A. Vallis, Martin R. Gill

**Affiliations:** 1 Department of Radiology, Huaxi MR Research Center (HMRRC), Institution of Radiology and Medical Imaging, West China Hospital of Sichuan University, Sichuan University, Chengdu, Sichuan China; 2 Department of Chemistry, Faculty of Science, Universiti Putra Malaysia, 43400 UPM Serdang, Selangor Malaysia; 3 UPM-MAKNA Cancer Research Laboratory, Institute of Bioscience, Universiti Putra Malaysia, 43400 UPM Serdang, Selangor Malaysia; 4 Department of Oncology, University of Oxford, Oxford, UK; 5 Department of Chemistry, Faculty of Science and Engineering, Swansea University, Swansea, UK

**Keywords:** Platinum, Platinum resistance, Cancer, Combination therapy, Drug synergy

## Abstract

Platinum-based cancer therapy remains a cornerstone of first-line treatment for several solid tumours such as ovarian, testicular, and non-small cell lung cancers, where it has received regulatory approval as both monotherapy and combination regimens. However, the inevitable emergence of resistance has necessitated extensive preclinical and clinical efforts to develop rational platinum-based combinations. The most appealing candidates for combination therapy are those that offer additive and/or synergistic effects without undesirable overlapping toxicities. Whilst early strategies focussed on co-administration with cytotoxic chemotherapies, recent advances have shifted towards combinations with targeted therapies and immunotherapies, offering improved efficacy and durability of response. In this review, we provide a comprehensive analysis of recent clinical trials evaluating platinum-based combination strategies (excluding radiotherapy) and give an overview of trial concepts that will lead to more refined therapies for cancer. We also highlight emerging dual-drug codelivery nanosystems, platinum-based antibody–drug conjugates (ADCs), and multi-targeted platinum compounds with promising preclinical and/or clinical evidence. Beyond traditional drug pairings, the improved design strategies of new platinum compounds such as their incorporation into ADCs offer enhanced targeting and reactivity. Whilst promising preclinical examples like trastuzumab-Pt(II) and cetuximab-C8Pt(IV) bring optimism to combinatorial approaches, significant challenges including stability and controlled payload release remain to be addressed before clinical translation. By integrating advances in molecular profiling and rational drug development, platinum-based therapies continue to evolve, offering renewed optimism for overcoming drug resistance and improving patient outcomes, although challenges such as biomarker identification, toxicity management, and treatment costs remain to be fully addressed.

**Graphical abstract:**

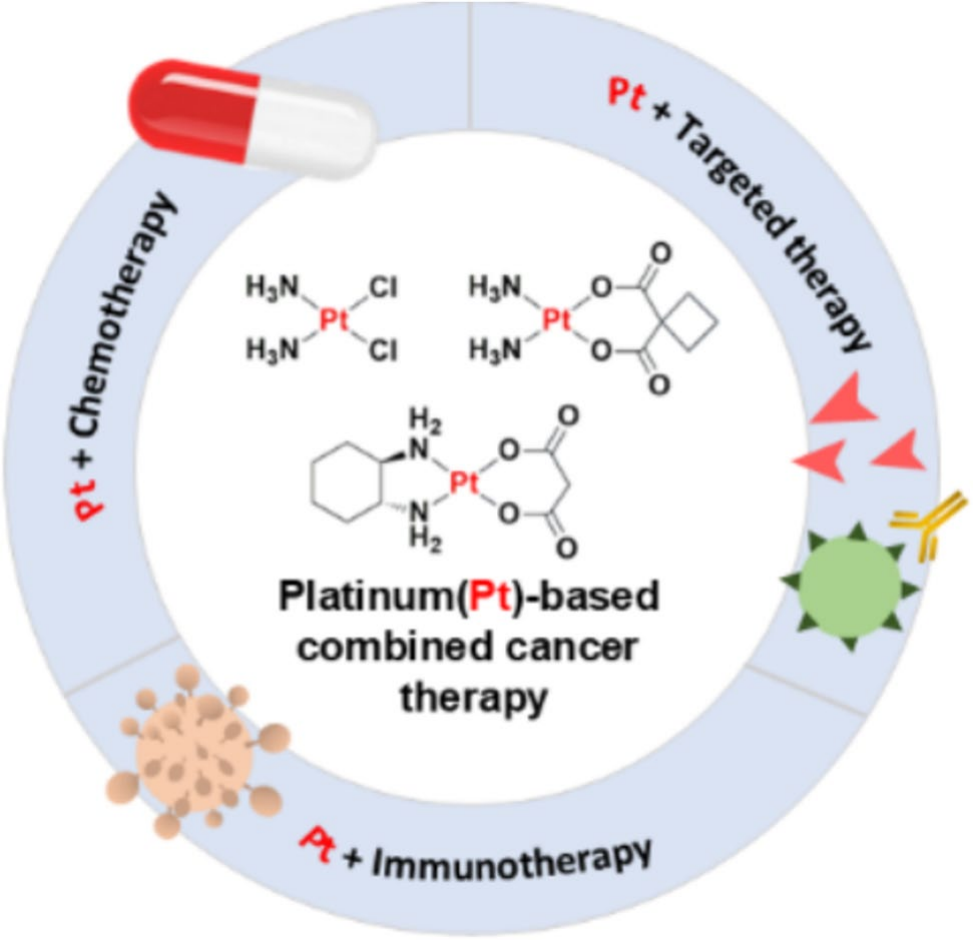

**Supplementary Information:**

The online version contains supplementary material available at 10.1007/s12032-025-02812-3.

## Introduction

**Fig. 1:**
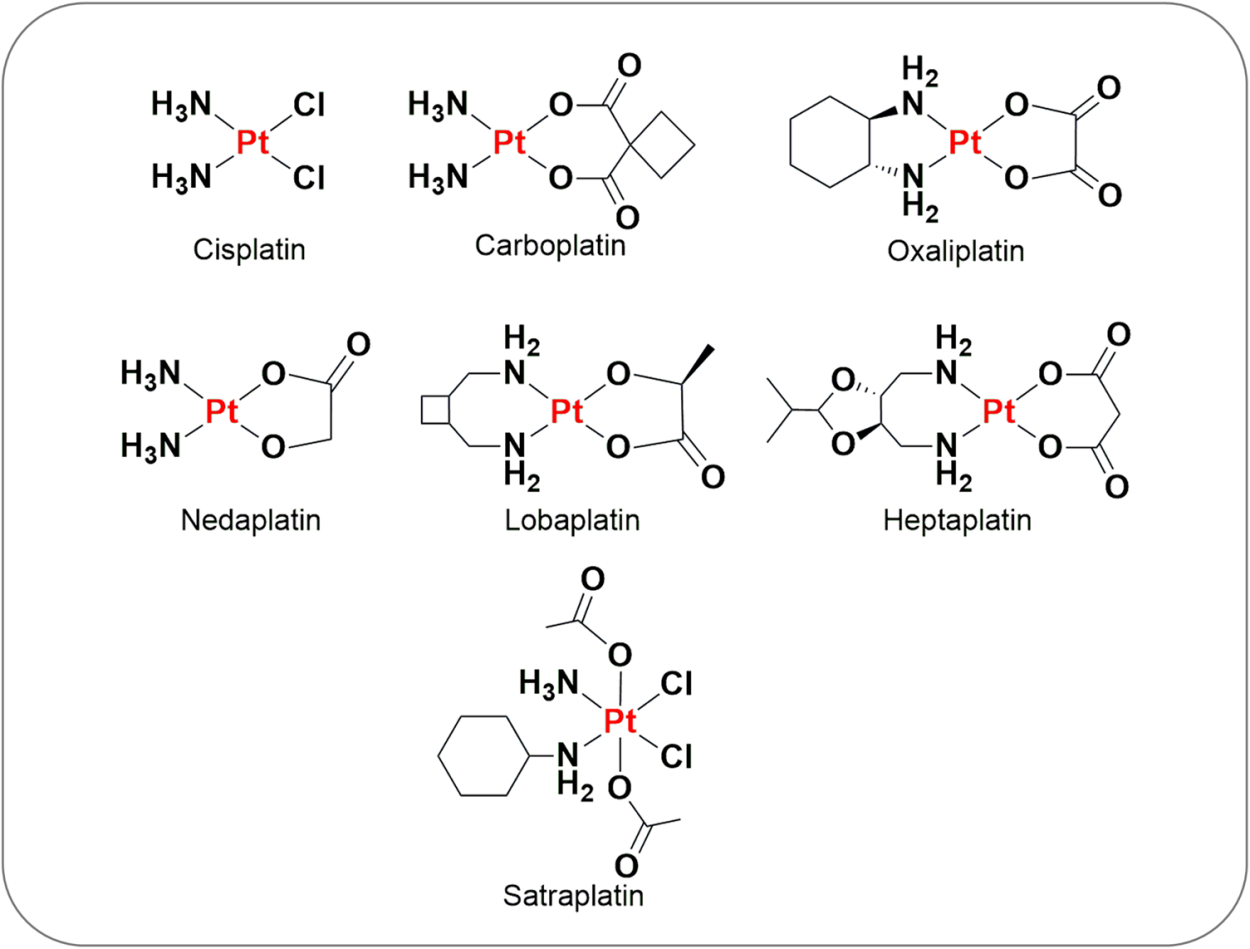
Clinical and FDA-approved anti-cancer platinum-based metallodrugs

Cancer remains a burden on society due to its high mortality and morbidity, as well as the economic strain it places on healthcare systems [1, 2]. Amongst the established chemotherapeutic agents, metallodrugs have been extensively used for cancer therapy where they offer versatile electronic and unique structural features [3–5]. The tuning of the metal, ligand, or metal–ligand interaction provides exceptional structural diversity and novel chemistry for drug design, resulting in a greater range of functions and unique mechanisms of action. In contrast to organic drugs, metallodrugs are often 'prodrugs' that convert into active forms once they enter the body or when they reach the desired target. This activation frequently involves the displacement or dissociation of one or more labile ligands, the opening of chelate rings, or a change in the oxidation state of the metal and/or ligands. Additionally, metallodrugs can also be selectively activated by external stimuli such as light, radiation, ultrasound, or heat upon reaching the target site. The antiproliferative effects of cisplatin (cis-diamminedichloroplatinum(II)) (Fig. 1) were discovered in 1965 by Barnett Rosenberg with the aid of serendipity, where his group observed that the inhibition of bacterial growth was not caused by electric fields, but rather by the platinum (Pt) compound that was released from the electrodes [6]. In 1978, the FDA granted regulatory approval for cisplatin to be used for the treatment of a variety of solid malignancies, making it one of the most successful therapeutic metallodrugs to date [6, 7]. Cisplatin exerts its cytotoxic effects through a complex interplay of molecular mechanisms, primarily by coordinating DNA to form platinum–DNA adducts, resulting in DNA damage, mitochondrial dysfunction, reactive oxygen species (ROS) generation, modulation of cell death pathways and subsequent apoptosis [7]. These multifaceted effects underscore the potency of cisplatin as a chemotherapy agent. However, despite being effective in numerous cases, cisplatin is linked to significant off-target toxicity, including nephrotoxicity, ototoxicity, and neurotoxicity [8]. Also, constant or prolonged cisplatin treatment often results in the acquisition of resistance through the selection bias of alternative cell survival pathways, leading to therapeutic failure and poor prognosis [9, 10].

Given the early success of cisplatin and to overcome its drawbacks, second- and third-generation cisplatin analogues were developed. One of the most successful platinum analogues is carboplatin (cis-diammine(1,1-cyclobutanedicarboxylato) platinum(II)) (Fig. 1) which was reported by Cleare and Hoeschele in 1973, and then gained FDA approval in 1989 [11]. Shortly after this, oxaliplatin (1R,2R-diaminocyclohexane oxalatoplatinum(II)) (Fig. 1) received European approval in 1999 and FDA approval in 2002 [12]. These second-generation platinum-based metallodrugs were developed with specific goals such as reducing toxicity, improving stability, and broadening the range of activity compared to cisplatin. Other drugs of a similar design were subsequently developed including nedaplatin, lobaplatin, and heptaplatin (Fig. 1), which have each gained approval in Japan, China, and South Korea, respectively [13]. Other than these drugs, satraplatin (Fig. 1) was developed, exciting great interest due to its high oral availability such that it can be administered in pill form, greatly improving patient convenience and reducing health care costs [14, 15]. Satraplatin has proven that overcoming the initially limiting conditions for platinum drug design (platinum(II) and cis-conformation) will help fuel the design of new lead compounds with improved functionality.

**Fig. 2:**
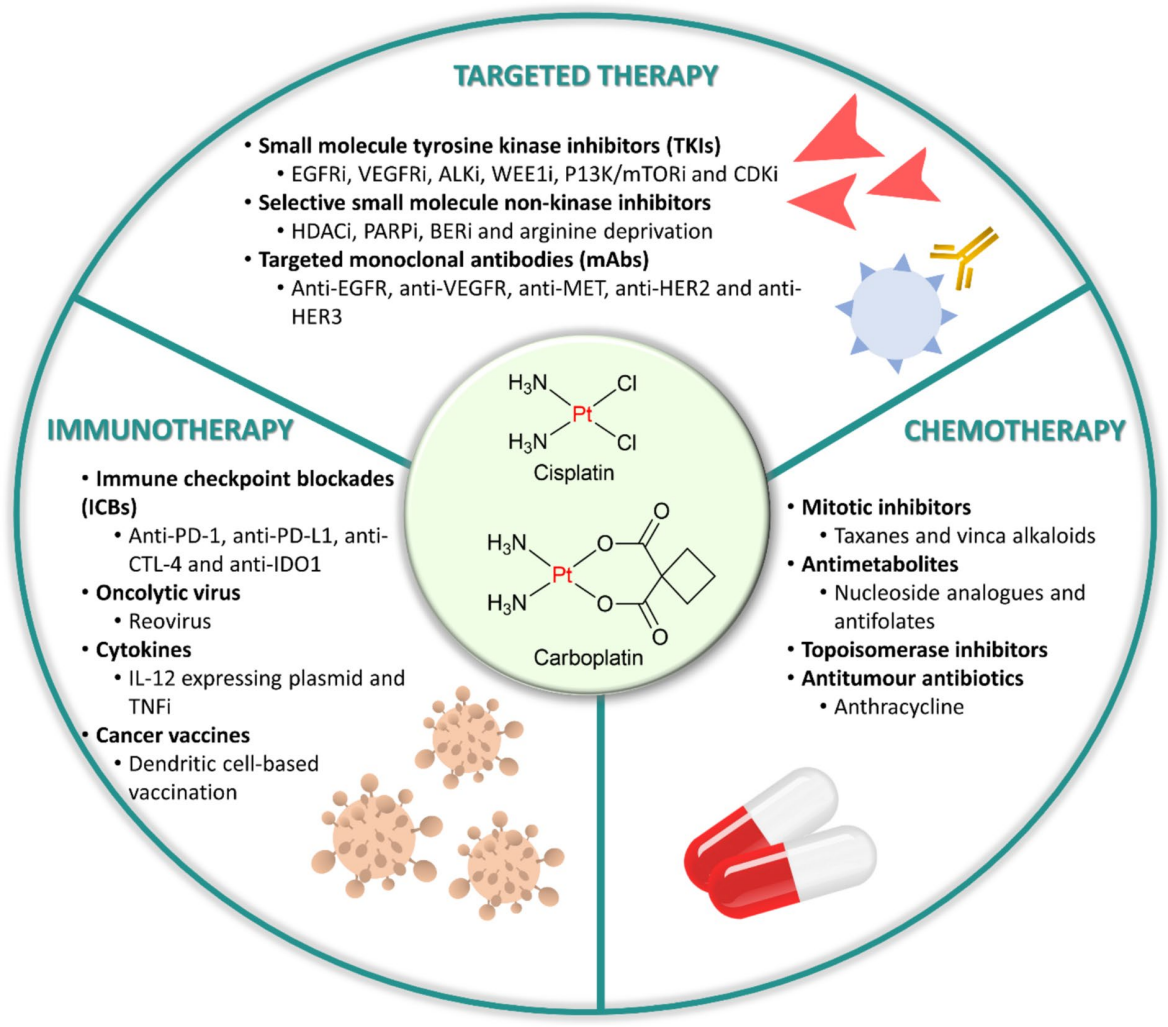
Combination strategies of platinum-based drugs (cisplatin or carboplatin) with various therapies have been explored extensively, with cytotoxic agents accounting for 37% of combinations, targeted therapies representing 44%, and immunotherapies comprising 18%. These approaches aim to enhance the therapeutic efficacy of platinum-based chemotherapy by leveraging different mechanisms of action. Cytotoxic agents, such as taxanes and antimetabolites, amplify DNA damage and impair tumour cell repair processes. Targeted therapies, including tyrosine kinase inhibitors and monoclonal antibodies (mAbs), selectively enhance the vulnerability of cancer cells to platinum drugs. Immunotherapies, particularly immune checkpoint inhibitors targeting PD-1/PD-L1 and CTLA-4, further potentiate the anti-cancer response by modulating the tumour microenvironment

Anti-cancer monotherapies, whether broadly active cytotoxics or even the most effective molecularly targeted drugs, have limited ability to induce long-lasting clinical responses, as drug resistance is common [16]. Moreover, single-drug therapy is typically inadequate for patients with advanced disease, leading to rapid disease progression and poor clinical outcomes [17]. As a result, combination therapies of two or more drugs now dominate modern cancer medicine. Specifically, additive and/or synergistic relationships can convert less effective single-drug treatments into regimens with robust anti-tumour activity [17]. Drug synergy is achieved when the combined effect of two or more drugs is greater than that predicted by their individual potencies where this has the advantages of: (1) increasing drug efficacy; (2) significantly lower therapeutic doses for each individual drug compared to when they are administered alone; (3) minimising the development of drug resistance or relapse; (4) achieving high cancer selectivity (cancer cell killing without affecting normal cells); and (5) expanding the range of treatment-responsive cancers [18, 19]. Many reviews have provided comprehensive perspectives on the use of platinum metallodrugs given as monotherapies [20, 21]. In this review, we specifically focus on the use of these platinum metallodrugs in combinatorial therapy to treat various cancers, considering the potential benefits compared to the risks in such approaches. We present examples of investigations into such combinations, with a significant emphasis on recent clinical studies, the majority of which have been completed since 2019. The primary endpoints of these clinical trials were highlighted, and the available clinical data is reported in Supplementary Table S1. Current and completed trials have examined platinum drugs with various therapies, including cytotoxic agents (37%), targeted therapies (44%), and immunotherapies (18%) (Fig. 2).

## Platinum/cytotoxic combinations

### Established platinum/cytotoxic combinations

Given the mounting clinical evidence, platinum-based doublet chemotherapy regimens have gained FDA approval and become the standard first-line treatment for specific cancers, including ovarian and non-small cell lung cancers (NSCLC), reflecting their demonstrated clinical efficacy. However, their use is not universal across all cancer types, such as prostate or breast cancer, where other therapeutic strategies are preferred. Notably, paclitaxel (taxane), in combination with either cisplatin or carboplatin, was approved by FDA for treating patients with advanced ovarian cancer after initial surgery [22], and NSCLC [23, 24]. Due to the success of this regimen, the platinum/cytotoxic combinations have been examined in current clinical settings for various advanced cancers. In an intriguing approach, a phase I study has investigated the combination of carboplatin with paclitaxel injection concentrate for nano-dispersion (PICN) in patients with advanced solid malignancies (ClinicalTrials.gov Identifier: NCT01304303) [25]. These nanoformulations offer the advantage of a wider therapeutic window and reduced toxicity. Due to observed efficacy in part A of the study, an early efficacy assessment of this treatment was also conducted in patients with unresectable biliary tract cancers (BTCs). Notably, this study demonstrated that PICN either alone or in combination with carboplatin was safe and had stable pharmacokinetics, thereby warranting further phase II trials. Pegylated liposomal doxorubicin (PLD) is doxorubicin encapsulated within a sterically stabilised liposome. Delivery using this pegylated liposomal carrier increases the circulating half-life of doxorubicin from approximately 3–55 h, whilst reducing cardiac toxicity and myelosuppression compared to conventional doxorubicin [26]. Consequently, the combinations of doxorubicin or PLD with platinum drugs have been explored in phase II/III clinical studies (NCT02413320 and NCT00538603) [27, 28]. Results from the phase III trial, which evaluated the carboplatin/PLD combination in patients with partially platinum-sensitive ovarian cancer, demonstrated a prolonged median progression-free survival (mPFS) compared to those treated with carboplatin/paclitaxel (11.3 vs. 9.4 months; *P* = 0.005, stratified log-rank test) [28]. Notably, the carboplatin/PLD regimen also showed more favourable risk–benefit profile than the standard carboplatin/paclitaxel suggesting its potential as an alternative to standard therapy for ovarian cancer patients. Recently, acelarin, a phosphoramidate transformation of gemcitabine, was developed to improve upon the limitations of gemcitabine (a nucleoside analogue), including stability, uptake, and resistance issues, potentially offering enhanced efficacy and an improved therapeutic profile. To date, acelarin remains an investigational agent and is not currently FDA-approved or in routine clinical use. Interestingly, it was evaluated in combination with cisplatin in a phase Ib clinical trial in patients with locally advanced or metastatic BTC (NCT02351765) [29]. This combination demonstrated an objective response rate (ORR) of 33%, a mPFS of 7.2 months and a median overall survival (mOS) of 9.6 months, comparable to outcomes achieved with standard cisplatin/gemcitabine treatment.

Subsequent studies have investigated the potential of a three-drug combination, for instance, cisplatin/gemcitabine/S-1 (an oral fluoropyrimidine) in patients with advanced BTC (NCT02182778) [30]. This study reported modest but statistically significant improvements in mOS (13.5 vs. 12.6 months; *P* = *0.046*, stratified log-rank test), mPFS (7.4 vs. 5.5 months; *P* = *0.015*), and response rate (RR, 41.5% vs. 15%) for the cisplatin/gemcitabine/S-1 (CGS) regimen compared to cisplatin/gemcitabine (CG). Although the absolute gains were limited, the findings suggest a potential clinical benefit of the CGS regimen, warranting further investigation in specific patient subgroups. A four-drug combination of cisplatin/docetaxel/gemcitabine/capecitabine was assessed in patients with metastatic pancreatic cancer (NCT01459614), where this regimen demonstrated survival benefit and was safe and well tolerated [31]. For this reason, a subsequent study was conducted to examine the addition of irinotecan to the cisplatin/gemcitabine/docetaxel/capecitabine regimen in patients with metastatic pancreatic cancer (NCT02324543) [31, 32]. Although the study did not achieve its primary endpoints, the treatment regimen was generally safe and well tolerated, indicating that additional refinement may be beneficial. The study reported that the most common grade 3 or higher treatment-related adverse events were anaemia (60%), neutropenia (60%), and leukopenia (47%), with no treatment-related deaths reported. Although these combinations using multiple drugs simultaneously may not directly target platinum resistance mechanisms, they have the potential to increase efficacy and delay the development of resistance when compared to platinum monotherapy. For example, a study by Shroff et al. demonstrated that the combination of cisplatin with nab-paclitaxel/gemcitabine resulted in improved clinical outcomes, including mOS of 19.2 months and a mPFS of 11.8 months in patients with advanced BTC (NCT02392637) [33], indicating enhanced efficacy and potential to mitigate resistance.

### Overlapping toxicity

Despite emerging evidence of augmented anti-tumour activity from platinum/cytotoxic combinations, their use is often hindered by unacceptable overlapping toxicity. This caution arises from the recognised side effects of platinum drugs, particularly when combined with other chemotherapy agents that target rapidly dividing cells, such as taxanes. For instance, in a randomised phase III trial, carboplatin/paclitaxel combination showed clinical benefits but was associated with significant toxicities, including grade 3/4 neutropenia (50%), grade 2 alopecia (86%), neuropathy and hypersensitivity reactions [34]. Although the combination of carboplatin and amrubicin (a topoisomerase inhibitor) demonstrated clinical benefits in patients with extensive-stage small cell lung cancer (SCLC) (NCT01076504) [35], no increased efficacy compared to standard treatments was observed, and severe myelosuppression was noted. Despite the fact that certain platinum/cytotoxic combinations are associated with notable toxicities, including high-grade adverse events in some cases (e.g. carboplatin/paclitaxel-induced grade 3/4 neutropenia in approximately 50% of patients), many regimens have also demonstrated enhanced clinical benefits compared to monotherapy, with manageable or acceptable safety profiles in specific patient populations. For instance, a platinum/docetaxel combination achieved a pathologic complete response (pCR) rate of 52% with a favourable toxicity profile in triple-negative breast cancer (TNBC) patients (NCT02413320) [27]. Similarly, cisplatin combined with the vinca alkaloid vinorelbine has shown efficacy and tolerability as a first-line therapy in NSCLC patients, with an OS of 10.2 months and an acceptable safety profile (EudraCT number: 2012–003531-40) [36]. The variability in observed toxicity across studies can be attributed to differences in patient characteristics, treatment regimens, supportive care measures, and study designs [37, 38]. These findings highlight the importance of personalised treatment approaches that balance efficacy with the risk of toxicity, along with careful monitoring and supportive care to mitigate adverse effects.

## Combining platinum-based drugs with targeted therapies

**Fig. 3:**
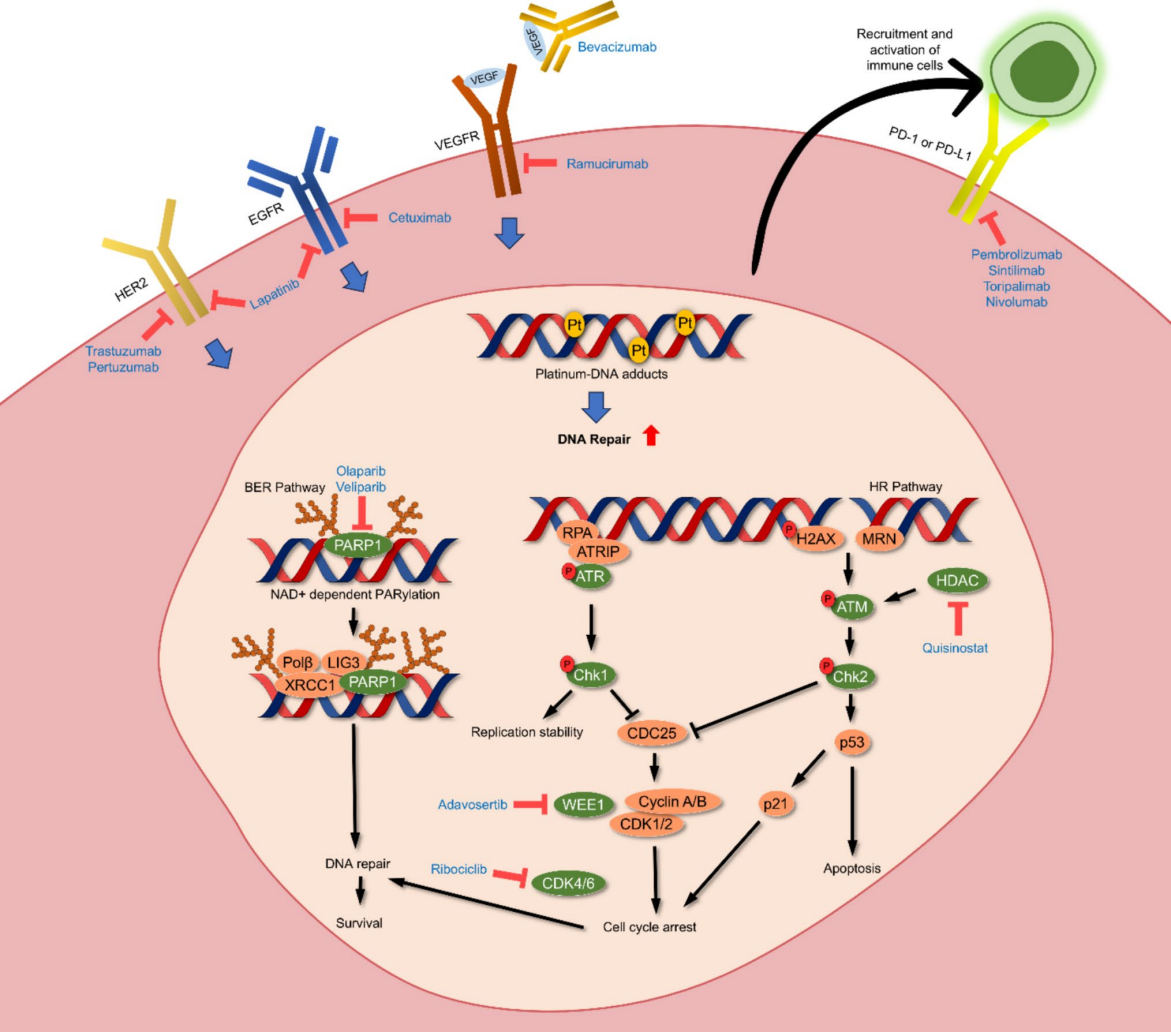
Targets for combinations with platinum-based chemotherapy. This figure illustrates key pathways that influence the response to DNA damage and are targeted in combination with platinum-based chemotherapy. These include DNA damage response (DDR) pathways such as homologous recombination (HR) and non-homologous end joining (NHEJ), which are targeted by PARP and ATM/ATR inhibitors. Additionally, cell cycle regulators such as CDK inhibitors play crucial roles in sensitising tumours to platinum therapy. Immune-related mechanisms, including checkpoint inhibitors targeting PD-1/PD-L1, enhance anti-tumour immune responses in combination with platinum drugs and overcome resistance mechanisms

Molecularly targeted small molecule drugs represent a cornerstone in cancer treatment due to their greater specificity and safety (more tumour-selective) compared to traditional chemotherapy [39]. Since the FDA approved the first tyrosine kinase inhibitor (TKI), imatinib, in 2001, numerous small molecule targeted drugs have been introduced into clinical oncology [40]. Of note, recent advancements in understanding the diverse mechanisms of platinum drugs, beyond inducing DNA damage, have facilitated the rational design of combination therapies with specific inhibitors (Fig. 3). This strategy, leveraging the complementary effects of diverse drugs, seeks to enhance anti-tumour efficacy and patient outcomes. Combination therapies that use drugs with distinct mechanisms are promising, as they can bypass resistance to both drugs.

### Tyrosine kinase inhibitors (TKIs)

Tyrosine kinases play a pivotal role in initiating intracellular signal-transduction cascades that regulate cell proliferation and survival. Given their overexpression in various cancers, inhibiting these kinases offers a targeted therapeutic approach [41]. A notable example of tyrosine kinase inhibitors (TKIs) are the WEE1 inhibitors, which regulate the G2/M transition and maintain genomic stability [42]. Inhibiting WEE1 induces premature mitosis entry, leading to mitotic catastrophe. Preclinical studies have shown that adavosertib, a potent and selective WEE1 inhibitor, enhances the efficacy of platinum chemotherapy, particularly in tumour protein p53 (TP53)-deficient tumours models [43]. This was evidenced in a phase II trial where adavosertib combined with carboplatin/paclitaxel showed clinical benefit in women with platinum-sensitive TP53-mutant ovarian cancer (NCT01357161). The combination resulted in improved mPFS compared to placebo [7.9 vs. 7.3 months; two-sided *P* = 0.080, exceeding the established significance threshold (*P* < 0.2)] [44], although it is important to note that TP53 mutation does not always equate to functional deficiency. Targeted therapies are developed to impede critical pathways implicated in tumour growth and metastasis. However, some tumours may evade treatment by using alternative pathways, necessitating a multi-targeted strategy to combat resistance [45, 46].

### Selective small molecule non-kinase inhibitors

Small molecule non-kinase inhibitors selectively bind to targets outside the kinome, effectively inhibiting downstream signalling pathways. This class of drugs includes poly(ADP-ribose) polymerase (PARP) inhibitors that have received FDA approval [47, 48]. Prominent examples of PARP inhibitors (PARPi) include olaparib, niraparib, rucaparib, talazoparib, and veliparib. Mechanistic studies indicate that platinum/PARPi synergy is associated with sustained DNA double-strand breaks (DSBs) leading to a significantly marked increase in apoptosis [49, 50]. These preclinical data have prompted clinical trials to assess this combination in various advanced cancers including TNBC, ovarian, SCLC and head and neck squamous cell carcinoma (HNSCC) (NCT01074970, NCT01063816, NCT02032277, NCT01642251, NCT01711541, NCT02163694, and NCT03150576) [51–58]. Encouragingly, in patients with extensive-stage SCLC, the addition of veliparib to a cisplatin/etoposide regimen met its primary endpoint with improved mOS (10.2 vs. 8.9 months; one-sided *P* = 0.17) and ORR (71.9% vs. 65.6%; two-sided *P* = 0.57) compared to a placebo-treated group [55]. In addition, combining veliparib with a carboplatin/paclitaxel regimen was found to be well tolerated in advanced HNSCC patients, with 2-year OS rate of 77.8% and 2-year PFS rate of 66.7% [56]. Notably, in a double-blind phase III trial, the subgroup analysis showed that the addition of veliparib to a carboplatin/paclitaxel regimen resulted in durable benefit with prolonged mPFS compared to the placebo/chemotherapy arm for all subgroups defined by homologous recombination (HR) or breast cancer susceptibility genes (BRCA1/2) status (HR + : 13.0 vs. 12.5 months, *P* = 0.013; TNBC: 16.6 vs. 14.1 months, *P* = 0.052; germline mutation in BRCA1 (gBRCA1): 14.2 vs. 12.6 months, *P* = 0.073; and gBRCA2: 14.6 vs. 12.6 months, *P* = 0.021) [57]. Importantly, the efficacy of PARPi in combination with platinum drugs also depends on the HR repair capacity of the tumours. In BRCA-deficient tumours—where HR is impaired—PARP inhibition leads to synthetic lethality, thereby improving therapeutic outcomes. In contrast, BRCA-proficient or HR-competent tumours may derive limited benefit from this strategy. For instance, the PARTNER trial reported no added benefit of olaparib when combined with neoadjuvant carboplatin/paclitaxel in BRCA-proficient TNBC, showing no improvement in pCR, event-free survival (EFS), or OS [58]. This contrasts sharply with the significant benefits observed in BRCA-deficient TNBC patients, as reported in an analysis within the PARTNER trial [59]. These findings highlight the critical role of BRCA mutation status and HRD as predictive biomarkers for PARP inhibitor efficacy and reinforce the need for biomarker-guided treatment selection. Furthermore, due to their complementary mechanisms of action, the combined use of methoxyamine, a base excision repair (BER) inhibitor, with cisplatin/pemetrexed has shown promising anti-tumour effects, notably in salivary gland tumours, whilst maintaining a tolerable safety profile at the tested doses (NCT02535312) [60].

### Targeted monoclonal antibodies (mAbs)

Monoclonal antibodies (mAbs) are engineered proteins designed to target the extracellular domains of specific antigens. Through this targeting, they disrupt ligand binding, impede subsequent activation, and block downstream signalling pathways involved in cancer cell growth and survival. For instance, the anti-epidermal growth factor receptor (EGFR) mAb cetuximab inhibits EGFR signalling, which can indirectly enhance the efficacy of DNA-damaging platinum drugs and improve their anti-tumour activity [61]. This combination is particularly effective in cancers with overexpressed or mutated EGFR. For this reason, cetuximab in combination with platinum-based regimens was evaluated in phase II/III trials (NCT01437449 and NCT02268695) [62, 63]. Subsequently, antibodies that target VEGF, also known as anti-angiogenesis agents, such as bevacizumab and ramucirumab, had undergone clinical investigations to determine their suitability as partners for platinum-based regimens (NCT00989651, NCT01160744, NCT01735071, NCT02359058, and NCT02363751) [64–67]. By inhibiting angiogenesis, these mAbs reduce the nutrient and oxygen supply to tumours and impair their ability to repair DNA damage, making them more susceptible to platinum-induced DNA damage. The addition of bevacizumab or ramucirumab to platinum-based regimens did not reveal any new or unexpected safety concerns, but neither were overall outcomes improved compared to standard platinum-based regimens, suggesting further optimisation is warranted.

Human epidermal growth factor receptor 2 (HER2) promotes cell growth and division, and is frequently overexpressed in various cancers, notably ovarian and breast carcinomas [68]. Trastuzumab and pertuzumab function by blocking HER2 signalling, thereby disrupting tumour cell proliferation. Thus, combining these anti-HER2 agents with DNA-damaging platinum drugs may enhance cancer cell death, specifically benefiting individuals with HER2-positive cancers. These combinations have been evaluated across various cancers, including urinary tract, gastric, breast, and gastroesophageal junction cancer (NCT00515411, NCT01358877, and NCT02205047) [69–71]. The addition of trastuzumab to platinum-based regimens was found to be effective and safe in patients with metastatic HER2-positive gastric cancer (ORR: 65%, mPFS: 13 months, and mOS: 24.9 months) [69]. Similarly, adding pertuzumab to trastuzumab and platinum-based chemotherapy conferred clinical benefits in patients with HER2-positive breast cancer [70].

Overall, mAbs have demonstrated encouraging clinical benefits when added to platinum regimens in various studies, albeit with some variability in results. Unlike TKIs, mAbs typically exhibit high specificity for their targets, which can result in variable outcomes influenced by factors such as tumour heterogeneity and patient selection criteria. Consequently, the development of mAbs may require careful selection of cancer types, stages, and suitable combination partners to ensure improved clinical outcomes. Nonetheless, these examples underscore how understanding the mechanisms of action of platinum drugs has facilitated the development of more effective combination therapies with targeted agents like mAbs. The key contribution of platinum-based drugs lies in their ability to induce DNA damage, which, when combined with the targeted inhibition of growth and survival pathways by mAbs, leads to enhanced anti-tumour efficacy.

### Platinum-based antibody–drug conjugates (ADCs)

**Fig. 4:**
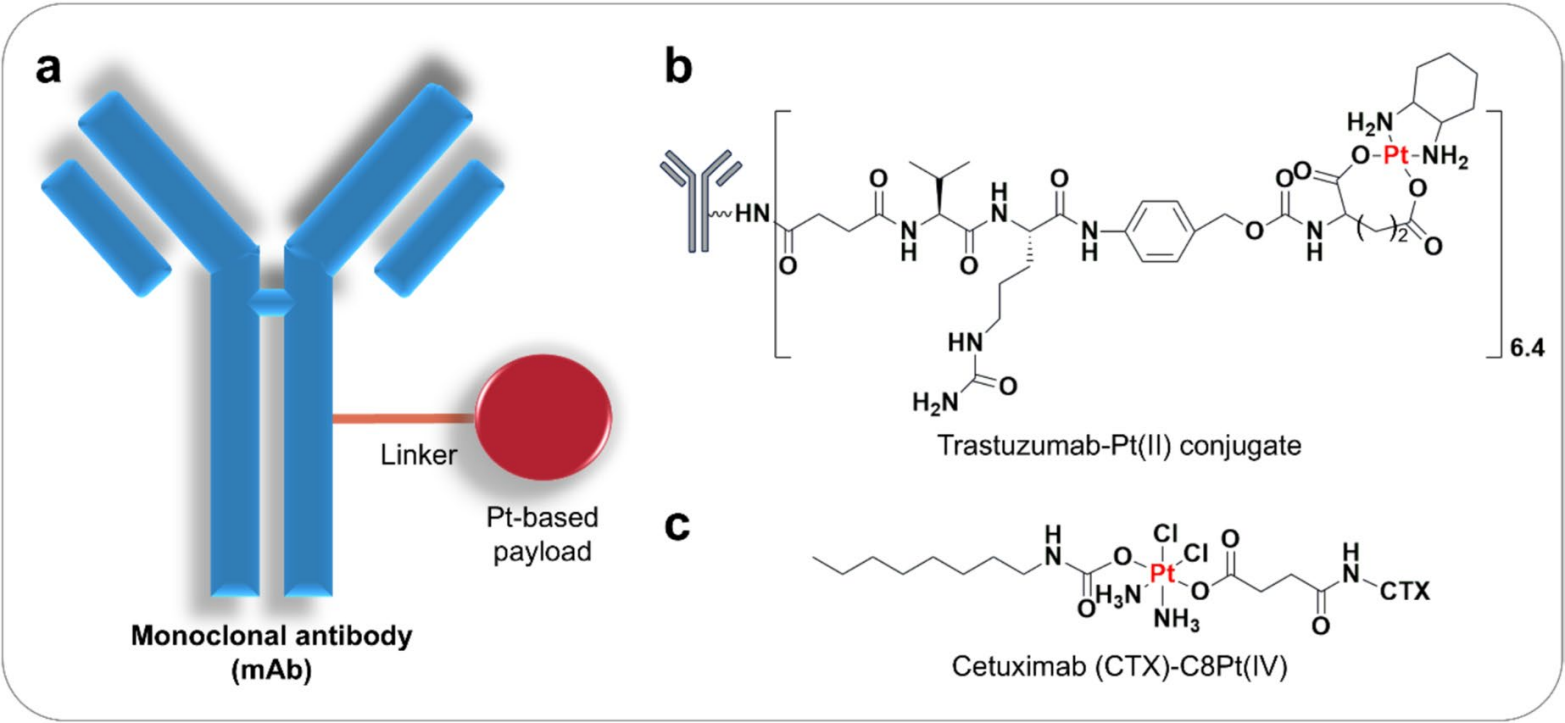
Platinum-based antibody–drug conjugate (ADC) as new strategies for specific tumour-targeting. **a** Platinum compounds are conjugated with monoclonal antibodies (mAbs) to enhance drug delivery and targeting. **b** Structure of trastuzumab-Pt(II) conjugate. **c** Structure of cetuximab-C8Pt(IV)

The advent of antibody–drug conjugate (ADC) has revolutionised cancer therapy by precisely targeting tumour antigens, improving efficacy, reducing drug toxicity, and enhancing the therapeutic window. Thanks to the advances in synthetic chemistry, ADCs have been designed to comprise tumour-targeting mAbs linked to cytotoxic payloads via intricately designed chemical linkers, simultaneously enabling potent effectiveness and precise targeting, thereby expanding the therapeutic index [72]. Mirvetuximab soravtansine is an example of an ADC where the antibody mirvetuximab (anti-folate receptor α, or anti-FRα) is linked to a cytotoxic drug called DM4 (a maytansinoid). In an intriguing approach, carboplatin was examined in combination with mirvetuximab soravtansine in patients with platinum-sensitive ovarian cancer in a phase Ib trial (NCT02606305) [73]. This combination demonstrated clinical benefit and is well tolerated (ORR: 71% and mPFS: 15 months). Whilst most ADCs in the preclinical and clinical developments rely on complex organic molecules, the potential of conjugating metallodrugs to mAbs has been largely neglected. Metallo-based ADCs might reduce the high cost associated with producing targeted chemotherapeutics, as their bioconjugation to mAbs could be simpler compared to cytotoxic payloads derived from organic molecules and natural products (Fig. 4a) [74]. Studies have shown that the conjugation of platinum drugs to trastuzumab via a cathepsin B cleavable dipeptide enhances drug accumulation and enables specific delivery to HER2-positive cancer cells (Fig. 4b) [75]. Trastuzumab-Pt(II) conjugate has been loaded with approximately 6.4 mol of platinum drugs per mole of antibody, retaining a high and selective binding affinity for the HER2 protein and HER2-positive SK-BR-3 breast cancer cells. Compared to oxaliplatin, trastuzumab-Pt(II) conjugate exhibits a higher cellular uptake of platinum drugs with improved in vitro cytotoxicity against SK-BR-3 cells. Similarly, conjugation of a new cytotoxic platinum (IV) prodrug (C8Pt(IV)) with cetuximab (Cet-C8Pt(IV)) also showed excellent tumour targeting in cutaneous squamous cell carcinoma (Fig. 4c) [76].

Compared to free platinum drugs, antibody-platinum (Ab-Pt) conjugates exhibit distinct cellular uptake mechanisms, platinum accumulation profiles, and DNA platination efficiency. Free platinum drugs such as cisplatin and oxaliplatin primarily enter cells via passive diffusion, leading to relatively non-specific intracellular distribution. In contrast, Ab-Pt conjugates are internalised via receptor-mediated endocytosis, enabling more targeted delivery to tumour cells. Experimental studies demonstrate the advantages of this approach. In HER2-positive SK-BR-3 cells, free oxaliplatin showed faster initial uptake, reaching 25 ng Pt per million cells at 4 h, approximately 1.7-fold higher than Herceptin-Pt(II) conjugate [75]. However, after 24 h, platinum accumulation in Herceptin-Pt(II)-treated cells reached 224 ng Pt per million cells, compared to only 67 ng for oxaliplatin-treated cells. This difference highlights the enhanced and sustained cellular uptake conferred by antibody targeting, attributed to the specific interaction between Herceptin and HER2 receptors and subsequent receptor-mediated endocytosis. Similarly, in EGFR-positive epidermoid carcinoma A-431 cells, treatment with a cetuximab-conjugated platinum(IV) prodrug (Cet-C8Pt(IV)) resulted in platinum concentrations 6.83 and 6.58 times higher than free C8Pt(IV) and a non-conjugated C8Pt(IV)/cetuximab mixture, respectively [76]. Competitive inhibition experiments confirmed that Cet-C8Pt(IV) targets EGFR specifically, as platinum uptake was 1.83 times higher in cells without cetuximab pretreatment. Fluorescence tracking with Cy5.5-labelled Cet-C8Pt(IV) further showed progressive cellular uptake over 6 h. Following internalisation, platinum release from Ab-Pt conjugates occur within endosomal or lysosomal compartments, typically triggered by acid-sensitive linkers or enzymatic cleavage. Successful release of the active platinum species is critical for cytosolic escape and subsequent nuclear targeting. Activated platinum species then form DNA adducts, disrupting replication and transcription. However, efficient endosomal escape remains a key factor influencing therapeutic efficacy, as entrapment in endosomes may limit access to nuclear DNA.

**Fig. 5:**
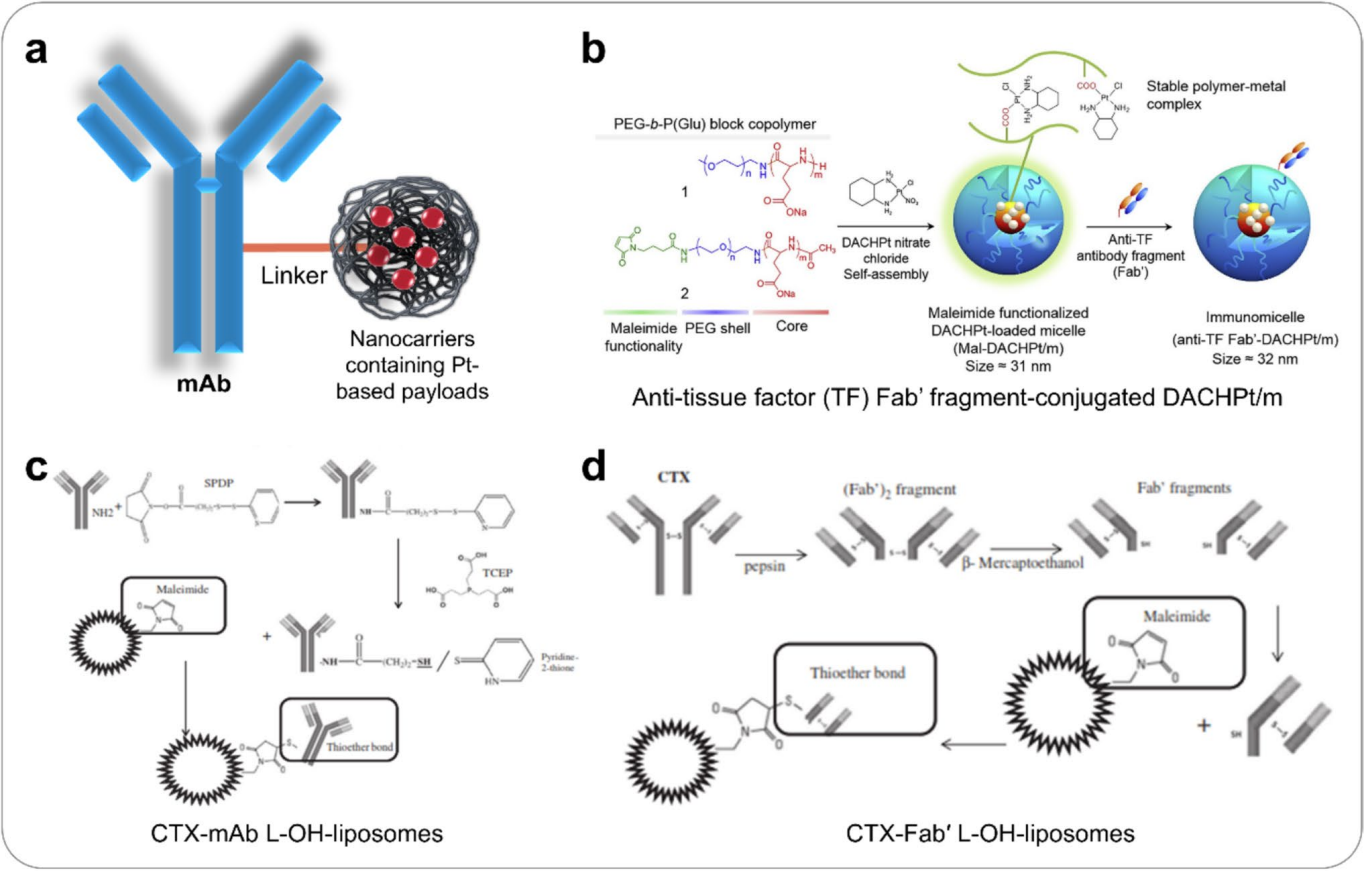
Platinum-based ADCs as new strategies for specific tumour-targeting. **a** Platinum compounds are encapsulated in carrier molecules linked to mAbs to enhance drug delivery and targeting. **b** Schematic illustration of the preparation of an anti-tissue factor (TF) antibody fragment-antigen binding (Fab') conjugated to polymeric micelles containing an active complex of oxaliplatin, (1,2diaminocyclohexane)platinum(II) (DACHPt) (DACHPt/m). Reprinted from [77], Copyright 2015, with permission from Elsevier. Schematic illustration of the method for preparing oxaliplatin (L-OH)-loaded liposomes linked to either (**c**) whole cetuximab (CTX) or (d) CTX-Fab’ fragments to their surface. Reprinted from [78], Copyright 2015, with permission from Elsevier

ADCs have demonstrated high efficacy by offering targeted delivery of cytotoxic drugs to tumours. However, they are limited by the payload they can carry. By integrating antibodies into drug-loaded nanocarriers, the ability of antibodies to deliver a wide range of therapeutic agents is significantly enhanced. Thus, platinum compounds were also encapsulated in carrier molecules bound to mAbs to further improve drug delivery and targeting (Fig. 5a). For instance, Ahn et al. developed an anti-tissue factor (TF) antibody fragment-antigen binding (Fab') conjugated to polymeric micelles containing an active complex of oxaliplatin, (1,2diaminocyclohexane)platinum(II) (DACHPt) (DACHPt/m, Fig. 5b) [77]. DACHPt/m was formed through maleimide-thiol conjugation and was designed to selectively deliver platinum drugs to pancreatic tumours. Notably, DACHPt/m demonstrated rapid cellular internalisation, resulting in enhanced in vitro cytotoxicity and effectively inhibiting the growth of pancreatic tumour xenografts in vivo, surpassing both non-targeted micelles and free drugs. Furthermore, Zalba et al. developed oxaliplatin (L-OH)-loaded liposomes linked to either whole cetuximab (CTX) or CTX-Fab’ fragments to their surface (Fig. 5c, d) [78]. In EGFR-overexpressing cell lines, targeted liposomes achieved up to threefold higher intracellular drug delivery compared to non-targeted liposomes. When tested in a colorectal cancer (CRC) xenograft model, these ADCs significantly enhanced drug delivery, with CTX-Fab’ L-OH-liposomes outperforming CTX-mAb L-OH-liposomes. This, in turn, was more effective than non-targeted liposomes and free drug treatment in mice with CRC. In addition, a novel ADC was synthesised incorporating ferritin-based nanoparticles where mAb Ep1 were conjugated to a single ferritin cage (Hft) encapsulating cisplatin [79]. Compared to cisplatin-containing ferritin nanoparticle alone, which were more effective in inhibiting thymidine incorporation in breast carcinoma than in melanoma cells, the HFt-Pt-Ep1 nanoparticle exhibited higher preference for melanoma cells. A similar preference for melanoma was observed in nude mice xenotransplanted with melanoma and breast carcinoma cells. This study identified the specific combinations and stoichiometric relationships between mAbs and nanoparticle protein cages, leading to the loss of tropism for ubiquitously distributed cellular receptors and the acquisition of lineage-selective binding. Moreover, another study developed oxaliplatin-loaded apoferritin conjugated with panitumumab via a polyethylene glycol (PEG) linker, which was designed to specifically target EGFR-overexpressing cell lines [80]. This ADC efficiently released oxaliplatin, inhibited tumour cell proliferation, and exhibited enhanced accumulation in tumour models with high EGFR expression in vivo. Remarkably, these studies have exhibited promising results, demonstrating that immune-nanocarriers can effectively enhance the therapeutic translational potential of ADCs containing platinum drugs. Due to the promising results obtained in these studies, ongoing efforts are currently underway to develop next-generation platinum-based ADCs.

### Platinum resistance

Platinum-resistant cancer patients face limited and often ineffective treatment options, highlighting a significant unmet medical need. Moreover, the prognosis for these patients is generally poor, with lower survival rates compared to those with platinum-sensitive cancers [81]. Approximately 85% of ovarian cancer patients eventually develop resistance after an initial response to the treatment [82]. These patients typically have low response rates to further chemotherapy (< 15%), with a PFS of 3 to 4 months and a median survival of less than 1 year [81]. Mechanistically, cells can prevent cisplatin from reaching and harming DNA by reducing drug uptake, increasing drug efflux, and inactivating the drug through covalent binding to glutathione or metalloproteins [9, 10]. Additionally, resistance involves downstream responses such as altered apoptosis signalling and autophagy. If cisplatin does interact with DNA and cause damage, cells respond by enhancing repair mechanisms within the DNA damage response (DDR) pathways to counteract the effects. Therefore, identifying therapeutic strategies targeting DDR proteins involved in the repair of platinum-induced DNA lesions provides one approach to the development of potential strategies aimed to address platinum resistance and increase clinical benefit. This approach is best illustrated by encompassing inhibitors of key mediators of DNA repair alongside platinum drugs in various recurrent cancers (NCT01237067 and NCT01033292) [83, 84]. Additionally, cyclin dependent kinases (CDKs) are also key regulators of DRR, which led to a phase I study examining the combination of ribocliclib, a CDK4/6 inhibitor, with carboplatin/paclitaxel in patients with recurrent platinum-sensitive ovarian cancer (NCT03056833) [85]. Notably, this combination was deemed to be safe and feasible, with an ORR of 79.3% and the mPFS was 11.4 months. Moreover, histone deacetylase (HDAC) expression was significantly increased in resistant tumours [86]. Based on this, the non-kinase inhibitor of HDAC, quisinostat, was investigated in combination with carboplatin/paclitaxel in the clinic, particularly for recurrent cancer (NCT02948075 and NCT00772798) [87, 88]. Encouragingly, in patients with recurrent platinum-sensitive ovarian cancer, this combination showed significant responses and good tolerability (ORR: 62.2%, mPFS: 11.6 months; *P* < 0.001, and mOS: 40.6 months) [88]. Additionally, the mAb cetuximab in combination with platinum is an effective first-line regimen in recurrent/metastatic HNSCC patients, and subsequent studies examined the addition of patritumab, anti-HER3 mAb, to the cetuximab/platinum regimen (NCT02350712 and NCT02633800) [89, 90]. A phase Ib trial showed patritumab plus cetuximab/platinum was tolerable and active in recurrent and/or metastatic HNSCC [89]. The combination of iniparib with carboplatin and gemcitabine demonstrates notable clinical activity and is well tolerated in platinum-sensitive and -resistant recurrent ovarian cancer, particularly in patients with BRCA mutations (NCT01033123) [91]; however, given that iniparib is no longer considered a true PARPi and its mechanism of action remains unclear [92], the basis of this observed synergy warrants further investigation.

### Effects of scheduling

Administration timing may be relevant in combination design, based on the notion that platinum-induced DNA damage and the activation of the DDR may need to occur first before the introduction of DNA repair inhibitors. This was shown by Li et al. in ovarian cancer models, where sequential administration of carboplatin and cell division cycle 7-related protein kinase (CDC7) inhibitor, XL413, showed synergistic enhancement of apoptosis [93]. Mechanistically, XL413 increases the accumulation of chemotherapy-induced DNA damage by inhibiting HR repair activity and delaying the recovery of DNA DSBs. This observation suggests that variable drug positioning, particularly delayed administration of DNA repair inhibitors following DNA-damaging agents, might increase treatment efficacy. For example, in a phase I/Ib trial, carboplatin was given before olaparib to patients with ovarian, breast, and uterine cancer [84]. Pharmacokinetic data from the trial suggested that administering carboplatin before olaparib may be the preferred treatment schedule to enhance the overall clinical benefit of this combination therapy, as pre-exposure to carboplatin causes intracellular accumulation of olaparib (*P* = 0.013), thereby improving its effective availability within tumour cells. These findings underscore the importance of exploring and optimising treatment scheduling to maximise their efficacy, particularly for future platinum drug combinations.

## Combining platinum-based drugs with immunotherapy

The advent of frontline immunotherapy in clinical trials rapidly changed the treatment landscape, establishing it as the standard of care in some clinical situations [94]. Platinum-based drugs have demonstrated potential to induce an anti-cancer immune response by promoting the recruitment and activation of immune cells [95, 96], thereby enhancing the efficacy of immunotherapies. Thus, numerous efforts have been made to identify effective platinum/immunotherapy combinations, which have recently entered the clinical setting. This approach is particularly appealing to patients with advanced cancer, given their limited life expectancy and the drug-related toxicity associated with other combination chemotherapy regimens.

### Immune checkpoint inhibitors (ICIs)

Immune checkpoint inhibitors (ICIs) such as anti-PD-1/PD-L1 and anti-CTLA-4 antibodies have obtained regulatory approvals across various tumour types and indications. ICIs work by activating the immune system of the body to identify and target cancer cells [97]. In 2011, based on promising results from a clinical trial in melanoma patients, the first ICI therapy ipilimumab, an anti-CTLA-4 mAb, has gained FDA approval [98]. Since then, ipilimumab was tested in numerous clinical trials for use in other cancer types. The subsequent triumphs in clinical trials paved the way for the approval of other anti-PD-1 mAbs, such as pembrolizumab, camrelizumab, sintilimab, toripalimab, and nivolumab, for treating a diverse range of malignancies [99]. Notably, the combination of pembrolizumab with platinum-based regimens has been evaluated in many cancers including NSCLC, SCLC, gastric, gastro-oesophageal junction, ovarian, urinary tract, endometrial, and BTC (NCT02549209, NCT02578680, NCT02580994, NCT02608684, NCT02853305, NCT02954536, NCT03029598, NCT03066778, NCT03664024, NCT03675737, NCT03582475, and NCT04003636) [100–111]. In the phase III KEYNOTE-189 trial of previously untreated metastatic NSCLC patients, the addition of pembrolizumab to platinum/pemetrexed chemotherapy improved efficacy outcomes with manageable toxicity [100]. Amongst 57 patients who completed 35 cycles of pembrolizumab/chemotherapy, the ORR was 86.0% and the 3-year OS rate was 71.9%. The benefit of pembrolizumab correlated with PD-L1 expression levels, with greater efficacy in patients with a tumour proportion score (TPS) $\ge$ 50%. In the intent-to-treat (ITT) population, the 5-year OS rate was approximately 20% with pembrolizumab plus chemotherapy compared to 11% with placebo plus chemotherapy, with higher survival observed in patients with TPS $\ge$ 50% (29.6% v 21.4%). Notably, in patients with advanced endometrial cancer, the addition of pembrolizumab to carboplatin/paclitaxel was found to improve ORR and was well tolerated compared to the placebo/chemotherapy group (ORR: 74.4%; *P* = 0.001, and mPFS: 10.6 months) [108]. In addition, results from phase III trials in advanced BTC patients showed that the addition of pembrolizumab to cisplatin/gemcitabine revealed an improvement in OS compared to cisplatin/gemcitabine alone without any new safety signals (mOS: 12·7 vs. 10·9 months; one-sided *P* = 0.0034 [significance threshold *P* = 0.02]) [110]. Additionally, in the KEYNOTE-859 phase III trial assessing patients with locally advanced or metastatic HER2-negative gastric or gastro-oesophageal junction adenocarcinoma, adding pembrolizumab to platinum/chemotherapy treatment significantly improved OS with a manageable toxicity profile compared to the placebo/chemotherapy group [111]. As such, these studies support the concept of adding pembrolizumab to platinum-based regimens as first-line treatment for various metastatic/advanced cancers. Despite the positive clinical responses observed, several studies demonstrated that these combinations did not significantly improve efficacy or provide benefit beyond chemotherapy alone in patients with untreated extensive-stage SCLC [101], recurrent platinum-resistant ovarian cancer patients [105], or advanced urothelial carcinoma patients [106]. This treatment also did not improve the durability of response in patients with platinum-resistant recurrent ovarian cancer compared to platinum chemotherapy alone, although the combination regimen was well tolerated with no discontinuations due to treatments-related toxicity [105].

In the CANTABRICO phase III trial, anti-PD-L1 durvalumab, was examined in combination with platinum/etoposide regimen in extensive-stage SCLC patients (NCT04712903 and EudraCT 2020–002328-35) [112]. This study demonstrated good clinical benefits with favourable safety profile (ORR: 51.5%; mPFS: 6.1 months and 6-month PFS rate: 50.2%). In another phase III trial, durvalumab with cisplatin/gemcitabine significantly showed improvements compared to the placebo/chemotherapy group in patients with BTC (24-month OS rate: 24.9% vs. 10.4% and ORR: 26.7% vs. 18.7%; NCT03875235) [113]. In another intriguing approach, anti-CTLA-4 mAb, ipilimumab, was added to nivolumab/platinum-doublet chemotherapy regimens where this combination was found effective and tolerable as a first-line treatment of advanced/metastatic NSCLC (NCT02659059) [114]. Another anti-CTLA-4 mAb, tremelimumab, was added to durvalumab/cisplatin/5-FU treatment where manageable safety and anti-tumour activity in patients with advanced or metastatic ESCC were shown (OR: 37.5%; mPFS: 3.75 months and mOS: 9.69 months; NCT02658214) [115], warranting further investigation in randomised trials.

Sintilimab was also examined in combination with a platinum-based regimen in patients with advanced or metastatic NSCLC (NCT02937116 and NCT03629925) [116, 117]. In these patients, this combination showed good clinical efficacy (ORR: 68.4%, and mPFS: 11.4 months), with an acceptable safety profile [116]. Other trials also examined toripalimab (NCT04144608) [118], and nivolumab (NCT02944396) [119], in combination with platinum-based regimens in NSCLC patients. Promisingly, the addition of toripalimab to a platinum-based regimen demonstrated robust anti-tumour activity with good tolerability in patients with potentially resectable NSCLC [118]. Of note, the combination of cemiplimab-rwlc, mAb targeting PD-1, in combination with platinum-based chemotherapy has gained FDA approval as first-line treatment for adult patients with advanced NSCLC [120].

### Oncolytic viruses, cytokines, and cancer vaccines

Other alternative immunotherapeutic approaches include oncolytic viruses, cytokines, and cancer vaccines, which represent potent approaches for treating certain aggressive and refractory cancers. For example, pelareorep (REOLYSIN), is an investigational novel oncolytic virus composed of a live, replication-competent, Reovirus Type 3 Dearing strain in a proprietary formulation [121]. Preclinical data demonstrated that pelareorep induces antineoplastic activity across various cancers types, particularly in cells with an activated RAS-signalling pathway [122]. In a randomised phase II trial, the combination of pelareorep with carboplatin/paclitaxel was safe but did not improve PFS in patients with metastatic pancreatic adenocarcinoma (NCT01280058) [123]. However, in another study, the addition of pelareorep to the carboplatin/paclitaxel regimen was both safe and showed promising clinical activity in patients with advanced malignant melanoma, with a mPFS of 5.2 months, mOS of 10.9 months and 1-year OS rate of 43% (NCT00984464) [124], warranting further randomised phase III trials. The tumour necrosis factor (TNF) is a pivotal proinflammatory cytokine that influences various aspects of the immune response [125]. The safety, efficacy, and pharmacodynamic effects of the addition of certolizumab, a TNF inhibitor, to cisplatin/pemetrexed regimen was evaluated in stage IV lung adenocarcinoma patients (NCT02120807) [126]. This treatment modality was well tolerated and the mPFS was 7.1 months.

In addition to cytokines, cancer vaccines are emerging as promising immunotherapies, demonstrating a level of therapeutic efficacy that surpasses or equals that of other treatments in certain contexts, which is considered high relative to current standards of care [127]. For example, dendritic cell vaccination is a safe immunotherapeutic approach that works by harnessing the body’s own immune system. It involves isolating dendritic cells from a patient, loading them with tumour antigens, and then reinfusing them into the patient to elicit both immunological and clinical responses in solid tumour patients. These antigen-presenting cells stimulate T cells to recognise and attack tumour cells, thereby potentially reducing tumour growth and improving patient outcomes. In a phase I/II study, the addition of dendritic cell vaccination to a carboplatin/paclitaxel regimen was safe and tolerable in patients with metastatic endometrial cancer (NCT04212377) [128]. Subsequently, the dendritic cell-based immunotherapy, DCVAC/OvCa, was combined with carboplatin/gemcitabine regimen in a phase II trial to evaluate their safety and efficacy in platinum-sensitive ovarian cancer (NCT02107950) [129]. DCVAC/OvCa combined with chemotherapy significantly prolonged mOS compared to the placebo/chemotherapy group (35.5 vs. 22.1 months; *P* = 0.003), and had a favourable safety profile. Moreover, the field of oncolytic virotherapy is progressing, as evidenced by a phase III clinical trial investigating the investigational oncolytic virus olvimulogene nanivacirepvec (Olvi-Vec) administered in combination with platinum-doublet chemotherapy and bevacizumab for the treatment of platinum-resistant or refractory ovarian cancer (NCT05281471) [130]. This trial highlights the ongoing efforts to combine virotherapy with traditional chemotherapy and immunotherapy modalities in cancer treatment.

## Comparative effectiveness of platinum-based combination strategies in different malignancies

Although platinum combinations are widely used in multiple malignancies, clinical outcomes vary significantly depending on cancer type and partner drugs. In advanced NSCLC, platinum combinations with immune checkpoint inhibitors or anti-angiogenic therapies have demonstrated improved clinical outcomes. For example, pembrolizumab combined with cisplatin and pemetrexed showed an ORR of 86% and a 3-year OS of 71.9% in previously untreated metastatic non-squamous NSCLC (NCT02578680), illustrating the potential of immunotherapy-platinum combinations [100]. In extensive-stage SCLC, platinum-etoposide remains a standard regimen, with immune checkpoint inhibitors further improving outcomes. Pembrolizumab combined with platinum-etoposide demonstrated an ORR of 70.6% and a 12-month PFS of 13.6%, whilst durvalumab showed an ORR of 51.5% and mPFS of 6.1 months (NCT03066778 and NCT04712903) [103, 112].

In ovarian cancer, carboplatin-based doublets remain the cornerstone of therapy, with paclitaxel-carboplatin showing robust survival outcomes across multiple phase III trials (mPFS 16.8–20.7 months, mOS up to 57.4 months; NCT00326456) [131]. Substituting paclitaxel with PLD has yielded even longer survival (mPFS 19.0 months, mOS 61.6 months), especially in partially platinum-sensitive patients. Early-phase trials incorporating agents like veliparib or gemcitabine have demonstrated response rates up to 45%, and mirvetuximab soravtansine has shown an ORR of 71% and mPFS of 15 months (NCT02606305) [73]. In HNSCC, platinum-based regimens combined with cetuximab remain a standard treatment, with the EXTREME regimen achieving mOS of 10.1 months and PFS of 5.6 months (NCT00122460) [132]. The TPEx regimen further improved mOS to 14.5 months (NCT02268695) [63]. Encouragingly, combinations with veliparib or HER-targeting agents like panitumumab and patritumab demonstrated mOS up to 13.5 months (NCT00454779, NCT02633800) [90, 133].

In TNBC, platinum-based combinations have been linked to improved pCR rates, particularly in early-stage disease. For instance, neoadjuvant paclitaxel plus carboplatin achieved a pCR of 52% and a 36-month EFS of 79% in the PARTNER trial (NCT03150576) [58, 59]. Adding olaparib yielded a pCR of 51% and slightly improved 36-month EFS (80%) and overall survival (90%). Other regimens, such as carboplatin with docetaxel or with cyclophosphamide and doxorubicin, showed pCR rates between 52 and 55% (NCT02413320) [27]. In the metastatic setting, platinum-PARPi combinations have demonstrated promising activity in TNBC, including those without BRCA mutations. For example, veliparib with carboplatin and paclitaxel resulted in a mPFS of 16.6 months in TNBC patients (NCT02163694) [57]. Finally, in BTC, cisplatin-gemcitabine remains the standard first-line therapy, with mOS ranging from 12.6 to 13.5 months and mPFS of 5.5–7.4 months (NCT02182778) [30]. Enhancing this regimen with nab-paclitaxel has resulted in mOS improvements up to 19.2 months and high disease control rates (84%) (NCT02392637) [33]. In contrast, carboplatin-paclitaxel remains the backbone of first-line therapy in ovarian cancer, with phase III trials showing mOS up to 44.8 months and mPFS of 16.2 months (NCT00028743) [134].

## Other therapeutic strategies for platinum-based drugs

### Combining platinum-based drugs with stem cell therapies

Besides the described treatment modalities, stem cell therapies, including stem cell transplants, in combination with platinum regimens, represent a promising approach for treating certain aggressive and refractory cancers. These have been the focus of phase III clinical trials in patients with relapsed hodgkin's lymphoma (NCT00025636) [135, 136], and men with previously untreated germ cell cancer (NCT00003941) [137]. Notably, in patients with relapsed or refractory germ cell tumours, the inclusion of stem cell transplants in the cisplatin/cytotoxic regimen demonstrated clinical benefits, with a 2-year PFS rate of 67% and a 2-year OS rate of 72% (NCT02375204) [138], indicating potential for regulatory approval and broader clinical application, albeit with the need for careful patient selection and management due to associated risks and side effects.

### Dual-drug codelivery nanosystems

Since the approval of nanotherapeutics that are commercially available, such as Abraxane^®^ (nab-paclitaxel), Doxil^®^ (liposomal doxorubicin), Onivyde^®^ (liposomal irinotecan), and Vyxeos^®^ (daunorubicin and cytarabine liposome), there has been a growing interest in nanocarrier approaches to deliver therapeutic agents. Nanocarriers offer several advantages, including enhancing the water solubility of poorly soluble drugs, prolonging their circulation time in the blood, and facilitating drug targeting to tumours [139]. This targeted delivery increases drug availability within tumour cells whilst mitigating the toxic and off-target side effects typically associated with traditional chemotherapy. The benefits of encapsulating platinum drugs in nanoparticles to reduce side effects without compromising efficacy have been demonstrated in tumour-bearing mice and preclinical cancer models [140]. Also, the tumour-localised drug delivery strategies exhibit benefits for preventing local tumour recurrence [141]. Several nanocarriers for cisplatin have entered clinical trials. For example, Lipoplatin, a liposomal cisplatin formulation, which has reached phase III trials and demonstrated excellent encouraging anti-cancer efficacy in several tumour types, including lung, colon, gastric, and prostate cancers [142], although clinical adoption remains limited. Another formulation, NC-6004, is a poly(glutamic acid) (PGlu)-based polymeric micelle containing cisplatin. In early-phase trials involving patients with advanced solid tumours, NC-6004 demonstrated reduced nephrotoxicity compared to cisplatin alone [143]. It has been further investigated in clinical studies in combination with gemcitabine [144–146] and pembrolizumab [147]. Despite mixed clinical outcomes, these formulations highlight the ongoing efforts and challenges in optimising nanocarrier-based platinum drug delivery systems. In preclinical studies, various nanocarriers have also been utilised for dual-drug codelivery of cisplatin/paclitaxel including telodendrimers [148], polymeric micelles [149], and nanostructured lipid carriers [150, 151] as well as cisplatin/doxorubicin combinations using hyaluronic acid micelles [152], and mesoporous silica NPs (MSNs) [153]. These studies demonstrated that dual-drug nanocarriers enhance pharmacokinetics by improving solubility, protecting drugs from rapid degradation, enabling extended release, bypassing first-pass metabolism, and providing targeted delivery.

### Multi-targeted platinum compounds

In contrast to the serendipitous discovery of the first-in-class drug, cisplatin, the subsequent development of platinum metallodrugs has relied heavily on rational drug design. A thorough analysis of the mechanism of action and adverse effects linked with the first-generation drug has enabled the rectification of these issues in the development of subsequent agents. Amidst the evolving landscape of reformed and newly developed platinum drugs, multi-targeted platinum compounds have garnered considerable attention for their potential in cancer-specific therapy [154]. This focus has spurred extensive research into leveraging cellular targets beyond DNA for therapeutic interventions involving platinum compounds. For example, Fronik et al. developed a triple-action platinum(IV) prodrug, designed for tumour targeting via maleimide-mediated albumin binding, also for release of the immunomodulatory ligand 1-methyl-d-tryptophan (1-MDT) [155]. Structure–activity relationship analysis unexpectedly revealed that the mode of 1-MDT conjugation significantly influences the prodrug's reducibility and activation. This, in turn, affects ligand release, pharmacokinetics, immunomodulatory efficiency, and anti-cancer activity both in vitro and in vivo. The use of albumin-targeted, multi-functional platinum(IV) prodrugs represents a promising strategy to enhance the intracellular delivery of low-permeability bioactive ligands like 1-MDT and to improve their selective accumulation in tumours, thereby enabling tumour-specific therapy supported by a modulated immune microenvironment.

**Fig. 6:**
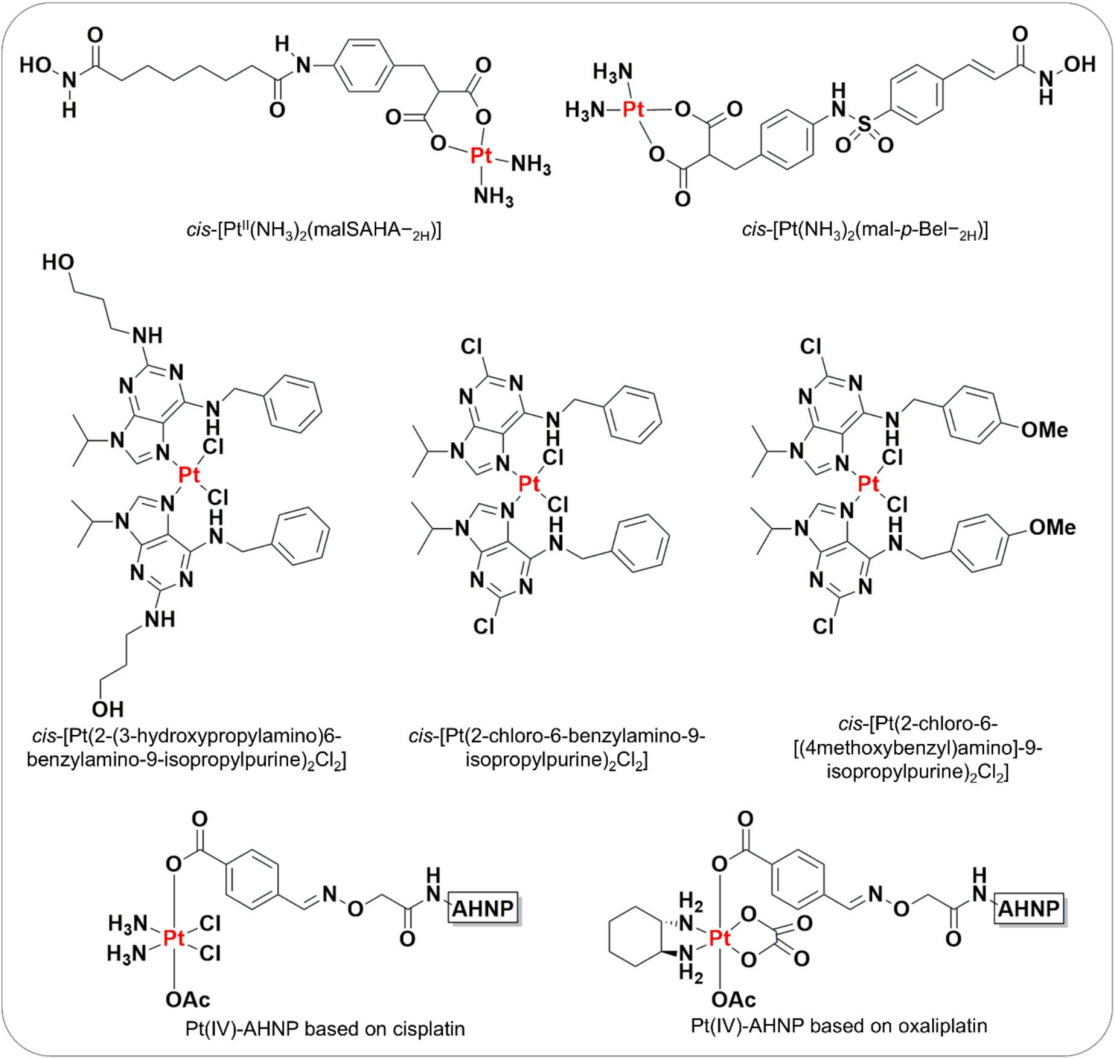
Structures of investigational multi-targeted platinum compounds

Enzymes, integral to nearly all physiological and pathophysiological processes, have long been recognised as promising drug targets. Several studies have thus developed dual-functioning platinum complexes, incorporating a HDAC inhibitor (HDACi) within the platinum framework. For instance, *cis*-[Pt^II^(NH_3_)_2_(malSAHA − _2H_)] (Pt-malSAHA) comprising both a cisplatin core and the HDACi, SAHA, has demonstrated DNA binding properties and HDAC inhibitory activity (Fig. 6) [156]. Remarkably, Pt-malSAHA exhibited potent cytotoxicity across various cancer cell lines, including ovarian, colon, lung, and breast cancer cells, with notably enhanced cancer selectivity over normal cells. Subsequently, Belinostat, a second-generation analogue of SAHA was combined within a platinum(II) framework, to develop *cis*-[Pt^II^(NH_3_)_2_(mal-*p*-Bel − _2H_)] (Pt-malBel) (Fig. 6) [157], showing similar cytotoxicity to Pt-malSAHA in A2780 ovarian cells and significant potency against cisplatin-resistant A2780cisR ovarian cancer cells. Additionally, two novel trans-platinum(II) complexes incorporating the HDACi valproic acid (VPA), named *trans*-[Pt(VPA − _1H_)_2_(NH_3_)(py)] and *trans*-[Pt(VPA − _1H_)_2_(py)_2_], where py is pyridine were developed [158]. These complexes showed only marginally enhanced cytotoxicity against A2780 and A2780cisR cells compared to cisplatin.

In addition to HDACi, new complexes combining a CDK inhibitor, bohemine or its derivatives, within a platinum(II) framework were developed, namely, *cis*-[Pt(2-(3-hydroxypropylamino)6-benzylamino-9-isopropylpurine)_2_Cl_2_], *cis*-[Pt(2-chloro-6-benzylamino-9-isopropylpurine)_2_Cl_2_], and *cis*-[Pt(2-chloro-6-[(4methoxybenzyl)amino]−9-isopropylpurine)_2_Cl_2_] (Fig. 6), demonstrating cytotoxicity comparable to or greater than that of cisplatin against ovarian cells [159]. Interestingly, the increased cytotoxicity was not attributed to CDK inhibitory activity, as CDK inhibition was lost when bohemine was complexed with the platinum(II) core. However, the complexes induced significant DNA platination, suggesting DNA binding as their primary mechanism of action. In another study, Wong et al. developed platinum(IV)-peptide conjugates using a cisplatin or oxaliplatin core, incorporating an anti-HER2/neu peptide (NH2-Tyr-Cys-Asp-Gly-Phe-Tyr-Ala-Cys-Tyr-Met-Asp-Val-Gly-Gly-Lys-Lys(aminooxy)-CONH2, or ANHP) (Fig. 6) [160]. These complexes demonstrated cytotoxicity comparable to cisplatin and oxaliplatin, and showed selective targeting for HER2-overexpressing NCI-N87 gastric cancer cells and BT-474 breast ductal carcinoma cells, both of which are resistant to apoptosis. Importantly, these platinum complexes exhibited enhanced selectivity for cancerous cells over normal cells, with their accumulation in HER2 cancer cells facilitated by the HER2-targeting peptide ligand. Overall, these innovative approaches to drug design have yielded new families of platinum metallodrugs, potentially mitigating the systemic toxicities associated with contemporary chemotherapeutics and addressing resistance issues.

## Conclusion and future perspectives

In conclusion, platinum-based combinations with various drug classes have shown promising clinical responses in randomised studies across various cancer types, surpassing the efficacy of traditional single-drug regimens. However, the limitations of established platinum/cytotoxic combinations, such as toxicity and drug resistance issues, highlight the necessity for innovative approaches. Newer platinum combinations, such as those with targeted therapies and immunotherapies, have demonstrate improved tolerability. Notably, combining platinum drugs with DDR inhibitors shows promise in targeting resistant cancers, and pairing platinum drugs with emerging treatment modalities likes oncolytic viruses, cancer vaccines, and cytokines holds significant potential. Whilst the potential benefits are substantial, these studies highlight the need to select platinum combinations for investigation in appropriate disease settings and patient populations to attain clinical benefit. As such, ongoing research efforts are focussed on optimising treatment regimens and identifying predictive biomarkers of therapy response to refine patient selection and maximise clinical benefits. Advances in molecular profiling and personalised medicine are likely to lead to more precise targeting of cancer cells, reducing the risk of toxicity and increasing the efficacy of treatment.

Whilst platinum-based ADCs and multi-targeted platinum compounds have not yet entered clinical trials, promising preclinical data suggest their potential clinical application. With extensive ongoing efforts to develop next-generation platinum compounds by identifying new targets and enhancing their pharmacological properties, it is likely that multi-targeted platinum drugs will reach the clinical stage in the near future. Additionally, advancements in drug delivery systems could improve the bioavailability and selectivity of platinum compounds through dual-drug delivery strategies, further enhancing their therapeutic potential [161]. Another promising avenue for future research is the integration of artificial intelligence (AI) in drug discovery and precision oncology. AI and machine learning models can facilitate the identification of optimal platinum-based combinations, predict patient responses using multi-omics data, and refine clinical trial designs, accelerating the development of more effective therapies [162]. Looking ahead, platinum-based combination therapies are poised to play a pivotal role in cancer management, offering more effective and less toxic treatment options. By leveraging emerging technologies, refining patient stratification, and advancing drug formulations, platinum-based chemotherapy is set to drive significant progress in oncology treatment.

## Supplementary Information

Below is the link to the electronic supplementary material.

Supplementary file1 (DOCX 528 KB)

## Data Availability

No datasets were generated or analysed during the current study.

## References

[1] Siegel RL, Miller KD, Wagle NS, Jemal A. Cancer statistics, 2023. CA Cancer J Clin. 2023;73(1):17–48. 10.3322/caac.21763. 36633525 10.3322/caac.21763

[2] Ferlay J, Colombet M, Soerjomataram I, Parkin DM, Piñeros M, Znaor A, et al. Cancer statistics for the year 2020: an overview. Int J Cancer. 2021;149(4):778–89. 10.1002/ijc.33588. 10.1002/ijc.33588 33818764

[3] Franz KJ, Metzler-Nolte N. Introduction: metals in medicine. Chem Rev. 2019;119(2):727–9. 10.1021/acs.chemrev.8b00685. 30990707 10.1021/acs.chemrev.8b00685

[4] Anthony EJ, Bolitho EM, Bridgewater HE, Carter OWL, Donnelly JM, Imberti C, et al. Metallodrugs are unique: opportunities and challenges of discovery and development. Chem Sci. 2020;11(48):12888–917. 10.1039/D0SC04082G. 34123239 10.1039/d0sc04082g PMC8163330

[5] Yousuf I, Bashir M, Arjmand F, Tabassum S. Advancement of metal compounds as therapeutic and diagnostic metallodrugs: current frontiers and future perspectives. Coord Chem Rev. 2021;445: 214104. 10.1016/j.ccr.2021.214104.

[6] Rosenberg B, Vancamp L, Trosko JE, Mansour VH. Platinum compounds: a new class of potent antitumour agents. Nature. 1969;222(5191):385–6. 10.1038/222385a0. 5782119 10.1038/222385a0

[7] Dasari S, Bernard Tchounwou P. Cisplatin in cancer therapy: molecular mechanisms of action. Eur J Pharmacol. 2014;740:364–78. 10.1016/j.ejphar.2014.07.025. 25058905 10.1016/j.ejphar.2014.07.025 PMC4146684

[8] Tang C, Livingston MJ, Safirstein R, Dong Z. Cisplatin nephrotoxicity: new insights and therapeutic implications. Nat Rev Nephrol. 2023;19(1):53–72. 10.1038/s41581-022-00631-7. 36229672 10.1038/s41581-022-00631-7

[9] Galluzzi L, Senovilla L, Vitale I, Michels J, Martins I, Kepp O, et al. Molecular mechanisms of cisplatin resistance. Oncogene. 2012;31(15):1869–83. 10.1038/onc.2011.384. 21892204 10.1038/onc.2011.384

[10] Shen DW, Pouliot LM, Hall MD, Gottesman MM. Cisplatin resistance: a cellular self-defense mechanism resulting from multiple epigenetic and genetic changes. Pharmacol Rev. 2012;64(3):706–21. 10.1124/pr.111.005637. 22659329 10.1124/pr.111.005637 PMC3400836

[11] Calvert AH, Harland SJ, Newell DR, Siddik ZH, Jones AC, McElwain TJ, et al. Early clinical studies with cis-diammine-1,1-cyclobutane dicarboxylate platinum II. Cancer Chemother Pharmacol. 1982;9(3):140–7. 10.1007/bf00257742. 6761010 10.1007/BF00257742

[12] Alcindor T, Beauger N. Oxaliplatin: a review in the era of molecularly targeted therapy. Curr Oncol. 2011;18(1):18–25. 10.3747/co.v18i1.708. 21331278 10.3747/co.v18i1.708 PMC3031353

[13] Johnstone TC, Suntharalingam K, Lippard SJ. The next generation of platinum drugs: targeted Pt(II) agents, nanoparticle delivery, and Pt(IV) prodrugs. Chem Rev. 2016;116(5):3436–86. 10.1021/acs.chemrev.5b00597. 26865551 10.1021/acs.chemrev.5b00597 PMC4792284

[14] Choy H, Park C, Yao M. Current status and future prospects for satraplatin, an oral platinum analogue. Clin Cancer Res. 2008;14(6):1633–8. 10.1158/1078-0432.Ccr-07-2176. 18347164 10.1158/1078-0432.CCR-07-2176

[15] Bhargava A, Vaishampayan UN. Satraplatin: leading the new generation of oral platinum agents. Expert Opin Investig Drugs. 2009;18(11):1787–97. 10.1517/13543780903362437. 19888874 10.1517/13543780903362437 PMC3856359

[16] Emran TB, Shahriar A, Mahmud AR, Rahman T, Abir MH, Siddiquee MFR, et al. Multidrug resistance in cancer: understanding molecular mechanisms, immunoprevention and therapeutic approaches. Front Oncol. 2022. 10.3389/fonc.2022.891652. 35814435 10.3389/fonc.2022.891652 PMC9262248

[17] Mokhtari RB, Homayouni TS, Baluch N, Morgatskaya E, Kumar S, Das B, et al. Combination therapy in combating cancer. Oncotarget. 2017;8(23):38022–43. 10.18632/oncotarget.16723. 28410237 10.18632/oncotarget.16723 PMC5514969

[18] Lehár J, Krueger AS, Avery W, Heilbut AM, Johansen LM, Price ER, et al. Synergistic drug combinations tend to improve therapeutically relevant selectivity. Nat Biotechnol. 2009;27(7):659–66. 10.1038/nbt.1549. 19581876 10.1038/nbt.1549 PMC2708317

[19] Lopez JS, Banerji U. Combine and conquer: challenges for targeted therapy combinations in early phase trials. Nat Rev Clin Oncol. 2017;14(1):57–66. 10.1038/nrclinonc.2016.96. 27377132 10.1038/nrclinonc.2016.96 PMC6135233

[20] Muhammad N, Guo Z. Metal-based anticancer chemotherapeutic agents. Curr Opin Chem Biol. 2014;19:144–53. 10.1016/j.cbpa.2014.02.003. 24608084 10.1016/j.cbpa.2014.02.003

[21] Ghosh S. Cisplatin: the first metal based anticancer drug. Bioorg Chem. 2019;88: 102925. 10.1016/j.bioorg.2019.102925. 31003078 10.1016/j.bioorg.2019.102925

[22] Karam A, Ledermann JA, Kim JW, Sehouli J, Lu K, Gourley C, et al. Fifth ovarian cancer consensus conference of the gynecologic cancer interGroup: first-line interventions. Ann Oncol. 2017;28(4):711–7. 10.1093/annonc/mdx011. 28327917 10.1093/annonc/mdx011

[23] Planchard D, Popat S, Kerr K, Novello S, Smit EF, Faivre-Finn C, et al. Metastatic non-small cell lung cancer: ESMO Clinical Practice Guidelines for diagnosis, treatment and follow-up. Ann Oncol. 2018;29:192–237. 10.1093/annonc/mdy275. 10.1093/annonc/mdy275 30285222

[24] Hanna N, Johnson D, Temin S Jr, Brahmer SBJ, Ellis PM, et al. Systemic therapy for stage IV non–small-cell lung cancer: American society of clinical oncology clinical practice guideline update. J Clin Oncol. 2017;35(30):3484–515. 10.1200/jco.2017.74.6065. 28806116 10.1200/JCO.2017.74.6065

[25] Ma WW, Zhu M, Lam ET, Diamond JR, Dy GK, Fisher GA, et al. A phase I pharmacokinetic and safety study of Paclitaxel Injection Concentrate for Nano-dispersion (PICN) alone and in combination with carboplatin in patients with advanced solid malignancies and biliary tract cancers. Cancer Chemother Pharmacol. 2021;87(6):779–88. 10.1007/s00280-021-04235-z. 33634324 10.1007/s00280-021-04235-z

[26] Gibson JM, Alzghari S, Ahn C, Trantham H, La-Beck NM. The role of pegylated liposomal doxorubicin in ovarian cancer: a meta-analysis of randomized clinical trials. Oncologist. 2013;18(9):1022–31. 10.1634/theoncologist.2013-0126. 23881990 10.1634/theoncologist.2013-0126 PMC3780634

[27] Sharma P, Kimler BF, O’Dea A, Nye LE, Wang YY, Yoder R, et al. Results of randomized phase II trial of neoadjuvant carboplatin plus docetaxel or carboplatin plus paclitaxel followed by AC in stage I-III triple-negative breast cancer (NCT02413320). J Clin Oncol. 2019;37(15_suppl):516–516. 10.1200/JCO.2019.37.15_suppl.516.

[28] Mahner S, Meier W, du Bois A, Brown C, Lorusso D, Dell’Anna T, et al. Carboplatin and pegylated liposomal doxorubicin versus carboplatin and paclitaxel in very platinum-sensitive ovarian cancer patients: results from a subset analysis of the CALYPSO phase III trial. Eur J Cancer. 2015;51(3):352–8. 10.1016/j.ejca.2014.11.017. 25534295 10.1016/j.ejca.2014.11.017

[29] McNamara MG, Bridgewater J, Palmer DH, Faluyi O, Wasan H, Patel A, et al. A Phase Ib Study of NUC-1031 in combination with cisplatin for the first-line treatment of patients with advanced biliary tract cancer (ABC-08). Oncologist. 2021;26(4):e669–78. 10.1002/onco.13598. 33210382 10.1002/onco.13598 PMC8018303

[30] Ioka T, Kanai M, Kobayashi S, Sakai D, Eguchi H, Baba H, et al. Randomized phase III study of gemcitabine, cisplatin plus S-1 versus gemcitabine, cisplatin for advanced biliary tract cancer (KHBO1401- MITSUBA). J Hepatobiliary Pancreat Sci. 2023;30(1):102–10. 10.1002/jhbp.1219. 35900311 10.1002/jhbp.1219 PMC10086809

[31] Wilbur HC, Durham JN, Lim SJ, Purtell K, Bever KM, Laheru DA, et al. Gemcitabine, docetaxel, capecitabine, cisplatin, irinotecan as first-line treatment for metastatic pancreatic cancer. Cancer Res Commun. 2023;3(8):1672–7. 10.1158/2767-9764.Crc-23-0230. 37645623 10.1158/2767-9764.CRC-23-0230 PMC10461640

[32] Christenson ES, Lim SJ, Durham J, De Jesus-Acosta A, Bever K, Laheru D, et al. Cell-free DNA predicts prolonged response to multi-agent chemotherapy in pancreatic ductal adenocarcinoma. Cancer Res Commun. 2022;2(11):1418–25. 10.1158/2767-9764.Crc-22-0343. 36970054 10.1158/2767-9764.CRC-22-0343 PMC10035498

[33] Shroff RT, Javle MM, Xiao L, Kaseb AO, Varadhachary GR, Wolff RA, et al. Gemcitabine, cisplatin, and nab-paclitaxel for the treatment of advanced biliary tract cancers: a phase 2 clinical trial. JAMA Oncol. 2019;5(6):824–30. 10.1001/jamaoncol.2019.0270. 30998813 10.1001/jamaoncol.2019.0270 PMC6567834

[34] Gladieff L, Ferrero A, De Rauglaudre G, Brown C, Vasey P, Reinthaller A, et al. Carboplatin and pegylated liposomal doxorubicin versus carboplatin and paclitaxel in partially platinum-sensitive ovarian cancer patients: results from a subset analysis of the CALYPSO phase III trial. Ann Oncol. 2012;23(5):1185–9. 10.1093/annonc/mdr441. 21976386 10.1093/annonc/mdr441

[35] Spigel DR, Hainsworth JD, Shipley DL, Mekhail TM, Zubkus JD, Waterhouse DM, et al. Amrubicin and carboplatin with pegfilgrastim in patients with extensive stage small cell lung cancer: a phase II trial of the Sarah Cannon Oncology Research Consortium. Lung Cancer. 2018;117:38–43. 10.1016/j.lungcan.2018.01.007. 29496254 10.1016/j.lungcan.2018.01.007

[36] Grossi F, Jaśkiewicz P, Ferreira M, Czyżewicz G, Kowalski D, Ciuffreda L, et al. Oral vinorelbine and cisplatin as first-line therapy for advanced squamous NSCLC patients: a prospective randomized international phase II study (NAVoTrial 03). Ther Adv Med Oncol. 2021. 10.1177/17588359211022905. 34349841 10.1177/17588359211022905 PMC8287271

[37] Pérez-Ramírez C, Cañadas-Garre M, Alnatsha A, Villar E, Delgado JR, Faus-Dáder MJ, et al. Pharmacogenetic predictors of toxicity to platinum based chemotherapy in non-small cell lung cancer patients. Pharmacol Res. 2016;111:877–84. 10.1016/j.phrs.2016.08.002. 27498158 10.1016/j.phrs.2016.08.002

[38] de Jong C, Herder GJM, Deneer VHM. Genetic variants as predictors of toxicity and response in patients with non-small cell lung cancer undergoing first-line platinum-based chemotherapy: design of the multicenter PGxLUNG study. Thorac Cancer. 2020;11(12):3634–40. 10.1111/1759-7714.13683. 33073546 10.1111/1759-7714.13683 PMC7705630

[39] Min H-Y, Lee H-Y. Molecular targeted therapy for anticancer treatment. Exp Mol Med. 2022;54(10):1670–94. 10.1038/s12276-022-00864-3. 36224343 10.1038/s12276-022-00864-3 PMC9636149

[40] Dagher R, Cohen M, Williams G, Rothmann M, Gobburu J, Robbie G, et al. Approval summary: imatinib mesylate in the treatment of metastatic and/or unresectable malignant gastrointestinal stromal tumors. Clin Cancer Res. 2002;8(10):3034–8. 12374669

[41] Du Z, Lovly CM. Mechanisms of receptor tyrosine kinase activation in cancer. Mol Cancer. 2018;17(1):58. 10.1186/s12943-018-0782-4. 29455648 10.1186/s12943-018-0782-4 PMC5817791

[42] Meng X, Gao JZ, Gomendoza SMT, Li JW, Yang S. Recent advances of WEE1 inhibitors and statins in cancers with p53 mutations. Front Med (Lausanne). 2021;8: 737951. 10.3389/fmed.2021.737951. 34671620 10.3389/fmed.2021.737951 PMC8520942

[43] Hirai H, Iwasawa Y, Okada M, Arai T, Nishibata T, Kobayashi M, et al. Small-molecule inhibition of Wee1 kinase by MK-1775 selectively sensitizes p53-deficient tumor cells to DNA-damaging agents. Mol Cancer Ther. 2009;8(11):2992–3000. 10.1158/1535-7163.Mct-09-0463. 19887545 10.1158/1535-7163.MCT-09-0463

[44] Oza AM, Estevez-Diz M, Grischke EM, Hall M, Marmé F, Provencher D, et al. A Biomarker-enriched, randomized phase II trial of adavosertib (AZD1775) plus paclitaxel and carboplatin for women with platinum-sensitive TP53-mutant ovarian cancer. Clin Cancer Res. 2020;26(18):4767–76. 10.1158/1078-0432.Ccr-20-0219. 32611648 10.1158/1078-0432.CCR-20-0219

[45] Jin H, Wang L, Bernards R. Rational combinations of targeted cancer therapies: background, advances and challenges. Nat Rev Drug Discov. 2023;22(3):213–34. 10.1038/s41573-022-00615-z. 36509911 10.1038/s41573-022-00615-z

[46] Garuti L, Roberti M, Bottegoni G. Multi-kinase inhibitors. Curr Med Chem. 2015;22(6):695–712. 10.2174/0929867321666141216125528. 25511779 10.2174/0929867321666141216125528

[47] Curtin NJ, Szabo C. Poly(ADP-ribose) polymerase inhibition: past, present and future. Nat Rev Drug Discov. 2020;19(10):711–36. 10.1038/s41573-020-0076-6. 32884152 10.1038/s41573-020-0076-6

[48] Mateo J, Lord CJ, Serra V, Tutt A, Balmaña J, Castroviejo-Bermejo M, et al. A decade of clinical development of PARP inhibitors in perspective. Ann Oncol. 2019;30(9):1437–47. 10.1093/annonc/mdz192. 31218365 10.1093/annonc/mdz192 PMC6771225

[49] Cheng H, Zhang Z, Borczuk A, Powell CA, Balajee AS, Lieberman HB, et al. PARP inhibition selectively increases sensitivity to cisplatin in ERCC1-low non-small cell lung cancer cells. Carcinogenesis. 2013;34(4):739–49. 10.1093/carcin/bgs393. 23275151 10.1093/carcin/bgs393 PMC3616665

[50] Prasad CB, Prasad SB, Yadav SS, Pandey LK, Singh S, Pradhan S, et al. Olaparib modulates DNA repair efficiency, sensitizes cervical cancer cells to cisplatin and exhibits anti-metastatic property. Sci Rep. 2017;7(1):12876. 10.1038/s41598-017-13232-3. 28993682 10.1038/s41598-017-13232-3 PMC5634505

[51] Miller K, Tong Y, Jones DR, Walsh T, Danso MA, Ma CX, et al. Cisplatin with or without rucaparib after preoperative chemotherapy in patients with triple negative breast cancer: final efficacy results of Hoosier Oncology Group BRE09-146. J Clin Oncol. 2015;33(15_suppl):1082–1082. 10.1200/jco.2015.33.15_suppl.1082.

[52] Gray HJ, Bell-McGuinn K, Fleming GF, Cristea M, Xiong H, Sullivan D, et al. Phase I combination study of the PARP inhibitor veliparib plus carboplatin and gemcitabine in patients with advanced ovarian cancer and other solid malignancies. Gynecol Oncol. 2018;148(3):507–14. 10.1016/j.ygyno.2017.12.029. 29352572 10.1016/j.ygyno.2017.12.029

[53] Loibl S, O’Shaughnessy J, Untch M, Sikov WM, Rugo HS, McKee MD, et al. Addition of the PARP inhibitor veliparib plus carboplatin or carboplatin alone to standard neoadjuvant chemotherapy in triple-negative breast cancer (BrighTNess): a randomised, phase 3 trial. Lancet Oncol. 2018;19(4):497–509. 10.1016/s1470-2045(18)30111-6. 29501363 10.1016/S1470-2045(18)30111-6

[54] Geyer CE, Sikov WM, Huober J, Rugo HS, Wolmark N, O’Shaughnessy J, et al. Long-term efficacy and safety of addition of carboplatin with or without veliparib to standard neoadjuvant chemotherapy in triple-negative breast cancer: 4-year follow-up data from BrighTNess, a randomized phase III trial. Ann Oncol. 2022;33(4):384–94. 10.1016/j.annonc.2022.01.009. 35093516 10.1016/j.annonc.2022.01.009

[55] Owonikoko TK, Dahlberg SE, Sica GL, Wagner LI, Wade JL 3rd, Srkalovic G, et al. Randomized phase II trial of cisplatin and etoposide in combination with veliparib or placebo for extensive-stage small-cell lung cancer: ECOG-ACRIN 2511 study. J Clin Oncol. 2019;37(3):222–9. 10.1200/jco.18.00264. 30523756 10.1200/JCO.18.00264 PMC6338394

[56] Jelinek MJ, Foster NR, Zoroufy AJ, Schwartz GK, Munster PN, Seiwert TY, et al. A phase I trial adding poly(ADP-ribose) polymerase inhibitor veliparib to induction carboplatin-paclitaxel in patients with head and neck squamous cell carcinoma: Alliance A091101. Oral Oncol. 2021;114: 105171. 10.1016/j.oraloncology.2020.105171. 33513474 10.1016/j.oraloncology.2020.105171 PMC7933088

[57] Ayoub JP, Wildiers H, Friedlander M, Arun BK, Han HS, Puhalla S, et al. Safety and efficacy of veliparib plus carboplatin/paclitaxel in patients with HER2-negative metastatic or locally advanced breast cancer: subgroup analyses by germline BRCA1/2 mutations and hormone receptor status from the phase-3 BROCADE3 trial. Ther Adv Med Oncol. 2021. 10.1177/17588359211059601. 34917174 10.1177/17588359211059601 PMC8669119

[58] Abraham JE, Pinilla K, Dayimu A, Grybowicz L, Demiris N, Harvey C, et al. The PARTNER trial of neoadjuvant olaparib in triple-negative breast cancer. Nature. 2024;629:1142–8. 10.1038/s41586-024-07384-2. 38588696 10.1038/s41586-024-07384-2 PMC11136660

[59] Drewett L, Lucey R, Pinilla KA, Grybowicz L, Wulff J, Dayimu A, et al. PARTNER: A randomized, phase II/III trial to evaluate the safety and efficacy of the addition of olaparib to platinum-based neoadjuvant chemotherapy in patients with triple-negative and/or germline BRCA-mutated breast cancer. J Clin Oncol. 2022;40(16_suppl):TPS619. 10.1200/JCO.2022.40.16_suppl.TPS619.

[60] Koczywas M, Frankel PH, Riess JW, El-Khoueiry AB, Villaruz LC, Leong S, et al. Phase I study of TRC102 in combination with cisplatin and pemetrexed in patients with advanced solid tumors/Phase II study of TRC102 with pemetrexed in patients with mesothelioma refractory to pemetrexed and cisplatin or carboplatin. J Clin Oncol. 2020;38(15_suppl):9055–9055. 10.1200/JCO.2020.38.15_suppl.9055.

[61] Son DJ, Hong JE, Ban JO, Park JH, Lee HL, Gu SM, et al. Synergistic inhibitory effects of cetuximab and cisplatin on human colon cancer cell growth via inhibition of the ERK-dependent EGF receptor signaling pathway. Biomed Res Int. 2015;2015: 397563. 10.1155/2015/397563. 26491668 10.1155/2015/397563 PMC4600871

[62] Trieu V, Pinto H, Riess JW, Lira R, Luciano R, Coty J, et al. Weekly docetaxel, cisplatin, and cetuximab in palliative treatment of patients with squamous cell carcinoma of the head and neck. Oncologist. 2018;23(7):764-e786. 10.1634/theoncologist.2017-0618. 29540603 10.1634/theoncologist.2017-0618 PMC6058339

[63] Guigay J, Aupérin A, Fayette J, Saada-Bouzid E, Lafond C, Taberna M, et al. Cetuximab, docetaxel, and cisplatin versus platinum, fluorouracil, and cetuximab as first-line treatment in patients with recurrent or metastatic head and neck squamous-cell carcinoma (GORTEC 2014–01 TPExtreme): a multicentre, open-label, randomised, phase 2 trial. Lancet Oncol. 2021;22(4):463–75. 10.1016/s1470-2045(20)30755-5. 33684370 10.1016/S1470-2045(20)30755-5

[64] Colombo N, Zaccarelli E, Baldoni A, Frezzini S, Scambia G, Palluzzi E, et al. Multicenter, randomised, open-label, non-comparative phase 2 trial on the efficacy and safety of the combination of bevacizumab and trabectedin with or without carboplatin in women with partially platinum-sensitive recurrent ovarian cancer. Br J Cancer. 2019;121(9):744–50. 10.1038/s41416-019-0584-5. 31537908 10.1038/s41416-019-0584-5 PMC6888836

[65] Moore KN, Miller A, Bell-McGuinn KM, Schilder RJ, Walker JL, O’Cearbhaill RE, et al. A phase I study of intravenous or intraperitoneal platinum based chemotherapy in combination with veliparib and bevacizumab in newly diagnosed ovarian, primary peritoneal and fallopian tube cancer. Gynecol Oncol. 2020;156(1):13–22. 10.1016/j.ygyno.2019.10.012. 31708167 10.1016/j.ygyno.2019.10.012 PMC7048389

[66] Thibault C, Fléchon A, Albiges L, Joly C, Barthelemy P, Gross-Goupil M, et al. Gemcitabine plus platinum-based chemotherapy in combination with bevacizumab for kidney metastatic collecting duct and medullary carcinomas: results of a prospective phase II trial (BEVABEL-GETUG/AFU24). Eur J Cancer. 2023;186:83–90. 10.1016/j.ejca.2023.03.018. 37054556 10.1016/j.ejca.2023.03.018

[67] Thomas S, Doebele RC, Spigel D, Tehfe M, Reck M, Verma S, et al. A phase 2 randomized open-label study of ramucirumab (RAM) plus first-line platinum-based chemotherapy in patients (pts) with recurrent or advanced non-small cell lung cancer (NSCLC): final results from squamous pts. Ann Oncol. 2017;28:ii42–3. 10.1093/annonc/mdx091.039.

[68] Yan M, Schwaederle M, Arguello D, Millis SZ, Gatalica Z, Kurzrock R. HER2 expression status in diverse cancers: review of results from 37,992 patients. Cancer Metastasis Rev. 2015;34(1):157–64. 10.1007/s10555-015-9552-6. 25712293 10.1007/s10555-015-9552-6 PMC4368842

[69] Mondaca S, Margolis M, Sanchez-Vega F, Jonsson P, Riches JC, Ku GY, et al. Phase II study of trastuzumab with modified docetaxel, cisplatin, and 5 fluorouracil in metastatic HER2-positive gastric cancer. Gastric Cancer. 2019;22(2):355–62. 10.1007/s10120-018-0861-7. 30088161 10.1007/s10120-018-0861-7 PMC6784321

[70] Piccart M, Procter M, Fumagalli D, Azambuja ED, Clark E, Ewer MS, et al. Adjuvant pertuzumab and trastuzumab in early HER2-positive breast cancer in the APHINITY trial: 6 years follow-up. J Clin Oncol. 2021;39(13):1448–57. 10.1200/jco.20.01204. 33539215 10.1200/JCO.20.01204

[71] Wagner AD, Grabsch HI, Mauer M, Romario UF, Kang Y-K, Bouche O, et al. Integration of trastuzumab (T), with or without pertuzumab (P), into perioperative chemotherapy (CT) of HER-2 positive gastric (GC) and esophagogastric junction cancer (EGJC): first results of the EORTC 1203 INNOVATION study, in collaboration with the Korean Cancer Study Group, and the Dutch Upper GI Cancer group. J Clin Oncol. 2023;41(16):4057–4057. 10.1200/JCO.2023.41.16_suppl.4057.37311151

[72] Fuentes-Antrás J, Genta S, Vijenthira A, Siu LL. Antibody-drug conjugates: in search of partners of choice. Trends in Cancer. 2023;9(4):339–54. 10.1016/j.trecan.2023.01.003. 36746689 10.1016/j.trecan.2023.01.003

[73] Moore KN, O’Malley DM, Vergote I, Martin LP, Gonzalez-Martin A, Malek K, et al. Safety and activity findings from a phase 1b escalation study of mirvetuximab soravtansine, a folate receptor alpha (FRα)-targeting antibody-drug conjugate (ADC), in combination with carboplatin in patients with platinum-sensitive ovarian cancer. Gynecol Oncol. 2018;151(1):46–52. 10.1016/j.ygyno.2018.07.017. 30093227 10.1016/j.ygyno.2018.07.017

[74] De Solar V, Contel M. Metal-based antibody drug conjugates. Potential and challenges in their application as targeted therapies in cancer. J Inorg Biochem. 2019;199:180. 10.1016/j.jinorgbio.2019.110780. 10.1016/j.jinorgbio.2019.110780 PMC6745269 31434020

[75] Huang R, Sun Y, Zhang XY, Sun BW, Wang QC, Zhu J. Biological evaluation of a novel Herceptin-platinum (II) conjugate for efficient and cancer cell specific delivery. Biomed Pharmacother. 2015;73:116–22. 10.1016/j.biopha.2015.05.013. 26211591 10.1016/j.biopha.2015.05.013

[76] Yin X, Zhuang Y, Song H, Xu Y, Zhang F, Cui J, et al. Antibody-platinum (IV) prodrugs conjugates for targeted treatment of cutaneous squamous cell carcinoma. J Pharm Anal. 2024;14(3):389–400. 10.1016/j.jpha.2023.11.002. 38618248 10.1016/j.jpha.2023.11.002 PMC11010626

[77] Ahn J, Miura Y, Yamada N, Chida T, Liu X, Kim A, et al. Antibody fragment-conjugated polymeric micelles incorporating platinum drugs for targeted therapy of pancreatic cancer. Biomaterials. 2015;39:23–30. 10.1016/j.biomaterials.2014.10.069. 25477168 10.1016/j.biomaterials.2014.10.069

[78] Zalba S, Contreras AM, Haeri A, Ten Hagen TL, Navarro I, Koning G, et al. Cetuximab-oxaliplatin-liposomes for epidermal growth factor receptor targeted chemotherapy of colorectal cancer. J Control Release. 2015;210:26–38. 10.1016/j.jconrel.2015.05.271. 25998052 10.1016/j.jconrel.2015.05.271

[79] Falvo E, Tremante E, Fraioli R, Leonetti C, Zamparelli C, Boffi A, et al. Antibody-drug conjugates: targeting melanoma with cisplatin encapsulated in protein-cage nanoparticles based on human ferritin. Nanoscale. 2013;5(24):12278–85. 10.1039/c3nr04268e. 24150593 10.1039/c3nr04268e

[80] Lin CY, Yang SJ, Peng CL, Shieh MJ. Panitumumab-conjugated and platinum-cored pH-sensitive apoferritin nanocages for colorectal cancer-targeted therapy. ACS Appl Mater Interfaces. 2018;10(7):6096–106. 10.1021/acsami.7b13431. 29368506 10.1021/acsami.7b13431

[81] Davis A, Tinker AV, Friedlander M. “Platinum resistant”; ovarian cancer: what is it, who to treat and how to measure benefit? Gynecol Oncol. 2014;133(3):624–31. 10.1016/j.ygyno.2014.02.038. 24607285 10.1016/j.ygyno.2014.02.038

[82] Laurent JS, Liu JF. Treatment approaches for platinum-resistant ovarian cancer. J Clin Oncol. 2024;42(2):127–33. 10.1200/jco.23.01771. 37910841 10.1200/JCO.23.01771

[83] Yusoh NA, Ahmad H, Gill MR. Combining PARP inhibition with platinum, ruthenium or gold complexes for cancer therapy. ChemMedChem. 2020;15(22):2121–35. 10.1002/cmdc.202000391. 32812709 10.1002/cmdc.202000391 PMC7754470

[84] Lee JM, Peer CJ, Yu M, Amable L, Gordon N, Annunziata CM, et al. Sequence-specific pharmacokinetic and pharmacodynamic phase I/Ib study of Olaparib tablets and carboplatin in women’s cancer. Clin Cancer Res. 2017;23(6):1397–406. 10.1158/1078-0432.Ccr-16-1546. 27663600 10.1158/1078-0432.CCR-16-1546 PMC5354956

[85] Coffman LG, Orellana TJ, Liu T, Frisbie LG, Normolle D, Griffith K, et al. Phase I trial of ribociclib with platinum chemotherapy in ovarian cancer. JCI Insight. 2022. 10.1172/jci.insight.160573. 35972817 10.1172/jci.insight.160573 PMC9675476

[86] Stronach EA, Alfraidi A, Rama N, Datler C, Studd JB, Agarwal R, et al. HDAC4-regulated STAT1 activation mediates platinum resistance in ovarian cancer. Cancer Res. 2011;71(13):4412–22. 10.1158/0008-5472.Can-10-4111. 21571862 10.1158/0008-5472.CAN-10-4111 PMC3130134

[87] Tjulandin S, Fedyanin M, Vladimirov VI, Kostorov V, Lisyanskaya AS, Krikunova L, et al. A multicenter phase II study of the efficacy and safety of quisinostat (an HDAC inhibitor) in combination with paclitaxel and carboplatin chemotherapy (CT) in patients (pts) with recurrent platinum resistant high grade serous epithelial ovarian, primarily peritoneal or fallopian tube carcinoma cancer (OC). J Clin Oncol. 2017;35(15):5541–5541. 10.1200/JCO.2017.35.15_suppl.5541.

[88] Meteran H, Knudsen A, Jørgensen TL, Nielsen D, Herrstedt J. Carboplatin plus paclitaxel in combination with the histone deacetylate inhibitor, vorinostat, in patients with recurrent platinum-sensitive ovarian cancer. J Clin Med. 2024;13(3):897. 10.3390/jcm13030897. 38337591 10.3390/jcm13030897 PMC10856581

[89] Dillon MT, Grove L, Newbold KL, Shaw H, Brown NF, Mendell J, et al. Patritumab with cetuximab plus platinum-containing therapy in recurrent or metastatic squamous cell carcinoma of the head and neck: an open-label, phase Ib study. Clin Cancer Res. 2019;25(2):487–95. 10.1158/1078-0432.Ccr-18-1539. 30327312 10.1158/1078-0432.CCR-18-1539

[90] Forster MD, Dillon MT, Kocsis J, Remenár E, Pajkos G, Rolland F, et al. Patritumab or placebo, with cetuximab plus platinum therapy in recurrent or metastatic squamous cell carcinoma of the head and neck: a randomised phase II study. Eur J Cancer. 2019;123:36–47. 10.1016/j.ejca.2019.08.017. 31648099 10.1016/j.ejca.2019.08.017

[91] Penson RT, Ambrosio AJ, Whalen CA, Krasner CN, Konstantinopoulos PA, Bradley C, et al. Phase II trials of iniparib (BSI-201) in combination with gemcitabine and carboplatin in patients with recurrent ovarian cancer. Oncologist. 2023;28(3):252–7. 10.1093/oncolo/oyac275. 36718018 10.1093/oncolo/oyac275 PMC10020803

[92] Mateo J, Ong M, Tan DSP, Gonzalez MA, de Bono JS. Appraising iniparib, the PARP inhibitor that never was—what must we learn? Nat Rev Clin Oncol. 2013;10(12):688–96. 10.1038/nrclinonc.2013.177. 24129347 10.1038/nrclinonc.2013.177

[93] Li J, Hu H, He J, Hu Y, Liu M, Cao B, et al. Effective sequential combined therapy with carboplatin and a CDC7 inhibitor in ovarian cancer. Transl Oncol. 2024;39: 101825. 10.1016/j.tranon.2023.101825. 37992591 10.1016/j.tranon.2023.101825 PMC10687335

[94] Waldman AD, Fritz JM, Lenardo MJ. A guide to cancer immunotherapy: from T cell basic science to clinical practice. Nat Rev Immunol. 2020;20(11):651–68. 10.1038/s41577-020-0306-5. 32433532 10.1038/s41577-020-0306-5 PMC7238960

[95] Sen S, Won M, Levine MS, Noh Y, Sedgwick AC, Kim JS, et al. Metal-based anticancer agents as immunogenic cell death inducers: the past, present, and future. Chem Soc Rev. 2022;51(4):1212–33. 10.1039/D1CS00417D. 35099487 10.1039/d1cs00417d PMC9398513

[96] Englinger B, Pirker C, Heffeter P, Terenzi A, Kowol CR, Keppler BK, et al. Metal drugs and the anticancer immune response. Chem Rev. 2019;119(2):1519–624. 10.1021/acs.chemrev.8b00396. 30489072 10.1021/acs.chemrev.8b00396

[97] Sun Q, Hong Z, Zhang C, Wang L, Han Z, Ma D. Immune checkpoint therapy for solid tumours: clinical dilemmas and future trends. Signal Transduct Target Ther. 2023;8(1):320. 10.1038/s41392-023-01522-4. 37635168 10.1038/s41392-023-01522-4 PMC10460796

[98] Hodi FS, O’Day SJ, McDermott DF, Weber RW, Sosman JA, Haanen JB, et al. Improved survival with ipilimumab in patients with metastatic melanoma. N Engl J Med. 2010;363(8):711–23. 10.1056/NEJMoa1003466. 20525992 10.1056/NEJMoa1003466 PMC3549297

[99] Twomey JD, Zhang B. Cancer immunotherapy update: FDA-approved checkpoint inhibitors and companion diagnostics. AAPS J. 2021;23(2):39. 10.1208/s12248-021-00574-0. 33677681 10.1208/s12248-021-00574-0 PMC7937597

[100] Garassino MC, Gadgeel S, Speranza G, Felip E, Esteban E, Dómine M, et al. Pembrolizumab plus pemetrexed and platinum in nonsquamous non-small-cell lung cancer: 5-Year outcomes from the phase 3 KEYNOTE-189 study. J Clin Oncol. 2023;41(11):1992–8. 10.1200/jco.22.01989. 36809080 10.1200/JCO.22.01989 PMC10082311

[101] Menis J, Bironzo P, Radj G, Greillier L, Monnet I, Livi L, et al. 9P Circulating tumour cells (CTCs) count and PD-L1 expression in untreated extensive small cell lung cancer patients treated in the REACTION trial, a phase II study of etoposide and cis/carboplatin with or without pembrolizumab (NCT02580994). Ann Oncol. 2020;31:S1420. 10.1016/j.annonc.2020.10.494.

[102] Janjigian YY, Maron SB, Chatila WK, Millang B, Chavan SS, Alterman C, et al. First-line pembrolizumab and trastuzumab in HER2-positive oesophageal, gastric, or gastro-oesophageal junction cancer: an open-label, single-arm, phase 2 trial. Lancet Oncol. 2020;21(6):821–31. 10.1016/s1470-2045(20)30169-8. 32437664 10.1016/S1470-2045(20)30169-8 PMC8229851

[103] Rudin CM, Awad MM, Navarro A, Gottfried M, Peters S, Csőszi T, et al. Pembrolizumab or placebo plus etoposide and platinum as first-Line therapy for extensive-stage small-cell lung cancer: randomized, double-blind, phase III KEYNOTE-604 study. J Clin Oncol. 2020;38(21):2369–79. 10.1200/jco.20.00793. 32468956 10.1200/JCO.20.00793 PMC7474472

[104] Horinouchi H, Nogami N, Saka H, Nishio M, Tokito T, Takahashi T, et al. Pembrolizumab plus pemetrexed-platinum for metastatic nonsquamous non-small-cell lung cancer: KEYNOTE-189 Japan Study. Cancer Sci. 2021;112(8):3255–65. 10.1111/cas.14980. 34036692 10.1111/cas.14980 PMC8353942

[105] Walsh CS, Kamrava M, Rogatko A, Kim S, Li A, Cass I, et al. Phase II trial of cisplatin, gemcitabine and pembrolizumab for platinum-resistant ovarian cancer. PLoS ONE. 2021;16(6): e0252665. 10.1371/journal.pone.0252665. 34081738 10.1371/journal.pone.0252665 PMC8174738

[106] Powles T, Csőszi T, Özgüroğlu M, Matsubara N, Géczi L, Cheng SY, et al. Pembrolizumab alone or combined with chemotherapy versus chemotherapy as first-line therapy for advanced urothelial carcinoma (KEYNOTE-361): a randomised, open-label, phase 3 trial. Lancet Oncol. 2021;22(7):931–45. 10.1016/s1470-2045(21)00152-2. 34051178 10.1016/S1470-2045(21)00152-2

[107] Liao JB, Gwin WR, Urban RR, Hitchcock-Bernhardt KM, Coveler AL, Higgins DM, et al. Pembrolizumab with low-dose carboplatin for recurrent platinum-resistant ovarian, fallopian tube, and primary peritoneal cancer: survival and immune correlates. J Immunother Cancer. 2021;9(9): e003122. 10.1136/jitc-2021-003122. 34531249 10.1136/jitc-2021-003122 PMC8449961

[108] Barber EL, Chen S, Pineda MJ, Robertson SE, Hill EK, Teoh D, et al. Clinical and biological activity of chemoimmunotherapy in advanced endometrial adenocarcinoma: a phase II trial of the Big Ten Cancer Research Consortium. Cancer Res Commun. 2022;2(10):1293–303. 10.1158/2767-9764.Crc-22-0147. 36388466 10.1158/2767-9764.CRC-22-0147 PMC9648489

[109] Chin AI, Ly A, Rodriguez S, Sachdeva A, Zomorodian N, Zhang H, et al. Updated results of a phase Ib single-center study of pembrolizumab in combination with chemotherapy in patients with locally advanced or metastatic small cell/neuroendocrine cancers of the prostate and urothelium. J Clin Oncol. 2023;41(6_suppl):165–165. 10.1200/JCO.2023.41.6_suppl.165.

[110] Kelley RK, Ueno M, Yoo C, Finn RS, Furuse J, Ren Z, et al. Pembrolizumab in combination with gemcitabine and cisplatin compared with gemcitabine and cisplatin alone for patients with advanced biliary tract cancer (KEYNOTE-966): a randomised, double-blind, placebo-controlled, phase 3 trial. Lancet. 2023;401(10391):1853–65. 10.1016/s0140-6736(23)00727-4. 37075781 10.1016/S0140-6736(23)00727-4

[111] Rha SY, Oh DY, Yañez P, Bai Y, Ryu MH, Lee J, et al. Pembrolizumab plus chemotherapy versus placebo plus chemotherapy for HER2-negative advanced gastric cancer (KEYNOTE-859): a multicentre, randomised, double-blind, phase 3 trial. Lancet Oncol. 2023;24(11):1181–95. 10.1016/s1470-2045(23)00515-6. 37875143 10.1016/S1470-2045(23)00515-6

[112] Isla D, Arriola E, Garcia Campelo MR, Diz Tain P, Marti Blanco C, Lopez-Brea Piqueras MM, et al. 1532P Phase IIIb study of durvalumab plus platinum-etoposide in first-line treatment of extensive-stage small cell lung cancer (CANTABRICO): preliminary efficacy results. Ann Oncol. 2022;33:S1247–8. 10.1016/j.annonc.2022.07.1627.

[113] Oh D-Y, He AR, Qin S, Chen L-T, Okusaka T, Vogel A, et al. Durvalumab plus gemcitabine and cisplatin in advanced biliary tract cancer. NEJM Evid. 2022;1(8):EVIDoa2200015. 10.1056/EVIDoa2200015. 38319896 10.1056/EVIDoa2200015

[114] Ready N, Hellmann MD, Awad MM, Otterson GA, Gutierrez M, Gainor JF, et al. First-line nivolumab plus ipilimumab in advanced non-small-cell lung cancer (CheckMate 568): outcomes by programmed death ligand 1 and tumor mutational burden as biomarkers. J Clin Oncol. 2019;37(12):992–1000. 10.1200/jco.18.01042. 30785829 10.1200/JCO.18.01042 PMC6494267

[115] Lee DH, Kim HR, Keam B, Kato K, Kuboki Y, Gao H, et al. Safety and tolerability of first-line durvalumab with tremelimumab and chemotherapy in esophageal squamous cell carcinoma. Cancer Med. 2023;12(15):16066–75. 10.1002/cam4.6260. 37489066 10.1002/cam4.6260 PMC10469840

[116] Xu N, Ying K, Wang Z, Liu Y, Jiang H, Zhou H, et al. Phase Ib study of sintilimab in combination with chemotherapy for 1L advanced or metastatic non-small cell lung cancer (NSCLC). J Clin Oncol. 2019;37(15):e20546. 10.1200/JCO.2019.37.15_suppl.e20546.

[117] Zhou C, Wu L, Fan Y, Wang Z, Liu L, Chen G, et al. Sintilimab plus platinum and gemcitabine as first-line treatment for advanced or metastatic squamous NSCLC: results from a randomized, double-blind, phase 3 trial (ORIENT-12). J Thorac Oncol. 2021;16(9):1501–11. 10.1016/j.jtho.2021.04.011. 34048947 10.1016/j.jtho.2021.04.011

[118] Zhang Y, Zeng L, Zhang X, Zhou Y, Zhang B, Jiang W, et al. P15.02 Toripalimab and platinum-doublet chemotherapy as neoadjuvant therapy for potentially resectable non-small cell lung cancer. J Thorac Oncol. 2021;16(10):S1014–5. 10.1016/j.jtho.2021.08.339.

[119] Clarke JM, Patel JD, Robert F, Kio EA, Thara E, Ross Camidge D, et al. Veliparib and nivolumab in combination with platinum doublet chemotherapy in patients with metastatic or advanced non-small cell lung cancer: a phase 1 dose escalation study. Lung Cancer. 2021;161:180–8. 10.1016/j.lungcan.2021.09.004. 34607210 10.1016/j.lungcan.2021.09.004

[120] Ahn J, Nagasaka M. Spotlight on cemiplimab-rwlc in the treatment of non-small cell lung cancer (NSCLC): focus on patient selection and considerations. Cancer Manag Res. 2023;15:627–34. 10.2147/cmar.S325856. 37457376 10.2147/CMAR.S325856 PMC10349595

[121] Lin D, Shen Y, Liang T. Oncolytic virotherapy: basic principles, recent advances and future directions. Signal Transduct Target Ther. 2023;8(1):156. 10.1038/s41392-023-01407-6. 37041165 10.1038/s41392-023-01407-6 PMC10090134

[122] Chakrabarty R, Tran H, Selvaggi G, Hagerman A, Thompson B, Coffey M. The oncolytic virus, pelareorep, as a novel anticancer agent: a review. Invest New Drugs. 2015;33(3):761–74. 10.1007/s10637-015-0216-8. 25693885 10.1007/s10637-015-0216-8

[123] Noonan AM, Farren MR, Geyer SM, Huang Y, Tahiri S, Ahn D, et al. Randomized phase 2 trial of the oncolytic virus pelareorep (Reolysin) in upfront treatment of metastatic pancreatic adenocarcinoma. Mol Ther. 2016;24(6):1150–8. 10.1038/mt.2016.66. 27039845 10.1038/mt.2016.66 PMC4923331

[124] Mahalingam D, Fountzilas C, Moseley J, Noronha N, Tran H, Chakrabarty R, et al. A phase II study of REOLYSIN(®) (pelareorep) in combination with carboplatin and paclitaxel for patients with advanced malignant melanoma. Cancer Chemother Pharmacol. 2017;79(4):697–703. 10.1007/s00280-017-3260-6. 28289863 10.1007/s00280-017-3260-6

[125] Jang D-I, Lee A-H, Shin H-Y, Song H-R, Park J-H, Kang T-B, et al. The role of tumor necrosis factor alpha (TNF-α) in autoimmune disease and current TNF-α inhibitors in therapeutics. Int J Mol Sci. 2021;22(5):2719. 33800290 10.3390/ijms22052719 PMC7962638

[126] Paik PK, Luo J, Ai N, Kim R, Ahn L, Biswas A, et al. Phase I trial of the TNF-α inhibitor certolizumab plus chemotherapy in stage IV lung adenocarcinomas. Nat Commun. 2022;13(1):6095. 10.1038/s41467-022-33719-6. 36241629 10.1038/s41467-022-33719-6 PMC9568581

[127] Liu J, Fu M, Wang M, Wan D, Wei Y, Wei X. Cancer vaccines as promising immuno-therapeutics: platforms and current progress. J Hematol Oncol. 2022;15(1):28. 10.1186/s13045-022-01247-x. 35303904 10.1186/s13045-022-01247-x PMC8931585

[128] Koeneman BJ, Schreibelt G, Gorris MAJ, Hins-de Bree S, Westdorp H, Ottevanger PB, et al. Dendritic cell vaccination combined with carboplatin/paclitaxel for metastatic endometrial cancer patients: results of a phase I/II trial. Front Immunol. 2024;15:1368103. 10.3389/fimmu.2024.1368103. 38444861 10.3389/fimmu.2024.1368103 PMC10912556

[129] Cibula D, Rob L, Mallmann P, Knapp P, Klat J, Chovanec J, et al. Dendritic cell-based immunotherapy (DCVAC/OvCa) combined with second-line chemotherapy in platinum-sensitive ovarian cancer (SOV02): a randomized, open-label, phase 2 trial. Gynecol Oncol. 2021;162(3):652–60. 10.1016/j.ygyno.2021.07.003. 34294416 10.1016/j.ygyno.2021.07.003

[130] Holloway RW, Thaker P, Mendivil AA, Ahmad S, Al-Niaimi AN, Barter J, et al. A phase III, multicenter, randomized study of olvimulogene nanivacirepvec followed by platinum-doublet chemotherapy and bevacizumab compared with platinum-doublet chemotherapy and bevacizumab in women with platinum-resistant/refractory ovarian cancer. Int J Gynecol Cancer. 2023;33(9):1458–63. 10.1136/ijgc-2023-004812. 37666539 10.1136/ijgc-2023-004812

[131] Pignata S, Scambia G, Ferrandina G, Savarese A, Sorio R, Breda E, et al. Carboplatin plus paclitaxel versus carboplatin plus pegylated liposomal doxorubicin as first-line treatment for patients with ovarian cancer: the MITO-2 randomized phase III trial. J Clin Oncol. 2011;29(27):3628–35. 10.1200/jco.2010.33.8566. 21844495 10.1200/JCO.2010.33.8566

[132] Vermorken JB, Mesia R, Rivera F, Remenar E, Kawecki A, Rottey S, et al. Platinum-based chemotherapy plus cetuximab in head and neck cancer. N Engl J Med. 2008;359(11):1116–27. 10.1056/NEJMoa0802656. 18784101 10.1056/NEJMoa0802656

[133] Wirth LJ, Dakhil SR, Kornek G, Axelrod R, Adkins D, Pant S, et al. PARTNER: A randomized phase II study of docetaxel/cisplatin (doc/cis) chemotherapy with or without panitumumab (pmab) as first-line treatment (tx) for recurrent or metastatic squamous cell carcinoma of the head and neck (R/M SCCHN). J Clin Oncol. 2013;31(15_suppl):6029–6029. 10.1200/jco.2013.31.15_suppl.6029. 10.1016/j.oraloncology.2016.07.005 27688102

[134] Cervantes-Ruiperez A, Hoskins P, Vergote I, Eisenhauer EA, Ghatage P, Carey M, et al. Final results of OV16, a phase III randomized study of sequential cisplatin-topotecan and carboplatin-paclitaxel (CP) versus CP in first-line chemotherapy for advanced epithelial ovarian cancer (EOC): A GCIG study of NCIC CTG, EORTC-GCG, and GEICO. J Clin Oncol. 2013;31(15_suppl):5502–5502. 10.1200/jco.2013.31.15_suppl.5502.

[135] Bröckelmann PJ, Müller H, Casasnovas O, Hutchings M, von Tresckow B, Jürgens M, et al. Risk factors and a prognostic score for survival after autologous stem-cell transplantation for relapsed or refractory Hodgkin lymphoma. Ann Oncol. 2017;28(6):1352–8. 10.1093/annonc/mdx072. 28327958 10.1093/annonc/mdx072

[136] Josting A, Müller H, Borchmann P, Baars JW, Metzner B, Döhner H, et al. Dose intensity of chemotherapy in patients with relapsed Hodgkin’s lymphoma. J Clin Oncol. 2010;28(34):5074–80. 10.1200/jco.2010.30.5771. 20975066 10.1200/JCO.2010.30.5771

[137] Daugaard G, Skoneczna I, Aass N, De Wit R, De Santis M, Dumez H, et al. A randomized phase III study comparing standard dose BEP with sequential high-dose cisplatin, etoposide, and ifosfamide (VIP) plus stem-cell support in males with poor-prognosis germ-cell cancer. An intergroup study of EORTC, GTCSG, and Grupo Germinal (EORTC 30974). Ann Oncol. 2011;22(5):1054–61. 10.1093/annonc/mdq575. 21059637 10.1093/annonc/mdq575 PMC3082158

[138] Tan YY, Al-Bubseeree B, Irvine D, MacDonald G, McQuaker G, Parker A, et al. High-dose chemotherapy in relapsed or refractory metastatic germ-cell cancer: the Scotland experience. Clin Genitourin Cancer. 2019;17(2):125–31. 10.1016/j.clgc.2018.11.013. 30563754 10.1016/j.clgc.2018.11.013

[139] Pelaz B, Alexiou C, Alvarez-Puebla RA, Alves F, Andrews AM, Ashraf S, et al. Diverse applications of nanomedicine. ACS Nano. 2017;11(3):2313–81. 10.1021/acsnano.6b06040. 28290206 10.1021/acsnano.6b06040 PMC5371978

[140] Duan X, He C, Kron SJ, Lin W. Nanoparticle formulations of cisplatin for cancer therapy. Wiley Interdiscip Rev Nanomed Nanobiotechnol. 2016;8(5):776–91. 10.1002/wnan.1390. 26848041 10.1002/wnan.1390 PMC4975677

[141] Cheng Q, Liu Y. Multifunctional platinum-based nanoparticles for biomedical applications. Wiley Interdiscip Rev Nanomed Nanobiotechnol. 2017;9(2): e1410. 10.1002/wnan.1410. 10.1002/wnan.1410 27094725

[142] Stathopoulos GP, Boulikas T. Lipoplatin formulation review article. J Drug Deliv. 2012;2012: 581363. 10.1155/2012/581363. 21904682 10.1155/2012/581363 PMC3166721

[143] Plummer R, Wilson RH, Calvert H, Boddy AV, Griffin M, Sludden J, et al. A phase I clinical study of cisplatin-incorporated polymeric micelles (NC-6004) in patients with solid tumours. Br J Cancer. 2011;104(4):593–8. 10.1038/bjc.2011.6. 21285987 10.1038/bjc.2011.6 PMC3049602

[144] Doi T, Hamaguchi T, Shitara K, Iwasa S, Shimada Y, Harada M, et al. NC-6004 Phase I study in combination with gemcitabine for advanced solid tumors and population PK/PD analysis. Cancer Chemother Pharmacol. 2017;79(3):569–78. 10.1007/s00280-017-3254-4. 28224231 10.1007/s00280-017-3254-4 PMC5344954

[145] Subbiah V, Grilley-Olson JE, Combest AJ, Sharma N, Tran RH, Bobe I, et al. Phase Ib/II Trial of NC-6004 (nanoparticle cisplatin) plus gemcitabine in patients with advanced solid tumors. Clin Cancer Res. 2018;24(1):43–51. 10.1158/1078-0432.Ccr-17-1114. 29030354 10.1158/1078-0432.CCR-17-1114

[146] Volovat SR, Ciuleanu TE, Koralewski P, Olson JEG, Croitoru A, Koynov K, et al. A multicenter, single-arm, basket design, phase II study of NC-6004 plus gemcitabine in patients with advanced unresectable lung, biliary tract, or bladder cancer. Oncotarget. 2020;11(33):3105–17. 10.18632/oncotarget.27684. 32913555 10.18632/oncotarget.27684 PMC7443368

[147] Osada A, Mangel L, Fijuth J, Żurawski B, Ursulovic T, Nikolin B, et al. Phase IIa/IIb clinical trial of NC-6004 (Nanoparticle Cisplatin) plus pembrolizumab in patients with head and neck cancer (HNSCC) who have failed platinum or a platinum-containing regimen. Eur J Cancer. 2020;138:S35. 10.1016/S0959-8049(20)31164-3.

[148] Cai L, Xu G, Shi C, Guo D, Wang X, Luo J. Telodendrimer nanocarrier for co-delivery of paclitaxel and cisplatin: a synergistic combination nanotherapy for ovarian cancer treatment. Biomaterials. 2015;37:456–68. 10.1016/j.biomaterials.2014.10.044. 25453973 10.1016/j.biomaterials.2014.10.044 PMC4312198

[149] Li J, Li Z, Li M, Zhang H, Xie Z. Synergistic effect and drug-resistance relief of paclitaxel and cisplatin caused by co-delivery using polymeric micelles. J Appl Polym Sci. 2015;132(6):41440. 10.1002/app.41440.

[150] Yang J, Ju Z, Dong S. Cisplatin and paclitaxel co-delivered by folate-decorated lipid carriers for the treatment of head and neck cancer. Drug Deliv. 2016;24(1):792–9. 10.1080/10717544.2016.1236849. 28494629 10.1080/10717544.2016.1236849 PMC8241145

[151] Liu B, Han L, Liu J, Han S, Chen Z, Jiang L. Co-delivery of paclitaxel and TOS-cisplatin via TAT-targeted solid lipid nanoparticles with synergistic antitumor activity against cervical cancer. Int J Nanomed. 2017;12:955–68. 10.2147/ijn.S115136. 10.2147/IJN.S115136 PMC5293363 28203075

[152] Yu T, Li Y, Gu X, Li Q. Development of a hyaluronic acid-based nanocarrier incorporating doxorubicin and cisplatin as a pH-sensitive and CD44-targeted anti-breast cancer drug delivery system. Front Pharmacol. 2020;11: 532457. 10.3389/fphar.2020.532457. 32982750 10.3389/fphar.2020.532457 PMC7485461

[153] Li H, Yu H, Zhu C, Hu J, Du M, Zhang F, et al. Cisplatin and doxorubicin dual-loaded mesoporous silica nanoparticles for controlled drug delivery. RSC Adv. 2016;6(96):94160–9. 10.1039/C6RA17213J.

[154] Kenny RG, Marmion CJ. Toward multi-targeted platinum and ruthenium drugs—A new paradigm in cancer drug treatment regimens? Chem Rev. 2019;119(2):1058–137. 10.1021/acs.chemrev.8b00271. 30640441 10.1021/acs.chemrev.8b00271

[155] Fronik P, Poetsch I, Kastner A, Mendrina T, Hager S, Hohenwallner K, et al. Structure-activity relationships of triple-action platinum(IV) prodrugs with albumin-binding properties and immunomodulating ligands. J Med Chem. 2021;64(16):12132–51. 10.1021/acs.jmedchem.1c00770. 34403254 10.1021/acs.jmedchem.1c00770 PMC8404199

[156] Griffith D, Morgan MP, Marmion CJ. A novel anti-cancer bifunctional platinum drug candidate with dual DNA binding and histone deacetylase inhibitory activity. Chem Commun. 2009;44:6735–7. 10.1039/b916715c. 10.1039/b916715c 19885462

[157] Parker JP, Nimir H, Griffith DM, Duff B, Chubb AJ, Brennan MP, et al. A novel platinum complex of the histone deacetylase inhibitor belinostat: rational design, development and in vitro cytotoxicity. J Inorg Biochem. 2013;124:70–7. 10.1016/j.jinorgbio.2013.03.011. 23603796 10.1016/j.jinorgbio.2013.03.011

[158] Griffith DM, Duff B, Suponitsky KY, Kavanagh K, Morgan MP, Egan D, et al. Novel trans-platinum complexes of the histone deacetylase inhibitor valproic acid; synthesis, in vitro cytotoxicity and mutagenicity. J Inorg Biochem. 2011;105(6):793–9. 10.1016/j.jinorgbio.2011.03.001. 21497577 10.1016/j.jinorgbio.2011.03.001

[159] Liskova B, Zerzankova L, Novakova O, Kostrhunova H, Travnicek Z, Brabec V. Cellular response to antitumor cis-Dichlorido platinum(II) complexes of CDK inhibitor Bohemine and its analogues. Chem Res Toxicol. 2012;25(2):500–9. 10.1021/tx200525n. 22250642 10.1021/tx200525n

[160] Wong DYQ, Lim JH, Ang WH. Induction of targeted necrosis with HER2-targeted platinum(iv) anticancer prodrugs. Chem Sci. 2015;6(5):3051–6. 10.1039/C5SC00015G. 28706680 10.1039/c5sc00015g PMC5490001

[161] Gong J, Shi T, Liu J, Pei Z, Liu J, Ren X, et al. Dual-drug codelivery nanosystems: an emerging approach for overcoming cancer multidrug resistance. Biomed Pharmacother. 2023;161:114505. 10.1016/j.biopha.2023.114505. 36921532 10.1016/j.biopha.2023.114505

[162] Liao Q, Zhang Y, Chu Y, Ding Y, Liu Z, Zhao X, et al. Application of artificial intelligence in drug-target interactions prediction: a review. npj Biomed Innov. 2025;2(1):1. 10.1038/s44385-024-00003-9. 10.1038/s44385-024-00003-9 PMC13055101 42032010

