# Supplementary material for: Advances in platinum-based cancer therapy: overcoming platinum resistance through rational combinatorial strategies
Source: Med Oncol. 2025 Jun 16;42(7):262. doi: 10.1007/s12032-025-02812-3 (PMC12167724; doi:10.1007/s12032-025-02812-3)
Supplement: Supplementary file 1 — Supplementary file1 (DOCX 528 KB) [file 12032_2025_2812_MOESM1_ESM.docx]

**Advances in platinum-based cancer therapy: overcoming platinum resistance through rational combinatorial strategies**

Nur Aininie Yusoh^1^, Haslina Ahmad^2,3^, Katherine A. Vallis^4^, and Martin R. Gill^5^*

^1^Department of Radiology, Huaxi MR Research Center (HMRRC), Institution of Radiology and Medical Imaging, West China Hospital of Sichuan University, Sichuan University, Chengdu, Sichuan, China.

^2^Department of Chemistry, Faculty of Science, Universiti Putra Malaysia, 43400 UPM Serdang, Selangor, Malaysia

^3^UPM-MAKNA Cancer Research Laboratory, Institute of Bioscience, Universiti Putra Malaysia, 43400 UPM Serdang, Selangor, Malaysia

^4^Oxford Institute for Radiation Oncology, Department of Oncology, University of Oxford, Oxford, United Kingdom

^5^Department of Chemistry, Faculty of Science and Engineering, Swansea University, Swansea, UK

Email: [m.r.gill@swansea.ac.uk](mailto:m.r.gill@swansea.ac.uk)

**Supplementary Information**

Supplementary Table S1: List of clinical trials evaluating platinum combinations.

| **Combinations** |  |  |  | **Platinum-based therapy** | **Conditions** | **Phase** | **N** | **Study number** | **Outcomes** | **Refs** | **Year published** |
| --- | --- | --- | --- | --- | --- | --- | --- | --- | --- | --- | --- |
| **Cytotoxic chemotherapies** | **Mitotic inhibitors** | Taxanes | Paclitaxel | Cisplatin or carboplatin | Patients with relapsed ovarian cancer | III | 800 | NCT00002894 | 2-year OS 57%  mOS 29 mo  PFS 12 mo | [S1] | 2003 |
|  |  |  | Paclitaxel | Cisplatin or carboplatin | Patients with ovarian cancer | III | 798 | - | mPFS 17.2 to 19.1 mo  mOS 43.3 mo to 44. mo | [S2] | 2003 |
|  |  |  | Paclitaxel | Cisplatin or carboplatin | Patients with optimally resected stage III ovarian cancer | III | 792 | - | mPFS 19.4 to 20.7 mo  mOS 48.7 to 57.4 mo | [S3] | 2003 |
|  |  |  | Paclitaxel | Carboplatin | Elderly patients with advanced non-small cell lung cancer (NSCLC) | II | 25 | - | ORR 28%  mS time 12.3 mo  1-year SR 52% | [S4] | 2005 |
|  |  |  | Paclitaxel | Cisplatin | Patients with stage IVB, recurrent, or persistent cervical carcinoma | III | 513 | NCT00064077 | ORR 29.1% | [S5] | 2009 |
|  |  |  | Paclitaxel | Carboplatin | Patients with ovarian cancer | III | 820 | NCT00326456 | mPFS 16.8 mo  mOS 53.2 mo | [S6] | 2011 |
|  |  |  | Paclitaxel | Carboplatin | Patients with advanced epithelial ovarian cancer | III | 819 | NCT00028743 | mPFS 16.2 mo  mOS 44.8 mo | [S7-10] | 2013 |
|  |  |  | Paclitaxel | Carboplatin | Patients with partially platinum-sensitive ovarian cancer | III | 344 | NCT00538603 | mPFS 9.4 mo | [S11] | 2015 |
|  |  |  | Paclitaxel | Carboplatin | Patients with BRCA-WT TNBC | II/III | 559 | NCT03150576 | pCR 52%  OS 87.2%  36-mo EFS 79% | [S12] | 2024 |
|  |  |  | Paclitaxel injection concentrate for nano-dispersion (PICN) (SPARC1023) | Carboplatin | Patients with solid tumor in advanced stage | I | 57 | NCT01304303 | mPFS 8.2 mo | [S13] | 2021 |
|  |  |  | Docetaxel | Cisplatin | Patients with non-operable adrenocortical carcinoma | II | 19 | NCT00324012 | RR 21%  mPFS 3 mo  mS 12.5 mo  1-year PFS 21% | [S14] | 2013 |
|  |  |  | Docetaxel | Carboplatin | Patients with anaplastic prostate cancer | II | 120 | NCT00514540 | mOS 16 mo | [S15, 16] | 2013 |
|  |  |  | Docetaxel | Carboplatin | Patients with stage I-III triple-negative breast cancer (TNBC) | II | 100 | NCT02413320 | pCR 52% | [S17] | 2019 |
|  |  |  | Docetaxel | Cisplatin | Patients with locally advanced nasopharyngeal carcinoma | - | 171 | NCT02575547 | - | [S18, 19] | 2019 |
|  |  | Vinca alkaloids | Vinorelbine (NVB) or vindensine (VDS) | Cisplatin (C) | Patients with advanced NSCLC | III | 612 | - | ORR 30% for NVB-C; 19% for VDS-C | [S20] | 1994 |
|  |  |  | Vinorelbine | Cisplatin | Patients with advanced NSCLC | II | 126 | - | - | [S21] | 1995 |
|  |  |  | Vinorelbine | Cisplatin | Patients with stage IVB, recurrent, or persistent cervical carcinoma | III | 513 | NCT00064077 | ORR 25.9% | [S5] | 2009 |
|  |  |  | Vinorelbine | Cisplatin | Patients with non-squamous NSCLC | II | 153 | EudraCT number 2009-012001-19 | DRR 75%  RR 24%  mPFS 4.2 mo  mS 10.2 mo | [S22] | 2014 |
|  |  |  | Vinorelbine | Cisplatin | Patients with advanced NSCLC with interstitial pneumonia | - | 67 | Institutional Review Board of National Cancer Center Hospital East (approval number: 2014-212) | ORR 34.3% mPFS 3.7 mo  mOS 7.4 mo  1-year SR 22.4% | [S23] | 2015 |
|  |  |  | Vinorelbine | Cisplatin | Patients with advanced squamous NSCLC | II | 114 | EudraCT number 2012-003531-40 | mPFS 4.2 mo  OS 10.2 mo | [S24] | 2021 |
|  | **Antimetabolites** | Nucleoside analagous | Gemcitabine | Cisplatin | Patients with locally advanced or metastatic NSCLC | III | 522 | - | RR 30.4%  OS 9.1 mo | [S25] | 2000 |
|  |  |  | Gemcitabine | Cisplatin | Patients with advanced NSCLC | II | 44 | - | ORR 73%  mPFS 10.6 mo mOS 15.0 mo | [S26] | 2002 |
|  |  |  | Gemcitabine | Cisplatin | Chemotherapy-naive patients with advanced-stage NSCLC | III | 863 | - | OS 10.3 mo | [S27] | 2008 |
|  |  |  | Gemcitabine | Cisplatin | Patients with stage IVB, recurrent, or persistent cervical carcinoma | III | 513 | NCT00064077 | ORR 22.3% | [S5] | 2009 |
|  |  |  | Gemcitabine | Cisplatin | Patients with advanced NSCLC | II | 28 | - | RR 33.3%  mOS 13 mo  1-year SR 55.6% | [S28] | 2010 |
|  |  |  | Gemcitabine | Cisplatin | Patients with biliary tract cancer | III | 410 | NCT00262769 | mOS 11.7 mo  mPFS 8.0 mo | [S29] | 2010 |
|  |  |  | Gemcitabine | Carboplatin | Patients with advanced urothelial cancer | II/III | 238 | NCT00014274 | ORR 41.2%  mOS 9.3 mo | [S30] | 2012 |
|  |  |  | Gemcitabine | Carboplatin | Patients with stage IV urinary tract cancer | III | 626 | NCT00022191 | mOS 12.7 mo  ORR 43.6% | [S31] | 2012 |
|  |  |  | Gemcitabine | Cisplatin + paclitaxel | Patients with stage IV urinary tract cancer | III | 626 | NCT00022191 | mOS 15.8 mo  ORR 55.5% | [S31] | 2012 |
|  |  |  | Gemcitabine | Cisplatin or carboplatin | Patients with advanced or metastatic urothelial carcinoma overexpressing HER2 | II | 563 | NCT01828736 | mPFS 10.2 mo  ORR 65.5%  mOS 15.7 mo | [S32] | 2015 |
|  |  |  | Gemcitabine | Cisplatin + nab-paclitaxel | Patients with advanced biliary tract cancer | II | 60 | NCT02392637 | mPFS 11.8 mo  DCR 84%  mOS 19.2 mo | [S33] | 2019 |
|  |  |  | Gemcitabine | Cisplatin | Patients with advanced biliary tract cancer | III | 246 | NCT02182778 | mOS 12.6 mo  1-year OS 53.7%  mPFS 5.5 mo  RR 15% | [S34] | 2023 |
|  |  |  | Gemcitabine | Cisplatin + S-1 (oral fluoropyrimidine) | Patients with advanced biliary tract cancer | III | 246 | NCT02182778 | mOS 13.5 mo  1-year OS 59.4%  mPFS 7.4 mo  RR 41.5% | [S34] | 2023 |
|  |  |  | Acelarin (a phosphoramidate transformation of gemcitabine) | Cisplatin | Patients with locally advanced/metastatic biliary tract cancers | Ib | 21 | NCT02351765 | ORR 33%  mPFS 7.2 mo  mOS 9.6 mo | [S35] | 2021 |
|  |  |  | Decitabine | Carboplatin | Patients with platinum resistant recurrent ovarian cancer | I/II | 28 | NCT00477386 | ORR 35%  mPFS 10 mo | [S36] | 2009 |
|  |  |  | Methotrexate | Carboplatin + vinblastine | Patients with advanced urothelial cancer | II/III | 238 | NCT00014274 | ORR 30.3%  mOS 8.1 mo | [S30] | 2012 |
|  |  |  | Azacitidine | Oxaliplatin | Patients with advanced cancers relapsed or refractory to any platinum therapy | I | 41 | NCT01039155 | mPFS 2.5 mo | [S37] | 2015 |
|  |  |  | Guadecitabine (a prodrug of decitabine) | Carboplatin | Patients with platinum-resistant, recurrent ovarian cancer | II | 120 | NCT01696032 | mPFS 4.0 mo  6-month PFS 37% | [S38] | 2020 |
|  |  |  | 5-Fluorouracil | Oxaliplatin | Patients with platinum-resistant recurrent ovarian carcinoma with a platinum-free interval of less than 6 months after any previous platinum-containing line of therapy | II | 90 | NCT01481701 | ORR 46%  mDR 7.0 mo | [S39] | 2014 |
|  |  |  | 5-Fluorouracil | Cisplatin + docetaxel | Patients with metastatic gastric adenocarcinoma | II | 85 | NCT00515411 | 6-month PFS 53%  mOS 12.6 mo | [S40] | 2015 |
|  |  |  | 5-Fluorouracil | Cisplatin + modified docetaxel | Patients with metastatic gastric adenocarcinoma | II | 85 | NCT00515411 | 6-month PFS 63%  mOS 18.8 mo | [S40] | 2015 |
|  |  |  | Capecitabine (a prodrug of 5-Fluorouracil) | Cisplatin + gemcitabine + docetaxel + | Patients with metastatic pancreatic cancer | II | 44 | NCT01459614 | RR 57%  DCR 87%  mOS 11.02 mo  mPFS 8.34 mo | [S41, 42] | 2014 |
|  |  |  | Capecitabine | Oxaliplatin | Patients with stage III colorectal cancer | III | 1886 | NCT00069121 | 7-year OS 73%  7-year DFS 63% | [S43] | 2015 |
|  |  |  | Capecitabine | Cisplatin + docetaxel | Patients with advanced gastric cancer | II | 46 | NCT00976976 | mRFS 26.9 mo  mOS 443.9 mo  5-year RFS 39.1%  5-year OS 41.3% | [S44] | 2015 |
|  |  | Antifolates | Leucovorin | Oxaliplatin + fluorouracil | Patients with advanced colorectal cancer | - | 1123 | - | 3-year DFS 78.2 % | [S45] | 2004 |
|  |  |  | Leucovorin | Oxaliplatin + fluorouracil | Patients with advanced colorectal cancer | II | 295 | NCT00039208 | mOS 17.4 mo | [S46] | 2005 |
|  |  |  | Pemetrexed | Cisplatin | Patients with MPM | II | 456 | - | mS 12.1 mo ORR 41.3% | [S47] | 2003 |
|  |  |  | Raltitrexed | Cisplatin | Patients with MPM | III | 250 | NCT00004920 | RR 23.6%  mOS 11.4 mo  1-year survival 46% | [S48] | 2005 |
|  |  |  | Pemetrexed | Carboplatin | Patients with malignant pleural mesothelioma (MPM) | II | 102 | - | ORR 18.6%  DC 65.7%  mOS 12.7 mo | [S49] | 2006 |
|  |  |  | Pemetrexed | Cisplatin | Patients with MPM | - | 728 | - | ORR 20.5%  CR 2%  PR 18.5%  mS 10.8 mo  1-year S 45.4% | [S50] | 2007 |
|  |  |  | Pemetrexed | Carboplatin | Patients with platinum-sensitive recurrent epithelial ovarian cancer | II | 22 | NCT01001910 | ORR 51.1%  mPFS 7.57 mo  mOS 20.3 mo | [S51] | 2008 |
|  |  |  | Pemetrexed | Carboplatin | Patients with MPM | II | 76 | - | CR 4%  PR 21%  ORR 25%  mS 14 mo | [S52] | 2008 |
|  |  |  | Pemetrexed | Cisplatin | Chemotherapy-naive patients with advanced-stage NSCLC | III | 862 | - | OS 10.3 mo | [S27] | 2008 |
|  |  |  | Pemetrexed | Cisplatin or carboplatin | Patients with locally advanced or metastatic NSCLC | II | 133 | NCT00402051 | 6-month mPFS 50.5%  mOS 11.7 mo  1-year SR 47.5% | [S53] | 2013 |
|  |  |  | Pemetrexed | Cisplatin | Patients with non-squamous NSCLC | II | 153 | EudraCT number 2009-012001-19 | DRR 76.5%  RR 31.4%  mPFS 4.3 mo  mS 10.8 mo | [S22] | 2014 |
|  |  |  | OSI-7904L (novel compound) | Oxaliplatin | Patients with advanced colorectal cancer | I | 14 | NCT00081237 | OSI-7904L 9 mg/m^2^  Oxaliplatin 130 mg/m^2^ | [S54] | 2008 |
|  | **Topoisomerase inhibitors** | Topo I inhibitor | Irinotecan | Cisplatin | Patients with locally advanced or metastatic penile cancer | II | 28 | NCT00066391 | RR < 30% | [S55] | 2008 |
|  |  |  | Irinotecan | Cisplatin, carboplatin or nedaplatin | Patients with inoperable and recurrent solid tumors | - | 331 | NCT01040312 | RR 52% | [S56] | 2016 |
|  |  |  | Irinotecan | Cisplatin + gemcitabine + docetaxel + capecitabine + | Patients with metastatic pancreatic cancer | I/II | 47 | NCT02324543 | RR 57%, mPFS 8.3 mo mOS 11 mo 36-month OS 19% | [S57] [S42] | 2022 |
|  |  |  | Topotecan | Cisplatin | Patients with stage IVB, recurrent, or persistent cervical carcinoma | III | 513 | NCT00064077 | ORR 23.4% | [S5] | 2009 |
|  |  |  | Topotecan | Cisplatin + topotecan; carboplatin + gemcitabine | Patients with advanced epithelial ovarian cancer | III | 819 | NCT00028743 | mPFS 14.6 mo  mOS 44.2 mo | [S7-10] | 2013 |
|  |  | Topo II inhibitors | Amrubicin | Cisplatin | Patients with extensive-stage small-cell lung cancer (SCLC) | II | 33 | NCT00388960 | ORR 77% | [S58] | 2011 |
|  |  |  | Amrubicin | Carboplatin | Patients with extensive-stage SCLC | II | 80 | NCT01076504 | ORR 74%  1-year SSR 38%  mS 10 mo | [S59] | 2018 |
|  |  |  | Etoposide | Cisplatin | Patients with extensive-stage SCLC | II | 33 | NCT00388960 | ORR 63% | [S58] | 2011 |
|  | **Antitumor antibiotics** |  | Bleomycin | Cisplatin + paclitaxel + etoposide | Men with germ cell cancer | II/III | 169 | NCT00003643 | 3-year PFS 79.4% | [S60] | 2012 |
|  |  |  | Bleomycin | Cisplatin + etoposide | Men with germ cell cancer | II/III | 168 | NCT00003643 | 3-year PFS 71.1% | [S60] | 2012 |
|  |  | Anthracycline | Doxorubicin | Cisplatin + gemcitabine + vinblastine + methotrexate | Patients who have undergone a radical cystectomy for stage III or Stage IV transitional cell carcinoma of the bladder urothelium | III | 284 | NCT00028756 | mPFS 2.9 yrs  5-year PFS 46.8%  mOS 6.8 yrs  5-year OS 53.6% | [S61] | 2014 |
|  |  |  | Doxorubicin | Carboplatin + cyclophosphamide + paclitaxel | Patients with stage I-III TNBC | II | 100 | NCT02413320 | pCR 55% | [S17] | 2019 |
|  |  |  | Pegylated liposomal doxorubicin (PLD) | Carboplatin | Patients with ovarian cancer recurrent within six to twelve months after initial carboplatin and paclitaxel chemotherapy | II | 58 | NCT00780039 | ORR 46%  mOS 19.1 mo | [S62] | 2009 |
|  |  |  | PLD | Carboplatin | Patients with ovarian cancer | III | 820 | NCT00326456 | mPFS 19.0 mo  mOS 61.6 mo | [S6] | 2011 |
|  |  |  | PLD | Carboplatin | Patients with partially platinum-sensitive ovarian cancer | III | 344 | NCT00538603 | mPFS 11.3 mo | [S11] | 2015 |
| **Stem cell therapies** |  |  | Stem cell transplant | Cisplatin + cytotoxics | Patients with relapsed hodgkin's lymphoma | III | 241 | NCT00025636 | - | [S63, 64] | 2010 |
|  |  |  | Stem cell transplant | Cisplatin + cytotoxics | Men with previously untreated germ cell cancer | III | 131 | NCT00003941 | 2-year FFS 44.8% | [S65] | 2011 |
|  |  |  | Stem cell transplant | Cisplatin + cytotoxics | Patients with relapsed or refractory germ cell tumors | III | 18 | NCT02375204 | 2-year PFS 67%  2-year OS 72% | [S66] | 2019 |
| **Targeted therapies**  **(small molecule inhibitors)** | **Selective small molecule kinase inhibitors** | EGFR inhibitors | Gefitinib | Cisplatin + gemcitabine | Patients with advanced NSCLC | III | 1037 | - | mS 9.9 mo  RR 50.3 | [S67] | 2004 |
|  |  |  | Gefitinib | Carboplatin + paclitaxel | patients with stage IIIB or IV NSCLC | pilot | 24 | - | PR 5/24  mS 8 mo | [S68] | 2003 |
|  |  |  | Gefitinib | Carboplatin + paclitaxel | Patients with advanced NSCLC | III | 1037 | - | mOS 9.8 mo | [S67] | 2004 |
|  |  |  | Gefitinib | Cisplatin + gemcitabine | Patients who are undergoing surgery for stage III NSCLC | II | - | NCT00103051 | - | - |  |
|  |  |  | Erlotinib | Carboplatin + paclitaxel | Patients with advanced NSCLC | III | 1059 | - | mS 10.6 mo | [S69] | 2005 |
|  |  |  | Erlotinib | Cisplatin + gemcitabine | Patients with advanced NSCLC | III | 1172 | - | Mos 10.8 mo  1-year SR 41% | [S70] | 2007 |
|  |  |  | Erlotinib | Platinum + gemcitabine | Patients with advanced NSCLC | II | 154 | NCT01998919 | mPFS 7.2 mo | [S71] | 2008 |
|  |  |  | Erlotinib | Platinum + gemcitabine | Patients with NSCLC with EGFR mutation-positive disease or unknown EGFR mutation status | III | 451 | NCT00883779 | mPFS 7.6 mo  mOS 18.3 mo | [S72] | 2013 |
|  |  |  | Erlotinib | Platinum + docetaxel | Patients with metastatic or recurrent head and neck squamous cell carcinoma (HNSCC) | II | 55 | NCT01064479 | RR 56%  mPFS 6.1 mo  mOS 17.0 mo | [S73] | 2017 |
|  |  | VEGFR inhibitors | Axitinib | Platinum + paclitaxel or gemcitabine | Patients with advanced NSCLC and other solid tumors | I | 49 | NCT00454649 | ORR 37% for APC and 23.8% for AGC | [S74] | 2012 |
|  |  | ALK inhibitors | Ceritinib | Cisplatin + gemcitabine | Patients with advanced solid tumors | I | 38 | NCT02227940 | ORR 20%  mPFS 4.8 mo | [S75] | 2021 |
|  |  | WEE1 inhibitors | Adavosertib | Carboplatin | Patients with TP53-mutated ovarian cancer refractory or resistant to first-line therapy within 3 months | II | 24 | NCT01164995 | ORR 43%  mPFS 5.3 mo  mOS 12.6 mo | [S76] | 2016 |
|  |  |  | Adavosertib (A) | Cisplatin + docetaxel | Patients with borderline resectable HNSCC | I | 12 | NCT02508246 | MTD A 150 mg orally bid for 2.5 days | [S77] | 2017 |
|  |  |  | Adavosertib | Carboplatin + paclitaxel | Patients with platinum-sensitive TP53-mutant ovarian cancer | II | 121 | NCT01357161 | mPFS 7.9 mo | [S78] | 2020 |
|  |  | PI3K/mTOR inhibitors | Gedatolisib | Cisplatin | Patients with TNBC | I | 110 | NCT01920061 | ORR 40% | [S79, 80] | 2016 |
|  |  | CDK inhibitors | Palbociclib | Carboplatin | Patients with unresectable recurrent or metastatic HNSCC | II | 21 | NCT03194373 | 12-week DCR 33%  mPFS 2.9 mo  mOS 4.6 mo | [S81] | 2020 |
|  |  |  | Ribociclib | Carboplatin + paclitaxel | Patients with recurrent platinum-sensitive ovarian cancer | I | 35 | NCT03056833 | ORR 79.3%  SDR 18%  mPFS 11.4 mo | [S82] | 2022 |
|  | **Multi-kinase small molecule inhibitors** |  | Sorafenib | Cisplatin + docetaxel | Patients with metastatic or advanced gastric or gastroesophageal junction cancer | II | 44 | NCT00253370 | PR 41%  mPFS 5.8 mo  mOS 13.6 mo | [S83] | 2010 |
|  |  |  | Nintedanib (N) | Carboplatin + paclitaxel (CP) | Patients with advanced gynecological malignancies | I | 22 | - | MTD of N 200 mg bid + CP  PFS 177 to 282 days | [S84] | 2010 |
|  |  |  | Nintedanib | Carboplatin + PLD | Patients with recurrent ovarian cancer | I | 13 | NCT01314105 | MTD of N 200 mg bid + carboplatin/PLD (AUC 5/30 mg/m2) | [S85] | 2014 |
|  |  |  | Pazopanib | Carboplatin + paclitaxel | Patients with advanced gynaecologic tumours | I/II | 6 | NCT00561795) | DLTs (2/6)  Severe myelotoxicity | [S86] | 2012 |
|  |  |  | Sunitinib | Cisplatin or carboplatin | Patients with untreated extensive-stage SCLC | II | 144 | NCT00453154 | mPFS 3.7 mo  mOS 9.0 mo | [S87] | 2015 |
|  |  |  | Lapatinib | Cisplatin + cytotoxics | Patients with stomach cancer or gastroesophageal junction cancer | II | 29 | NCT01123473 | mPFS 8.0 mo  mOS 13.8 mo | [S88] | 2018 |
|  | **Selective small molecule non-kinase inhibitors** | HDAC inhibitors | Vorinostat | Cisplatin + gemcitabine | Patients with advanced NSCLC | I | 28 | - | DLTs (2/28)  PR 47% (9/19)  SD (8/19)  DS (2/19) | [S89] | 2009 |
|  |  |  | Vorinostat | Carboplatin + paclitaxel | Patients with advanced NSCLC | II | 94 | NCT00481078 | RR 34%  mPFS 6.0 mo  OS 13 mo | [S90] | 2010 |
|  |  |  | Quisinostat | Carboplatin + paclitaxel | Patients with recurrent and platinum-resistant high grade serous epithelial ovarian, primarily peritoneal or fallopian tube carcinoma cancer | II | 31 | NCT02948075 | ORR 50%  mDoR 5 mo  mPFS 6 mo | [S91] | 2017 |
|  |  |  | Quisinostat | Carboplatin + paclitaxel | Patients with recurrent platinum-sensitive ovarian cancer | II | 55 | NCT00772798 | CR 26.4%  PR 35.8%  ORR 62.2%  mPFS 11.6 mo  mOS 40.6 mo | [S92] | 2024 |
|  |  | XIAP inhibitors | Phenoxodiol | Cisplatin | Patients with platinum/taxane-refractory/resistant epithelial ovarian, fallopian tube, or primary peritoneal cancers | II | 32 | - | SD 56%  ORR 19% | [S93] | 2011 |
|  |  | Proteasome inhibitor | Bortezomib | Cisplatin | Patients with MPM | II | 82 | NCT00458913 | 18-weeks PFS 53%  mPFS 5.1 mo  OS 13.5 mo  1-year S 56%  RR 28.4% | [S94] | 2013 |
|  |  | Vascular-disrupting agents | Ombrabulin | Platinum/docetaxel or platinum/paclitaxel | Patients with advanced solid tumor | I | 71 | NCT00719524 | mPR 5.5 mo | [S95] | 2014 |
|  |  | Proton pump inhibitors | Esomeprazole | Cisplatin + docetaxel | Patients with metastatic breast cancer | II | 94 | NCT01069081 | ORR 67.7%  OS 29.9 mo | [S96] | 2015 |
|  |  | PARP inhibitors | Rucaparib | Cisplatin | Patients with TNBC with BRCA1/2 mutations | II | 128 | NCT01074970 | 2-year DFS 63.1% | [S97] | 2015 |
|  |  |  | Olaparib | Carboplatin | Patients with refractory or recurrent women’s cancers | I | 77 | NCT01237067 | RR 68% for BRCA mutation carriers and 19% without BRCA mutation carriers | [S98] | 2017 |
|  |  |  | Veliparib | Carboplatin + gemcitabine | Patients with advanced ovarian cancer and other solid malignancies patients | I | 75 | NCT01063816 | MTD/RP2D veliparib 250 mg + carboplatin AUC 4 + gemcitabine 800mg/m^2^  PR 45%  CR 24% | [S99] | 2018 |
|  |  |  | Veliparib | Carboplatin + paclitaxel + cyclophosphamide + doxorubicin | Patients with untreated stage II-IIITNBC | III | 634 | NCT02032277 | pCR 53% | [S100, 101] | 2018 |
|  |  |  | Veliparib | Cisplatin + etoposide | Patients with extensive-stage SCLC | II | 128 | NCT01642251 | mOS 10.3 mo  ORR 71.9% | [S102] | 2019 |
|  |  |  | Veliparib | Platinum + paclitaxel | Patients with advanced HNSCC | I | 20 | NCT01711541 | RP2D veliparib + carboplatin + paclitaxel 350 mg bid  1-year OS 77.8%  1-year PFS 66.7% | [S103] | 2021 |
|  |  |  | Veliparib | Carboplatin + paclitaxel | Patients with HER2-negative metastatic or locally advanced breast cancer | III | 509 | NCT02163694 | mPFS 13.0 mo HR+; 16.6 mo TNBC; gBRCA1 14.2 mo  2-year PFS 27.5% HR+; 40.4% TNBC | [S104] | 2021 |
|  |  |  | Iniparib | Carboplatin + gemcitabine | Patients with recurrent ovarian cancer | II | 43 | NCT01033123  NCT01033292 | Platinum-sensitive; ORR 66%  mPFS 9.9 mo  platinum-resistant; ORR 26%  mPFS 6.8 mo | [S105] | 2023 |
|  |  |  | Olaparib | Carboplatin/paclitaxel | Patients with BRCA-WT TNBC | II/III | 559 | NCT03150576 | pCR 51%  OS 90%  36-mo EFS 80% | [S12] | 2024 |
|  |  | Arginine deprivation | Pegylated arginine deiminase (ADI-PEG20) | Cisplatin + pemetrexed | Patients with ASS1-deficient thoracic cancers | I | 9 | NCT02029690 | PR 78% | [S106] | 2017 |
|  |  |  | Pegylated arginine | Cisplatin + pemetrexed | Patients with ASS1-deficient non-epithelioid MPM | II/III | 249 | NCT02709512 | mOS 9.3 mo  mPFS 6.2 mo | [S107, 108] | 2017 |
|  |  |  | Pegylated arginine deiminase | Cisplatin + pemetrexed | Patients with ASS1-deficient recurrent high-grade glioma | I | 10 | NCT02029690 | SD 80%  mPFS 5.2 mo  OS 6.3 mo | [S109] | 2019 |
|  |  | Wnt inhibitors | Ipafricept | Carboplatin + paclitaxel | Patients with recurrent platinum-sensitive ovarian cancer | I | 37 | NCT02092363 | No DLTs  ORR 75.7%  mPFS 10.3 mo  OS 33 mo | [S110] | 2019 |
|  |  | Bcl-2 inhibitors | AT-101 | Platinum + docetaxel | Patients with advanced laryngeal cancer | II | 55 | NCT01633541 | 2-year OS 81% | [S111, 112] | 2019 |
|  |  | BER inhibitors | Methoxyamine | Cisplatin + pemetrexed | Patients with advanced solid tumors | I | 16 | NCT02535312 | No DLTs  mPFS 7.1% (1.4-15.5 mo) | [S113] | 2020 |
| **Targeted therapies**  **(monoclonal antibodies)** | **mAbs** | Anti-EGFR | Cetuximab | Carboplatin + paclitaxel | Untreated patients with stage IV NSCLC | I/II | 32 | - | OR 26%  mS 11 mo  1-year SR 40%  2-year SR 16% | [S114] | 2005 |
|  |  |  | Cetuximab | Carboplatin | Patients with EGFR-positive, relapsed platinum-sensitive ovarian or primary peritoneal carcinoma | II | 28 | NCT00086892 | ORR (9/26) | [S115] | 2008 |
|  |  |  | Cetuximab | Platinum + 5-fluorouracil | Patients with recurrent or metastatic head and neck cancer | III | 442 | NCT00122460 | mOS 10.1 mo  mPFS 5.6 mo | [S116] | 2008 |
|  |  |  | Cetuximab | Platinum + paclitaxel or platinum + gemcitabine or platinum + pemetrexed | Patients with recurrent or advanced NSCLC | IIb | 601 | NCT00828841 | mOS 9.3-10.6 mo NSQ; 7.1-9.6 mo SQ  1-year S 42.9-47.3% NSQ; 32.6-35.4% SQ | [S117] | 2012 |
|  |  |  | Cetuximab + cilengitide (integrin inhibitor) | Cisplatin + vilnorelbine or cisplatin + gemcitabine | Patients with advanced NSCLC | II | 169 | NCT00842712 | PFS 6.2 mo  mOS 13.6 mo | [S118] | 2015 |
|  |  |  | Cetuximab | Cisplatin + fluorouracil + docetaxel | Patients with stage III-IV resectable oropharynx cancer | II | 42 | NCT00665392 | ORR 58% | [S119] | 2015 |
|  |  |  | Cetuximab | Cisplatin + docetaxel | Patients with recurrent or metastatic HNSCC | II | 54 | NCT01289522 | ORR 44.4%  mOS 14 mo  mPFS 6.2 mo | [S120] | 2015 |
|  |  |  | Cetuximab | Platinum doublets | Patients with NSCLC | III | 583 | NCT00820755 | 1-year SR 64.4% | [S121] | 2015 |
|  |  |  | Cetuximab | Cisplatin + docetaxel | Patients with HNSCC | II | 29 | NCT01437449 | ORR 56%  mPFS 4.8 mo  mOS 14.7 mo | [S122] | 2018 |
|  |  |  | Cetuximab | Cisplatin + 5-fluorouracil (EXTREME) or cisplatin + docetaxel (TPEx) | patients with recurrent or metastatic HNSCC | II | 541 | NCT02268695 | mOS 14.5 mo TPEx; 13.4 mo EXTREME | [S123] | 2021 |
|  |  |  | Panitumumab | Cisplatin + docetaxel | Patients with head and neck cancer | II | 113 | NCT00454779 | mPFS 6.9 mo  mOS 12.9 mo  ORR 44% | [S124] | 2013 |
|  |  | Anti-VEGFR | Cediranib | Carboplatin + paclitaxel | Patients with advanced NSCLC | I | 20 | - | No dose-limiting toxicities  CR 9/20 | [S125] | 2008 |
|  |  |  | Bevacizumab | Platinum doublets | Patients with NSCLC and brain metastases | II | 115 | NCT00312728 | - | [S126] | 2009 |
|  |  |  | Bevacizumab | Platinum-based chemotherapy | Patients with advanced or recurrent non-squamous NSCLC | IV | 2212 | NCT00451906 | mOS 14.6 mo | [S127] | 2010 |
|  |  |  | Bevacizumab | Platinum-based chemotherapy | Asian patients with advanced non-squamous NSCLC | IV | 314 | NCT00451906 | DCR 94.1%  mOS 18.9 mo | [S128] | 2011 |
|  |  |  | Bevacizumab | Platinum-based chemotherapy | Elderly patients with advanced non-squamous NSCLC | IV | 623 | NCT00451906 | mOS 14.6 mo  RR 49.3%  DCR 89.3% | [S129] | 2011 |
|  |  |  | Bevacizumab | Cisplatin + docetaxel + fluorouracil | Patients with unresectable or metastatic gastroesophageal adenocarcinoma | II | 48 | NCT00390416 | 6-month PFS 79%  mPFS 12 mo  mOS 16. Mo  2-year SR 37% | [S130] | 2011 |
|  |  |  | Bevacizumab | Cisplatin + docetaxel + surgery | Patients with resectable NSCLC | II | 71 | NCT00130780 | - | [S131] | 2011 |
|  |  |  | Bevacizumab | Cisplatin + radiation therapy | Patients with previously untreated locally advanced cervical cancer | II | 60 | NCT00369122 | No treatment-related SAEs | [S132] | 2012 |
|  |  |  | Bevacizumab | Carboplatin + trabectedin | Patients with partially platinum-sensitive recurrent ovarian cancer | II | 71 | NCT01735071 | 6-month PFS 85% | [S133] | 2019 |
|  |  |  | Bevacizumab | Platinum + paclitaxel + veliparib | Patients with newly diagnosed ovarian, primary peritoneal and fallopian tube cancer | I | 424 | NCT00989651 | MTD 250 mg veliparib BID + feasible dose 150 mg BID | [S134] | 2020 |
|  |  |  | Bevacizumab | Platinum + gemcitabine | Patients with kidney metastatic collecting duct and medullary carcinomas | II | 36 | NCT02363751 | 6-month ORR 29.4%  6-month PFS 47.1%  mOS 11.1 mo | [S135] | 2023 |
|  |  |  | Ramucirumab | Platinum + pemetrexed | Patients with untreated recurrent or advanced NSCLC | II | 280 | NCT01160744 | ORR 49.3%  PFS 7.2 mo  DCR 85.5% | [S136, 137] | 2015 |
|  |  |  | Ramucirumab | Platinum-based chemotherapy | Japanese patients) with metastatic gastric/gastroesophageal junction adenocarcinoma | 1b | 18 | NCT02359058 | DCR 100%  ORR 45% | [S138] | 2017 |
|  |  | Anti-MET | Onartuzumab | Platinum + bevacizumab + paclitaxel or platinum + pemetrexed | Patients with stage IIIb or IV non-squamous NSCLC | II | 259 | NCT01496742 | - | [S139] | 2012 |
|  |  |  | Onartuzumab | Platinum + paclitaxel | Patients with previously untreated advanced squamous cell NSCLC | II | 109 | NCT01519804 | ORR 40%  mOS 9.1 mo | [S140] | 2017 |
|  |  | Anti-HER2 | Pertuzumab | Carboplatin + (paclitaxel or gemcitabine) | Patients with platinum-sensitive, recurrent advanced ovarian cancer | II | 149 | NCT02004093 | mPFS 34.1 weeks  mOS 28.2 mo | [S141] | 2013 |
|  |  |  | Trastuzumab | Cisplatin + S-1 (oral fluoropyrimidine) | Patients with HER-2-positibe gastric cancer | II | 56 | UMIN-CTR, UMIN000005739 | RR 68%  DCR 94%  mOS 16.0 mo  mPFS 7.8 mo | [S142] | 2014 |
|  |  |  | Trastuzumab | Platinum + gemcitabine | Patients with advanced or metastatic urothelial carcinoma overexpressing HER2 | II | 563 | NCT01828736 | mPFS 8.2 mo  ORR 53.2%  mOS 14.1 mo | [S32] | 2015 |
|  |  |  | Trastuzumab | Cisplatin + modified docetaxel + 5-fluorouracil | Patients with metastatic HER2-positive gastric cancer | II | 26 | NCT00515411 | 6-month PFS 73%  ORR 65%  mPFS 13 mo  mOS 24.9 mo | [S143] | 2019 |
|  |  |  | Pertuzumab + Trastuzumab | Carboplatin + cytotoxics | Patients with HER2-positive primary breast cancer | III | 4805 | NCT01358877 | 6-year OS 95% | [S144-146] | 2017 |
|  |  |  | Pertuzumab + Trastuzumab | Cisplatin + 5-FU or capecitabine + surgery | Patients gastric or gastroesophageal junction adenocarcinoma | II | 161 | NCT02205047 | mORR 26.4% | [S147, 148] | 2019 |
|  |  | Anti-IGF1R | Cixutumumab (C) or (vismodegib (V), a hedgehog inhibitor) | Cisplatin + etoposide | Patients With extensive stage or recurrent SCLC | II | 155 | NCT00887159 | mPFS 4.4 mo for C; 4.6 mo for v  mOS 9.8 for C; 10.1 mo for V  RR 52% for C; 49% for V | [S149] | 2013 |
|  |  | Anti-CD20 | Ofatumumab | Carboplatin + bendamustine + etoposide | Patients with refractory or relapsed aggressive B-cell non-Hodgkin lymphomas (NHL) | I/II | 11 | NCT01458366 | ORR 64%  12-month OS 63.5% | [S150] | 2014 |
|  |  | Anti-HGF and anti-IGF1R | Rilotumumab (R) or ganitumab (G) | Platinum + etoposide | Patients with extensive stage SCLC | II | 185 | NCT00791154 | mOS 12.2 for R; 10.7 for G  mPFS 5.4 for R; 5.5 for G | [S151] | 2017 |
|  |  | Anti-HER3 | Patritumab | Platinum + cetuximab | Patients with recurrent and/or metastatic HNSCC | I | 15 | NCT02350712 | No DLTs  mPFS 7.9 mo  mOS 13.5 mo | [S152] | 2019 |
|  |  |  | Patritumab | Platinum + cetuximab | Patients with recurrent and/or metastatic HNSCC | II | 87 | NCT02633800 | mPFS 5.6 mo  mOS 10.0 mo | [S153] | 2019 |
|  |  | Anti-FRα | Farletuzumab | Carboplatin + paclitaxel or carboplatin + PLD | Patients with low CA-125 platinum-sensitive ovarian cancer | II | 214 | NCT02289950 | mPFS 11.7 mo | [S154, 155] | 2021 |
|  |  | Anti-RANK | Denosumab | Platinum doublet | Patients with NSCLC | III | 514 | NCT02129699 | mOS 8.2 mo | [S156, 157] | 2020 |
| **Targeted therapies**  **(Antibody-drug conjugate (ADC))** | **ADC** | Anti-FRα | Mirvetuximab soravtansine linked to a cytotoxic drug called DM4 (a maytansinoid) | Carboplatin | Patients with platinum-sensitive ovarian cancer | Ib | 18 | NCT02606305 | ORR 71%  mPFS 15 mo | [S158] | 2018 |
| **Immunotherapies** | **Immune checkpoint inhibitors** | Anti-PD-1 | Camrelizumab | Cisplatin + gemcitabine | Patients with nasopharyngeal carcinoma | I | 23 | NCT03121716 | OR 91% | [S159] | 2018 |
|  |  |  | Sintilimab | Cisplatin + pemetrexed or cisplatin + gemcitabine | Patients with advanced or metastatic NSCLC | 1b | 41 | NCT02937116 | ORR 68.4% NSQ; 64.7% SQ  mPFS 11.4 mo NSQ; 6.5 mo SQ | [S160] | 2019 |
|  |  |  | Sintilimab | Platinum + gemcitabine | Patients with advanced or metastatic squamous NSCLC | III | 357 | NCT03629925 | mPFS 5.1 mo  6-month PFS 41.4% | [S161] | 2021 |
|  |  |  | Pembrolizumab | Cisplatin + pemetrexed | Patients with previously untreated metastatic nonsquamous NSCLC | III | 616 | NCT02578680 | 57 patients who completed 35 cycles; ORR 86% and 3-year OS 71.9%  5-year OS 19.4% | [S162-165] | 2020 |
|  |  |  | Pembrolizumab | Platinum  + etoposide | Patients with untreated extensive SCLC | II | 125 | NCT02580994 | RR 61%  mOS 12.3 mo  mPFS 4.7 mo | [S166] | 2020 |
|  |  |  | Pembrolizumab | Cisplatin + capecitabine + trastuzumab or oxaliplatin + 5-FU + trastuzumab | Patients with HER2-positive oesophageal, gastric, or gastro-oesophageal junction cancer | II | 37 | NCT02954536 | 6-month PF 70% | [S167] | 2020 |
|  |  |  | Pembrolizumab | Platinum + etoposide | Patients with extensive stage SCLC | III | 453 | NCT03066778 | 12-month PFS 13.6%  24-month OS 22.5%  ORR 70.6% | [S168] | 2020 |
|  |  |  | Pembrolizumab | Cisplatin + pemetrexed | Japanese patients with previously untreated metastatic nonsquamous NSCLC | III | 40 | NCT02578680 | mPFS 7.1 mo | [S169] | 2021 |
|  |  |  | Pembrolizumab | Cisplatin + gemcitabine | Patients with recurrent platinum-resistant ovarian cancer | II | 21 | NCT02608684 | ORR 60%  mPFS 6.2 mo  mOS 11.3 mo | [S170] | 2021 |
|  |  |  | Pembrolizumab | Platinum + gemcitabine | Patients with urothelial carcinoma | III | 1010 | NCT02853305 | mPFS 7.1 mo  mOS 17.0 mo | [S171] | 2021 |
|  |  |  | Pembrolizumab | Carboplatin | Patients with recurrent platinum-resistant ovarian, fallopian tube, and primary peritoneal cancer | I/II | 29 | NCT03029598 | PR 56.5%  SD 51.7%  mPFS 4.6 mo  mOS 11.3 mo | [S172] | 2021 |
|  |  |  | Pembrolizumab | Carboplatin + paclitaxel | Patients with advanced endometrial cancer | II | 46 | NCT02549209 | ORR 74.4%  mPFS 10.6 mo | [S173] | 2022 |
|  |  |  | Pembrolizumab | Platinum + etoposide | Patients with locally advanced or metastatic small cell/neuroendocrine cancers of urothelium or prostate | I | 1 | NCT03582475 | ORR 43%  12-month PFS rate 64% | [S174] | 2023 |
|  |  |  | Pembrolizumab | Platinum + pemetrexed | Patients with NSCLC | II | 117 | NCT03664024 | ORR 40.2%  mPFS 7.2 mo  mOS 18.1 mo | [S175] | 2023 |
|  |  |  | Pembrolizumab | Cisplatin + gemcitabine | Patients with advanced or metastatic biliary tract cancer | III | 1069 | NCT04003636 | mOS 12.7 mo | [S176] | 2023 |
|  |  |  | Pembrolizumab | Cisplatin + cytotoxics | Patients with locally advanced or metastatic HER2-negative gastric or gastro-esophageal junction adenocarcinoma | III | 1579 | NCT03675737 | mOS 12.9 mo | [S177] | 2023 |
|  |  |  | Toripalimab | Platinum + pemetrexed + nab-paclitaxel | Patients with potentially resectable non-driver gene mutation NSCLC | II | 18 | NCT04144608 | MPR 53.3%  pCR 40% | [S178] | 2021 |
|  |  |  | Nivolumab | Carboplatin + paclitaxel + veliparib or carboplatin + pemetrexed + veliparib | Patients with metastatic or advanced NSCLC | I | 25 | NCT02944396 | ORR 40%  OR 64% | [S179] | 2023 |
|  |  | Anti-IDO1 (+ anti-PD-1) | Epacadostat (+ pembrolizumab) | Platinum + pemetrexed or carboplatin + paclitaxel | Patients with metastatic NSCLC | II | 1062 | NCT03322566 | - | [S180] | 2018 |
|  |  |  | Epacadostat (+ pembrolizumab) | Platinum + cytotoxics | Patients with advanced or metastatic solid tumors | I/II | 70 | NCT03085914 | ORR 31.4% | [S181] | 2022 |
|  |  | Anti-CTLA-4 (+ anti-PD-1) | Ipilimumab + nivolumab | Cisplatin + pemetrexed or carboplatin + paclitaxel | Patients with advanced/metastatic NSCLC | II | 288 | NCT02659059 | ORR 30% | [S182] | 2019 |
|  |  | Anti-PD-L1 | Avelumab | Carboplatin + berzosertib | Patients with platinum-sensitive PARPi-resistant ovarian cancer | Ib/II | 3 | NCT03704467 | - | [S183] | 2019 |
|  |  |  | Durvalumab | Platinum + etoposide | Patients With untreated extensive-stage SCLC | III | 101 | NCT04712903  EudraCT 2020-002328-35 | ORR 51.5%  mPFS 6.1 mo  6-month PFS 50.2% | [S184] | 2022 |
|  |  |  | Durvalumab | Cisplatin + gemcitabine | Patients with advanced biliary tract cancer | III | 685 | NCT03875235 | 24-month OS 24.9%  ORR 26.7% | [S185] | 2022 |
|  |  | Anti-PD-L1 (+ anti-CTLA-4) | Durvalumab (+ tremelimumab) | Cisplatin + 5-FU | Patients with esophageal squamous cell carcinoma | Ib | 16 | NCT02658214 | OR 37.5%  mPFS 3.75 mo  mOS 9.69 mo | [S186] | 2023 |
|  |  | Anti-PD-1 and anti-TGF-β receptor | Bintrafusp alfa | Platinum doublets | Patients with stage IV NSCLC | I/II | 70 | NCT03840915 | - | [S187, 188] | 2021 |
|  | **Oncolytic virus** | Reovirus | Reovirus type 3 Dearing (RT3D, REOLYSIN, pelareorep) | Carboplatin + paclitaxel | Patients with advanced solid cancers with emphasis on HNSCC | I/II | 31 | - | CR 3.8%  PR 23.1%  SD 34.6%  DP 30.8% | [S189-191] | 2009 |
|  |  |  | Pelareorep | Carboplatin + paclitaxel | Patients with metastatic pancreatic adenocarcinoma | II | 36 | NCT01280058 | mPFS 4.9 mo | [S192] | 2016 |
|  |  |  | Pelareorep | Carboplatin + paclitaxel | Patients with advanced malignant melanoma | II | 14 | - | mPFS 5.2 mo  mOS 10.9 mo  1-year OS 43%  DCR 85% | [S193] | 2017 |
|  | **Cytokines** | IL-12 expressing plasmid | EGEN-001 (phIL-12-005/PPC) (formulated IL-12 plasmid) | Carboplatin + docetaxel | Patients with platinum-sensitive recurrent ovarian cancer | I | 13 | NCT00473954 | CR 17%  PR 33%  SD 42%  PD 8% | [S194] | 2013 |
|  |  | TNF inhibitors | Certolizumab | Cisplatin + pemetrexed | Patients with stage IV lung adenocarcinomas | I | 30 | NCT02120807 | mPFS 7.1 mo | [S195] | 2022 |
|  | **Cancer vaccines** | Dendritic cell-based vaccination | DCVAC/OvCa | Carboplatin + gemcitabine | Patients with platinum-sensitive ovarian cancer | II | 71 | NCT02107950 | mOS prolonged by 13.4 mo | [S196] | 2021 |
|  |  |  | Dendritic cell vaccination | Carboplatin + paclitaxel | Patients with metastatic endometrial cancer | I/II | 7 | NCT04212377 | No DLTs | [S197] | 2024 |

S1. Parmar, M. K., Ledermann, J. A., Colombo, N., du Bois, A., Delaloye, J. F., Kristensen, G. B., et al. (2003). Paclitaxel plus platinum-based chemotherapy versus conventional platinum-based chemotherapy in women with relapsed ovarian cancer: the ICON4/AGO-OVAR-2.2 trial. *Lancet, 361*(9375), 2099-2106, <https://doi.org/10.1016/s0140-6736(03)13718-x>.

S2. du Bois, A., Lück, H.-J., Meier, W., Adams, H.-P., Möbus, V., Costa, S., et al. (2003). A Randomized Clinical Trial of Cisplatin/Paclitaxel Versus Carboplatin/Paclitaxel as First-Line Treatment of Ovarian Cancer. *JNCI: Journal of the National Cancer Institute, 95*(17), 1320-1329, <https://doi.org/10.1093/jnci/djg036>.

S3. Ozols, R. F., Bundy, B. N., Greer, B. E., Fowler, J. M., Clarke-Pearson, D., Burger, R. A., et al. (2003). Phase III Trial of Carboplatin and Paclitaxel Compared With Cisplatin and Paclitaxel in Patients With Optimally Resected Stage III Ovarian Cancer: A Gynecologic Oncology Group Study. *Journal of Clinical Oncology, 21*(17), 3194-3200, <https://doi.org/10.1200/jco.2003.02.153>.

S4. Okamoto, I., Moriyama, E., Fujii, S., Kishi, H., Nomura, M., Goto, E., et al. (2005). Phase II study of carboplatin-paclitaxel combination chemotherapy in elderly patients with advanced non-small cell lung cancer. *Jpn J Clin Oncol, 35*(4), 188-194, <https://doi.org/10.1093/jjco/hyi059>.

S5. Monk, B. J., Sill, M. W., McMeekin, D. S., Cohn, D. E., Ramondetta, L. M., Boardman, C. H., et al. (2009). Phase III trial of four cisplatin-containing doublet combinations in stage IVB, recurrent, or persistent cervical carcinoma: a Gynecologic Oncology Group study. *J Clin Oncol, 27*(28), 4649-4655, <https://doi.org/10.1200/jco.2009.21.8909>.

S6. Pignata, S., Scambia, G., Ferrandina, G., Savarese, A., Sorio, R., Breda, E., et al. (2011). Carboplatin plus paclitaxel versus carboplatin plus pegylated liposomal doxorubicin as first-line treatment for patients with ovarian cancer: the MITO-2 randomized phase III trial. *J Clin Oncol, 29*(27), 3628-3635, <https://doi.org/10.1200/jco.2010.33.8566>.

S7. Cervantes-Ruiperez, A., Hoskins, P., Vergote, I., Eisenhauer, E. A., Ghatage, P., Carey, M., et al. (2013). Final results of OV16, a phase III randomized study of sequential cisplatin-topotecan and carboplatin-paclitaxel (CP) versus CP in first-line chemotherapy for advanced epithelial ovarian cancer (EOC): A GCIG study of NCIC CTG, EORTC-GCG, and GEICO. *Journal of Clinical Oncology, 31*(15_suppl), 5502-5502, <https://doi.org/10.1200/jco.2013.31.15_suppl.5502>.

S8. Hoskins, P., Vergote, I., Cervantes, A., Tu, D., Stuart, G., Zola, P., et al. (2010). Advanced Ovarian Cancer: Phase III Randomized Study of Sequential Cisplatin–Topotecan and Carboplatin–Paclitaxel vs Carboplatin–Paclitaxel. *JNCI: Journal of the National Cancer Institute, 102*(20), 1547-1556, <https://doi.org/10.1093/jnci/djq362>.

S9. Hoskins, P. J., Vergote, I., Stuart, G., Cervantes, A., Tu, D., Carey, M., et al. (2008). A phase III trial of cisplatin plus topotecan followed by paclitaxel plus carboplatin versus standard carboplatin plus paclitaxel as first-line chemotherapy in women with newly diagnosed advanced epithelial ovarian cancer (EOC) (OV.16). A Gynecologic Cancer Intergroup Study of the NCIC CTG, EORTC GCG, and GEICO. *Journal of Clinical Oncology, 26*(15_suppl), LBA5505-LBA5505, <https://doi.org/10.1200/jco.2008.26.15_suppl.lba5505>.

S10. Brotto, L., Brundage, M., Hoskins, P., Vergote, I., Cervantes, A., Casado, H. A., et al. (2016). Randomized study of sequential cisplatin-topotecan/carboplatin-paclitaxel versus carboplatin-paclitaxel: effects on quality of life. *Support Care Cancer, 24*(3), 1241-1249, <https://doi.org/10.1007/s00520-015-2873-8>.

S11. Mahner, S., Meier, W., du Bois, A., Brown, C., Lorusso, D., Dell'Anna, T., et al. (2015). Carboplatin and pegylated liposomal doxorubicin versus carboplatin and paclitaxel in very platinum-sensitive ovarian cancer patients: results from a subset analysis of the CALYPSO phase III trial. *Eur J Cancer, 51*(3), 352-358, <https://doi.org/10.1016/j.ejca.2014.11.017>.

S12. Abraham, J. E., Pinilla, K., Dayimu, A., Grybowicz, L., Demiris, N., Harvey, C., et al. (2024). The PARTNER trial of neoadjuvant olaparib in triple-negative breast cancer. *Nature, 629*, 1142–1148, <https://doi.org/10.1038/s41586-024-07384-2>.

S13. Ma, W. W., Zhu, M., Lam, E. T., Diamond, J. R., Dy, G. K., Fisher, G. A., et al. (2021). A phase I pharmacokinetic and safety study of Paclitaxel Injection Concentrate for Nano-dispersion (PICN) alone and in combination with carboplatin in patients with advanced solid malignancies and biliary tract cancers. *Cancer Chemother Pharmacol, 87*(6), 779-788, <https://doi.org/10.1007/s00280-021-04235-z>.

S14. Urup, T., Pawlak, W. Z., Petersen, P. M., Pappot, H., Rørth, M., & Daugaard, G. (2013). Treatment with docetaxel and cisplatin in advanced adrenocortical carcinoma, a phase II study. *Br J Cancer, 108*(10), 1994-1997, <https://doi.org/10.1038/bjc.2013.229>.

S15. Aparicio, A. M., Harzstark, A. L., Corn, P. G., Wen, S., Araujo, J. C., Tu, S. M., et al. (2013). Platinum-based chemotherapy for variant castrate-resistant prostate cancer. *Clin Cancer Res, 19*(13), 3621-3630, <https://doi.org/10.1158/1078-0432.Ccr-12-3791>.

S16. Aparicio, A., Shen, L., Tapia, E. L. N., Lu, J.-F., Chen, H.-C., Zhang, J., et al. (2015). A comprehensive molecular characterization of aggressive variant prostate cancer (PC). *Journal of Clinical Oncology, 33*(7_suppl), 149-149, <https://doi.org/10.1200/jco.2015.33.7_suppl.149>.

S17. Sharma, P., Kimler, B. F., O'Dea, A., Nye, L. E., Wang, Y. Y., Yoder, R., et al. (2019). Results of randomized phase II trial of neoadjuvant carboplatin plus docetaxel or carboplatin plus paclitaxel followed by AC in stage I-III triple-negative breast cancer (NCT02413320). *J Clin Oncol, 37*(15_suppl), 516-516, <https://doi.org/10.1200/JCO.2019.37.15_suppl.516>.

S18. Miao, J., Wang, L., Ong, E. H. W., Hu, C., Lin, S., Chen, X., et al. (2023). Effects of induction chemotherapy on nutrition status in locally advanced nasopharyngeal carcinoma: a multicentre prospective study. *J Cachexia Sarcopenia Muscle, 14*(2), 815-825, <https://doi.org/10.1002/jcsm.13196>.

S19. Miao, J., Wang, L., Hu, C., Lin, S., Tan, S. H., Ong, E. H. W., et al. (2019). A Multicenter Prospective Observational Study of Nutritional Status in Locally Advanced Nasopharynx Cancer Treated by Induction Chemotherapy and Chemoradiotherapy. *International Journal of Radiation Oncology, Biology, Physics, 105*(1), S212, <https://doi.org/10.1016/j.ijrobp.2019.06.288>.

S20. Le Chevalier, T., Brisgand, D., Douillard, J. Y., Pujol, J. L., Alberola, V., Monnier, A., et al. (1994). Randomized study of vinorelbine and cisplatin versus vindesine and cisplatin versus vinorelbine alone in advanced non-small-cell lung cancer: results of a European multicenter trial including 612 patients. *J Clin Oncol, 12*(2), 360-367, <https://doi.org/10.1200/jco.1994.12.2.360>.

S21. Adam, Z., Coupková, H., Kolek, V., Kucera, M., Loffelmann, L., Martinez, A., et al. (1995). Vinorelbine and cisplatin in the treatment of advanced non-small cell lung cancer: results of a multicenter Czech study. *Acta Med Austriaca, 22*(5), 120-124.

S22. Bennouna, J., Havel, L., Krzakowski, M., Kollmeier, J., Gervais, R., Dansin, E., et al. (2014). Oral vinorelbine plus cisplatin as first-line chemotherapy in nonsquamous non-small-cell lung cancer: final results of an International randomized phase II study (NAVotrial 01). *Clin Lung Cancer, 15*(4), 258-265, <https://doi.org/10.1016/j.cllc.2014.04.007>.

S23. Watanabe, N., Niho, S., Kirita, K., Umemura, S., Matsumoto, S., Yoh, K., et al. (2015). Vinorelbine and cisplatin in patients with advanced non-small cell lung cancer with interstitial pneumonia. *Anticancer Res, 35*(3), 1697-1701.

S24. Grossi, F., Jaśkiewicz, P., Ferreira, M., Czyżewicz, G., Kowalski, D., Ciuffreda, L., et al. (2021). Oral vinorelbine and cisplatin as first-line therapy for advanced squamous NSCLC patients: a prospective randomized international phase II study (NAVoTrial 03). *Ther Adv Med Oncol, 13*, <https://doi.org/10.1177/17588359211022905>.

S25. Sandler, A. B., Nemunaitis, J., Denham, C., von Pawel, J., Cormier, Y., Gatzemeier, U., et al. (2000). Phase III trial of gemcitabine plus cisplatin versus cisplatin alone in patients with locally advanced or metastatic non-small-cell lung cancer. *J Clin Oncol, 18*(1), 122-130, <https://doi.org/10.1200/jco.2000.18.1.122>.

S26. Ngan, R. K. C., Yiu, H. H. Y., Lau, W. H., Yau, S., Cheung, F. Y., Chan, T. M., et al. (2002). Combination gemcitabine and cisplatin chemotherapy for metastatic or recurrent nasopharyngeal carcinoma: report of a phase II study. *Annals of Oncology, 13*(8), 1252-1258, <https://doi.org/https://doi.org/10.1093/annonc/mdf200>.

S27. Scagliotti, G. V., Parikh, P., Pawel, J. v., Biesma, B., Vansteenkiste, J., Manegold, C., et al. (2023). Phase III Study Comparing Cisplatin Plus Gemcitabine With Cisplatin Plus Pemetrexed in Chemotherapy-Naive Patients With Advanced-Stage Non–Small-Cell Lung Cancer. *Journal of Clinical Oncology, 41*(14), 2458-2466, <https://doi.org/10.1200/jco.22.02544>.

S28. Fan, Y., Lin, N.-m., Ma, S.-l., Luo, L.-h., Fang, L., Huang, Z.-y., et al. (2010). Phase II trial of gemcitabine plus cisplatin in patients with advanced non-small cell lung cancer. *Acta Pharmacologica Sinica, 31*(6), 746-752, <https://doi.org/10.1038/aps.2010.50>.

S29. Valle, J., Wasan, H., Palmer, D. H., Cunningham, D., Anthoney, A., Maraveyas, A., et al. (2010). Cisplatin plus Gemcitabine versus Gemcitabine for Biliary Tract Cancer. *New England Journal of Medicine, 362*(14), 1273-1281, <https://doi.org/10.1056/NEJMoa0908721>.

S30. De Santis, M., Bellmunt, J., Mead, G., Kerst, J. M., Leahy, M., Maroto, P., et al. (2012). Randomized phase II/III trial assessing gemcitabine/carboplatin and methotrexate/carboplatin/vinblastine in patients with advanced urothelial cancer who are unfit for cisplatin-based chemotherapy: EORTC study 30986. *J Clin Oncol, 30*(2), 191-199, <https://doi.org/10.1200/jco.2011.37.3571>.

S31. Bellmunt, J., von der Maase, H., Mead, G. M., Skoneczna, I., De Santis, M., Daugaard, G., et al. (2012). Randomized phase III study comparing paclitaxel/cisplatin/gemcitabine and gemcitabine/cisplatin in patients with locally advanced or metastatic urothelial cancer without prior systemic therapy: EORTC Intergroup Study 30987. *J Clin Oncol, 30*(10), 1107-1113, <https://doi.org/10.1200/jco.2011.38.6979>.

S32. Oudard, S., Culine, S., Vano, Y., Goldwasser, F., Théodore, C., Nguyen, T., et al. (2015). Multicentre randomised phase II trial of gemcitabine+platinum, with or without trastuzumab, in advanced or metastatic urothelial carcinoma overexpressing Her2. *Eur J Cancer, 51*(1), 45-54, <https://doi.org/10.1016/j.ejca.2014.10.009>.

S33. Shroff, R. T., Javle, M. M., Xiao, L., Kaseb, A. O., Varadhachary, G. R., Wolff, R. A., et al. (2019). Gemcitabine, cisplatin, and nab-paclitaxel for the treatment of advanced biliary tract cancers: a phase 2 clinical trial. *JAMA Oncology, 5*(6), 824-830, <https://doi.org/10.1001/jamaoncol.2019.0270>.

S34. Ioka, T., Kanai, M., Kobayashi, S., Sakai, D., Eguchi, H., Baba, H., et al. (2023). Randomized phase III study of gemcitabine, cisplatin plus S-1 versus gemcitabine, cisplatin for advanced biliary tract cancer (KHBO1401- MITSUBA). *J Hepatobiliary Pancreat Sci, 30*(1), 102-110, <https://doi.org/10.1002/jhbp.1219>.

S35. McNamara, M. G., Bridgewater, J., Palmer, D. H., Faluyi, O., Wasan, H., Patel, A., et al. (2021). A Phase Ib Study of NUC-1031 in Combination with Cisplatin for the First-Line Treatment of Patients with Advanced Biliary Tract Cancer (ABC-08). *Oncologist, 26*(4), e669-e678, <https://doi.org/10.1002/onco.13598>.

S36. Nephew, K., Matei, D., Balch, C., Fang, F., & Schilder, J. DNA methylation inhibitors for chemotherapy resensitization of solid tumors. In *Proc 100th Annual Meeting of the American Association for Cancer Research, Denver, CO, 2009 (Abstract 25), 2009*

S37. Tsimberidou, A. M., Said, R., Culotta, K., Wistuba, I., Jelinek, J., Fu, S., et al. (2015). Phase I study of azacitidine and oxaliplatin in patients with advanced cancers that have relapsed or are refractory to any platinum therapy. *Clin Epigenetics, 7*(1), 29, <https://doi.org/10.1186/s13148-015-0065-5>.

S38. Oza, A. M., Matulonis, U. A., Alvarez Secord, A., Nemunaitis, J., Roman, L. D., Blagden, S. P., et al. (2020). A Randomized Phase II Trial of Epigenetic Priming with Guadecitabine and Carboplatin in Platinum-resistant, Recurrent Ovarian Cancer. *Clin Cancer Res, 26*(5), 1009-1016, <https://doi.org/10.1158/1078-0432.Ccr-19-1638>.

S39. Kerger, J. N., Lebrun, F., Gil, T., Gombos, A., Ridoine, A., Bustin, F., et al. (2014). Phase II trial of oxaliplatin and 5-FU in patients (pts) with platinum-resistant recurrent (PRR) ovarian carcinoma (OVCA). *Journal of Clinical Oncology, 32*(15_suppl), 5580-5580, <https://doi.org/10.1200/jco.2014.32.15_suppl.5580>.

S40. Shah, M. A., Janjigian, Y. Y., Stoller, R., Shibata, S., Kemeny, M., Krishnamurthi, S., et al. (2015). Randomized Multicenter Phase II Study of Modified Docetaxel, Cisplatin, and Fluorouracil (DCF) Versus DCF Plus Growth Factor Support in Patients With Metastatic Gastric Adenocarcinoma: A Study of the US Gastric Cancer Consortium. *J Clin Oncol, 33*(33), 3874-3879, <https://doi.org/10.1200/jco.2015.60.7465>.

S41. Le, D. T., Laheru, D. A., Purtell, K., Uram, J. N., Wang, H., Lawrence, S., et al. (2014). A phase II trial of low-dose multiagent chemotherapy with gemcitabine, docetaxel, capecitabine, and cisplatin (GTX-C) in patients with metastatic pancreatic cancer. *Journal of Clinical Oncology, 32*(3_suppl), 213-213, <https://doi.org/10.1200/jco.2014.32.3_suppl.213>.

S42. Wilbur, H. C., Durham, J. N., Lim, S. J., Purtell, K., Bever, K. M., Laheru, D. A., et al. (2023). Gemcitabine, docetaxel, capecitabine, cisplatin, irinotecan as first-line treatment for metastatic pancreatic cancer. *Cancer Res Commun, 3*(8), 1672-1677, <https://doi.org/10.1158/2767-9764.Crc-23-0230>.

S43. Schmoll, H. J., Tabernero, J., Maroun, J., de Braud, F., Price, T., Van Cutsem, E., et al. (2015). Capecitabine Plus Oxaliplatin Compared With Fluorouracil/Folinic Acid As Adjuvant Therapy for Stage III Colon Cancer: Final Results of the NO16968 Randomized Controlled Phase III Trial. *J Clin Oncol, 33*(32), 3733-3740, <https://doi.org/10.1200/jco.2015.60.9107>.

S44. Yoon, S., Yoo, C., Ryu, M.-H., Kang, M. J., Ryoo, B.-Y., Park, S. R., et al. (2017). Phase 2 study of adjuvant chemotherapy with docetaxel, capecitabine, and cisplatin in patients with curatively resected stage IIIB–IV gastric cancer. *Gastric Cancer, 20*(1), 182-189, <https://doi.org/10.1007/s10120-015-0580-2>.

S45. André, T., Boni, C., Mounedji-Boudiaf, L., Navarro, M., Tabernero, J., Hickish, T., et al. (2004). Oxaliplatin, fluorouracil, and leucovorin as adjuvant treatment for colon cancer. *N Engl J Med, 350*(23), 2343-2351, <https://doi.org/10.1056/NEJMoa032709>.

S46. Kalofonos, H. P., Aravantinos, G., Kosmidis, P., Papakostas, P., Economopoulos, T., Dimopoulos, M., et al. (2005). Irinotecan or oxaliplatin combined with leucovorin and 5-fluorouracil as first-line treatment in advanced colorectal cancer: a multicenter, randomized, phase II study. *Ann Oncol, 16*(6), 869-877, <https://doi.org/10.1093/annonc/mdi193>.

S47. Vogelzang, N. J., Rusthoven, J. J., Symanowski, J., Denham, C., Kaukel, E., Ruffie, P., et al. (2023). Phase III Study of Pemetrexed in Combination With Cisplatin Versus Cisplatin Alone in Patients With Malignant Pleural Mesothelioma. *J Clin Oncol, 41*(12), 2125-2133, <https://doi.org/10.1200/jco.22.02542>.

S48. van Meerbeeck, J. P., Gaafar, R., Manegold, C., Van Klaveren, R. J., Van Marck, E. A., Vincent, M., et al. (2005). Randomized phase III study of cisplatin with or without raltitrexed in patients with malignant pleural mesothelioma: an intergroup study of the European Organisation for Research and Treatment of Cancer Lung Cancer Group and the National Cancer Institute of Canada. *J Clin Oncol, 23*(28), 6881-6889, <https://doi.org/10.1200/jco.20005.14.589>.

S49. Ceresoli, G. L., Zucali, P. A., Favaretto, A. G., Grossi, F., Bidoli, P., Del Conte, G., et al. (2006). Phase II study of pemetrexed plus carboplatin in malignant pleural mesothelioma. *J Clin Oncol, 24*(9), 1443-1448, <https://doi.org/10.1200/jco.2005.04.3190>.

S50. Obasaju, C. K., Ye, Z., Wozniak, A. J., Belani, C. P., Keohan, M. L., Ross, H. J., et al. (2007). Single-arm, open label study of pemetrexed plus cisplatin in chemotherapy naïve patients with malignant pleural mesothelioma: outcomes of an expanded access program. *Lung Cancer, 55*(2), 187-194, <https://doi.org/10.1016/j.lungcan.2006.09.023>.

S51. Matulonis, U. A., Horowitz, N. S., Campos, S. M., Lee, H., Lee, J., Krasner, C. N., et al. (2008). Phase II study of carboplatin and pemetrexed for the treatment of platinum-sensitive recurrent ovarian cancer. *J Clin Oncol, 26*(35), 5761-5766, <https://doi.org/10.1200/jco.2008.17.0282>.

S52. Castagneto, B., Botta, M., Aitini, E., Spigno, F., Degiovanni, D., Alabiso, O., et al. (2008). Phase II study of pemetrexed in combination with carboplatin in patients with malignant pleural mesothelioma (MPM). *Ann Oncol, 19*(2), 370-373, <https://doi.org/10.1093/annonc/mdm501>.

S53. Schuette, W. H., Gröschel, A., Sebastian, M., Andreas, S., Müller, T., Schneller, F., et al. (2013). A randomized phase II study of pemetrexed in combination with cisplatin or carboplatin as first-line therapy for patients with locally advanced or metastatic non-small-cell lung cancer. *Clin Lung Cancer, 14*(3), 215-223, <https://doi.org/10.1016/j.cllc.2012.10.001>.

S54. Clamp, A. R., Schöffski, P., Valle, J. W., Wilson, R. H., Marreaud, S., Govaerts, A. S., et al. (2008). A phase I and pharmacokinetic study of OSI-7904L, a liposomal thymidylate synthase inhibitor in combination with oxaliplatin in patients with advanced colorectal cancer. *Cancer Chemother Pharmacol, 61*(4), 579-585, <https://doi.org/10.1007/s00280-007-0509-5>.

S55. Theodore, C., Skoneczna, I., Bodrogi, I., Leahy, M., Kerst, J. M., Collette, L., et al. (2008). A phase II multicentre study of irinotecan (CPT 11) in combination with cisplatin (CDDP) in metastatic or locally advanced penile carcinoma (EORTC PROTOCOL 30992). *Ann Oncol, 19*(7), 1304-1307, <https://doi.org/10.1093/annonc/mdn149>.

S56. Takano, M., Yamamoto, K., Tabata, T., Minegishi, Y., Yokoyama, T., Hirata, E., et al. (2016). Impact of UGT1A1 genotype upon toxicities of combination with low-dose irinotecan plus platinum. *Asia-Pacific Journal of Clinical Oncology, 12*(2), 115-124, <https://doi.org/https://doi.org/10.1111/ajco.12453>.

S57. Christenson, E. S., Lim, S. J., Durham, J., De Jesus-Acosta, A., Bever, K., Laheru, D., et al. (2022). Cell-free DNA predicts prolonged response to multi-agent chemotherapy in pancreatic ductal adenocarcinoma. *Cancer Res Commun, 2*(11), 1418-1425, <https://doi.org/10.1158/2767-9764.Crc-22-0343>.

S58. O'Brien, M. E., Konopa, K., Lorigan, P., Bosquee, L., Marshall, E., Bustin, F., et al. (2011). Randomised phase II study of amrubicin as single agent or in combination with cisplatin versus cisplatin etoposide as first-line treatment in patients with extensive stage small cell lung cancer - EORTC 08062. *Eur J Cancer, 47*(15), 2322-2330, <https://doi.org/10.1016/j.ejca.2011.05.020>.

S59. Spigel, D. R., Hainsworth, J. D., Shipley, D. L., Mekhail, T. M., Zubkus, J. D., Waterhouse, D. M., et al. (2018). Amrubicin and carboplatin with pegfilgrastim in patients with extensive stage small cell lung cancer: a phase II trial of the Sarah Cannon Oncology Research Consortium. *Lung Cancer, 117*, 38-43, <https://doi.org/10.1016/j.lungcan.2018.01.007>.

S60. de Wit, R., Skoneczna, I., Daugaard, G., De Santis, M., Garin, A., Aass, N., et al. (2012). Randomized phase III study comparing paclitaxel-bleomycin, etoposide, and cisplatin (BEP) to standard BEP in intermediate-prognosis germ-cell cancer: intergroup study EORTC 30983. *J Clin Oncol, 30*(8), 792-799, <https://doi.org/10.1200/jco.2011.37.0171>.

S61. Sternberg, C. N., Skoneczna, I. A., Kerst, J. M., Fossa, S. D., Albers, P., Agerbaek, M., et al. (2014). Final results of EORTC intergroup randomized phase III trial comparing immediate versus deferred chemotherapy after radical cystectomy in patients with pT3T4 and/or N+ M0 transitional cell carcinoma (TCC) of the bladder. *Journal of Clinical Oncology, 32*(15_suppl), 4500-4500, <https://doi.org/10.1200/jco.2014.32.15_suppl.4500>.

S62. Power, P., Stuart, G., Oza, A., Provencher, D., Bentley, J. R., Miller, W. H., Jr., et al. (2009). Efficacy of pegylated liposomal doxorubicin (PLD) plus carboplatin in ovarian cancer patients who recur within six to twelve months: a phase II study. *Gynecol Oncol, 114*(3), 410-414, <https://doi.org/10.1016/j.ygyno.2009.04.037>.

S63. Bröckelmann, P. J., Müller, H., Casasnovas, O., Hutchings, M., von Tresckow, B., Jürgens, M., et al. (2017). Risk factors and a prognostic score for survival after autologous stem-cell transplantation for relapsed or refractory Hodgkin lymphoma. *Ann Oncol, 28*(6), 1352-1358, <https://doi.org/10.1093/annonc/mdx072>.

S64. Josting, A., Müller, H., Borchmann, P., Baars, J. W., Metzner, B., Döhner, H., et al. (2010). Dose intensity of chemotherapy in patients with relapsed Hodgkin's lymphoma. *J Clin Oncol, 28*(34), 5074-5080, <https://doi.org/10.1200/jco.2010.30.5771>.

S65. Daugaard, G., Skoneczna, I., Aass, N., De Wit, R., De Santis, M., Dumez, H., et al. (2011). A randomized phase III study comparing standard dose BEP with sequential high-dose cisplatin, etoposide, and ifosfamide (VIP) plus stem-cell support in males with poor-prognosis germ-cell cancer. An intergroup study of EORTC, GTCSG, and Grupo Germinal (EORTC 30974). *Ann Oncol, 22*(5), 1054-1061, <https://doi.org/10.1093/annonc/mdq575>.

S66. Tan, Y. Y., Al-Bubseeree, B., Irvine, D., MacDonald, G., McQuaker, G., Parker, A., et al. (2019). High-dose chemotherapy in relapsed or refractory metastatic germ-cell cancer: the Scotland experience. *Clin Genitourin Cancer, 17*(2), 125-131, <https://doi.org/10.1016/j.clgc.2018.11.013>.

S67. Giaccone, G., Herbst, R. S., Manegold, C., Scagliotti, G., Rosell, R., Miller, V., et al. (2004). Gefitinib in combination with gemcitabine and cisplatin in advanced non-small-cell lung cancer: a phase III trial--INTACT 1. *J Clin Oncol, 22*(5), 777-784, <https://doi.org/10.1200/jco.2004.08.001>.

S68. Miller, V. A., Johnson, D. H., Krug, L. M., Pizzo, B., Tyson, L., Perez, W., et al. (2003). Pilot trial of the epidermal growth factor receptor tyrosine kinase inhibitor gefitinib plus carboplatin and paclitaxel in patients with stage IIIB or IV non-small-cell lung cancer. *J Clin Oncol, 21*(11), 2094-2100, <https://doi.org/10.1200/jco.2003.12.008>.

S69. Herbst, R. S., Prager, D., Hermann, R., Fehrenbacher, L., Johnson, B. E., Sandler, A., et al. (2005). TRIBUTE: a phase III trial of erlotinib hydrochloride (OSI-774) combined with carboplatin and paclitaxel chemotherapy in advanced non-small-cell lung cancer. *J Clin Oncol, 23*(25), 5892-5899, <https://doi.org/10.1200/jco.2005.02.840>.

S70. Gatzemeier, U., Pluzanska, A., Szczesna, A., Kaukel, E., Roubec, J., De Rosa, F., et al. (2007). Phase III study of erlotinib in combination with cisplatin and gemcitabine in advanced non-small-cell lung cancer: the Tarceva Lung Cancer Investigation Trial. *J Clin Oncol, 25*(12), 1545-1552, <https://doi.org/10.1200/jco.2005.05.1474>.

S71. Lee, J. S., Ignacio, J., Yu, C., Zhou, C., Wu, Y., Chen, Y., et al. (2008). FAST-ACT: A phase II randomized double-blind trial of sequential erlotinib and chemotherapy as first-line treatment in patients (pts) with stage IIIB/IV non-small cell lung cancer (NSCLC). *Journal of Clinical Oncology, 26*(15_suppl), 8031-8031, <https://doi.org/10.1200/jco.2008.26.15_suppl.8031>.

S72. Wu, Y. L., Lee, J. S., Thongprasert, S., Yu, C. J., Zhang, L., Ladrera, G., et al. (2013). Intercalated combination of chemotherapy and erlotinib for patients with advanced stage non-small-cell lung cancer (FASTACT-2): a randomised, double-blind trial. *Lancet Oncol, 14*(8), 777-786, <https://doi.org/10.1016/s1470-2045(13)70254-7>.

S73. William, W. N., Feng, L., Kies, M. S., Ahmed, S., Blumenschein, G. R., Glisson, B. S., et al. (2017). Randomized, double-blind, placebo-controlled, phase II trial of first-line platinum/docetaxel with or without erlotinib (E) in patients (pts) with recurrent and/or metastatic (R/M) head and neck squamous cell carcinomas (HNSCCs). *Journal of Clinical Oncology, 35*(15_suppl), 6017-6017, <https://doi.org/10.1200/JCO.2017.35.15_suppl.6017>.

S74. Kozloff, M. F., Martin, L. P., Krzakowski, M., Samuel, T. A., Rado, T. A., Arriola, E., et al. (2012). Phase I trial of axitinib combined with platinum doublets in patients with advanced non-small cell lung cancer and other solid tumours. *British Journal of Cancer, 107*(8), 1277-1285, <https://doi.org/10.1038/bjc.2012.406>.

S75. Fountzilas, C., Adjei, A., Opyrchal, M., Evans, R., Ghasemi, M., Attwood, K., et al. (2021). A phase I study of the anaplastic lymphoma kinase inhibitor ceritinib in combination with gemcitabine-based chemotherapy in patients with advanced solid tumors. *International Journal of Cancer, 149*(12), 2063-2074, <https://doi.org/https://doi.org/10.1002/ijc.33754>.

S76. Leijen, S., van Geel, R. M., Sonke, G. S., de Jong, D., Rosenberg, E. H., Marchetti, S., et al. (2016). Phase II Study of WEE1 Inhibitor AZD1775 Plus Carboplatin in Patients With TP53-Mutated Ovarian Cancer Refractory or Resistant to First-Line Therapy Within 3 Months. *J Clin Oncol, 34*(36), 4354-4361, <https://doi.org/10.1200/jco.2016.67.5942>.

S77. Mendez, E., Rodriguez, C. P., Kao, M., Harbison, R. A., Martins, R. G., Futran, N. D., et al. (2017). A phase I clinical trial of AZD1775 in combination with neoadjuvant weekly cisplatin and docetaxel in borderline resectable head and neck squamous cell carcinoma (HNSCC). *Journal of Clinical Oncology, 35*(15_suppl), 6034-6034, <https://doi.org/10.1200/JCO.2017.35.15_suppl.6034>.

S78. Oza, A. M., Estevez-Diz, M., Grischke, E. M., Hall, M., Marmé, F., Provencher, D., et al. (2020). A Biomarker-enriched, randomized phase II trial of adavosertib (AZD1775) plus paclitaxel and carboplatin for women with platinum-sensitive TP53-mutant ovarian cancer. *Clin Cancer Res, 26*(18), 4767-4776, <https://doi.org/10.1158/1078-0432.Ccr-20-0219>.

S79. Curigliano, G., Shapiro, G. I., Kristeleit, R. S., Abdul Razak, A. R., Leong, S., Alsina, M., et al. (2023). A Phase 1B open-label study of gedatolisib (PF-05212384) in combination with other anti-tumour agents for patients with advanced solid tumours and triple-negative breast cancer. *Br J Cancer, 128*(1), 30-41, <https://doi.org/10.1038/s41416-022-02025-9>.

S80. Wainberg, Z. A., Shapiro, G., Curigliano, G., Kristeleit, R. S., Leong, S., Alsina, M., et al. (2016). Phase I study of the PI3K/mTOR inhibitor gedatolisib (PF-05212384) in combination with docetaxel, cisplatin, and dacomitinib. *Journal of Clinical Oncology, 34*(15_suppl), 2566-2566, <https://doi.org/10.1200/JCO.2016.34.15_suppl.2566>.

S81. Swiecicki, P. L., Durm, G., Bellile, E., Bhangale, A., Brenner, J. C., & Worden, F. P. (2020). A multi-center phase II trial evaluating the efficacy of palbociclib in combination with carboplatin for the treatment of unresectable recurrent or metastatic head and neck squamous cell carcinoma. *Invest New Drugs, 38*(5), 1550-1558, <https://doi.org/10.1007/s10637-020-00898-2>.

S82. Coffman, L. G., Orellana, T. J., Liu, T., Frisbie, L. G., Normolle, D., Griffith, K., et al. (2022). Phase I trial of ribociclib with platinum chemotherapy in ovarian cancer. *JCI Insight, 7*(18), <https://doi.org/10.1172/jci.insight.160573>.

S83. Sun, W., Powell, M., O'Dwyer, P. J., Catalano, P., Ansari, R. H., & Benson, A. B., 3rd (2010). Phase II study of sorafenib in combination with docetaxel and cisplatin in the treatment of metastatic or advanced gastric and gastroesophageal junction adenocarcinoma: ECOG 5203. *J Clin Oncol, 28*(18), 2947-2951, <https://doi.org/10.1200/jco.2009.27.7988>.

S84. du Bois, A., Huober, J., Stopfer, P., Pfisterer, J., Wimberger, P., Loibl, S., et al. (2010). A phase I open-label dose-escalation study of oral BIBF 1120 combined with standard paclitaxel and carboplatin in patients with advanced gynecological malignancies. *Ann Oncol, 21*(2), 370-375, <https://doi.org/10.1093/annonc/mdp506>.

S85. Del Campo, J. M., Pardo Búrdalo, B., Rodriguez Freixinos, V., Gaba Garcia, L., Gil Martín, M., Oaknin, A., et al. (2014). 889P - Phase I Dose-Escalation Study to Determine the Maximum Tolerated Dose (Mtd) of Nintedanib (Bibf 1120) in Combination with Carboplatin/Pegylated Liposomal Doxorubicin (Pld) in Patients (Pts) with Recurrent Ovarian Cancer (Roc). *Annals of Oncology, 25*, iv310, <https://doi.org/https://doi.org/10.1093/annonc/mdu338.15>.

S86. du Bois, A., Vergote, I., Wimberger, P., Ray-Coquard, I., Harter, P., Curtis, L. B., et al. (2012). Open-label feasibility study of pazopanib, carboplatin, and paclitaxel in women with newly diagnosed, untreated, gynaecologic tumours: a phase I/II trial of the AGO study group. *Br J Cancer, 106*(4), 629-632, <https://doi.org/10.1038/bjc.2011.608>.

S87. Ready, N. E., Pang, H. H., Gu, L., Otterson, G. A., Thomas, S. P., Miller, A. A., et al. (2015). Chemotherapy With or Without Maintenance Sunitinib for Untreated Extensive-Stage Small-Cell Lung Cancer: A Randomized, Double-Blind, Placebo-Controlled Phase II Study-CALGB 30504 (Alliance). *J Clin Oncol, 33*(15), 1660-1665, <https://doi.org/10.1200/jco.2014.57.3105>.

S88. Moehler, M., Schad, A., Maderer, A., Atasoy, A., Mauer, M. E., Caballero, C., et al. (2018). Lapatinib with ECF/X in the first-line treatment of metastatic gastric cancer according to HER2neu and EGFR status: a randomized placebo-controlled phase II study (EORTC 40071). *Cancer Chemother Pharmacol, 82*(4), 733-739, <https://doi.org/10.1007/s00280-018-3667-8>.

S89. Trédaniel, J., Descourt, R., Moro-Sibilot, D., Misset, J., Gachard, E., Garcia-Vargas, J., et al. (2009). Vorinostat in combination with gemcitabine and cisplatinum in patients with advanced non-small cell lung cancer (NSCLC): A Phase I dose-escalation study. *Journal of Clinical Oncology, 27*(15_suppl), 8049-8049, <https://doi.org/10.1200/jco.2009.27.15_suppl.8049>.

S90. Ramalingam, S. S., Maitland, M. L., Frankel, P., Argiris, A. E., Koczywas, M., Gitlitz, B., et al. (2010). Carboplatin and Paclitaxel in combination with either vorinostat or placebo for first-line therapy of advanced non-small-cell lung cancer. *J Clin Oncol, 28*(1), 56-62, <https://doi.org/10.1200/jco.2009.24.9094>.

S91. Tjulandin, S., Fedyanin, M., Vladimirov, V. I., Kostorov, V., Lisyanskaya, A. S., Krikunova, L., et al. (2017). A multicenter phase II study of the efficacy and safety of quisinostat (an HDAC inhibitor) in combination with paclitaxel and carboplatin chemotherapy (CT) in patients (pts) with recurrent platinum resistant high grade serous epithelial ovarian, primarily peritoneal or fallopian tube carcinoma cancer (OC). *J Clin Oncol, 35*(15_suppl), 5541-5541, <https://doi.org/10.1200/JCO.2017.35.15_suppl.5541>.

S92. Meteran, H., Knudsen, A., Jørgensen, T. L., Nielsen, D., & Herrstedt, J. (2024). Carboplatin plus paclitaxel in combination with the histone deacetylate inhibitor, vorinostat, in patients with recurrent platinum-sensitive ovarian cancer. *J Clin Med, 13*(3), 897, <https://doi.org/10.3390/jcm13030897>.

S93. Kelly, M. G., Mor, G., Husband, A., O'Malley, D. M., Baker, L., Azodi, M., et al. (2011). Phase II evaluation of phenoxodiol in combination with cisplatin or paclitaxel in women with platinum/taxane-refractory/resistant epithelial ovarian, fallopian tube, or primary peritoneal cancers. *Int J Gynecol Cancer, 21*(4), 633-639, <https://doi.org/10.1097/IGC.0b013e3182126f05>.

S94. O'Brien, M. E., Gaafar, R. M., Popat, S., Grossi, F., Price, A., Talbot, D. C., et al. (2013). Phase II study of first-line bortezomib and cisplatin in malignant pleural mesothelioma and prospective validation of progression free survival rate as a primary end-point for mesothelioma clinical trials (European Organisation for Research and Treatment of Cancer 08052). *Eur J Cancer, 49*(13), 2815-2822, <https://doi.org/10.1016/j.ejca.2013.05.008>.

S95. Bahleda, R., Sessa, C., Del Conte, G., Gianni, L., Capri, G., Varga, A., et al. (2014). Phase I clinical and pharmacokinetic study of ombrabulin (AVE8062) combined with cisplatin/docetaxel or carboplatin/paclitaxel in patients with advanced solid tumors. *Invest New Drugs, 32*(6), 1188-1196, <https://doi.org/10.1007/s10637-014-0119-0>.

S96. Wang, B. Y., Zhang, J., Wang, J. L., Sun, S., Wang, Z. H., Wang, L. P., et al. (2015). Erratum to: Intermittent high dose proton pump inhibitor enhances the antitumor effects of chemotherapy in metastatic breast cancer. *J Exp Clin Cancer Res, 34*, 109, <https://doi.org/10.1186/s13046-015-0220-z>.

S97. Miller, K., Tong, Y., Jones, D. R., Walsh, T., Danso, M. A., Ma, C. X., et al. (2015). Cisplatin with or without rucaparib after preoperative chemotherapy in patients with triple negative breast cancer: final efficacy results of Hoosier Oncology Group BRE09-146. *J Clin Oncol, 33*(15_suppl), 1082-1082, <https://doi.org/10.1200/jco.2015.33.15_suppl.1082>.

S98. Lee, J. M., Peer, C. J., Yu, M., Amable, L., Gordon, N., Annunziata, C. M., et al. (2017). Sequence-specific pharmacokinetic and pharmacodynamic phase I/Ib study of Olaparib tablets and carboplatin in women's cancer. *Clin Cancer Res, 23*(6), 1397-1406, <https://doi.org/10.1158/1078-0432.Ccr-16-1546>.

S99. Gray, H. J., Bell-McGuinn, K., Fleming, G. F., Cristea, M., Xiong, H., Sullivan, D., et al. (2018). Phase I combination study of the PARP inhibitor veliparib plus carboplatin and gemcitabine in patients with advanced ovarian cancer and other solid malignancies. *Gynecol Oncol, 148*(3), 507-514, <https://doi.org/10.1016/j.ygyno.2017.12.029>.

S100. Loibl, S., O'Shaughnessy, J., Untch, M., Sikov, W. M., Rugo, H. S., McKee, M. D., et al. (2018). Addition of the PARP inhibitor veliparib plus carboplatin or carboplatin alone to standard neoadjuvant chemotherapy in triple-negative breast cancer (BrighTNess): a randomised, phase 3 trial. *Lancet Oncol, 19*(4), 497-509, <https://doi.org/10.1016/s1470-2045(18)30111-6>.

S101. Geyer, C. E., Sikov, W. M., Huober, J., Rugo, H. S., Wolmark, N., O'Shaughnessy, J., et al. (2022). Long-term efficacy and safety of addition of carboplatin with or without veliparib to standard neoadjuvant chemotherapy in triple-negative breast cancer: 4-year follow-up data from BrighTNess, a randomized phase III trial. *Ann Oncol, 33*(4), 384-394, <https://doi.org/10.1016/j.annonc.2022.01.009>.

S102. Owonikoko, T. K., Dahlberg, S. E., Sica, G. L., Wagner, L. I., Wade, J. L., 3rd, Srkalovic, G., et al. (2019). Randomized phase II trial of cisplatin and etoposide in combination with veliparib or placebo for extensive-stage small-cell lung cancer: ECOG-ACRIN 2511 study. *J Clin Oncol, 37*(3), 222-229, <https://doi.org/10.1200/jco.18.00264>.

S103. Jelinek, M. J., Foster, N. R., Zoroufy, A. J., Schwartz, G. K., Munster, P. N., Seiwert, T. Y., et al. (2021). A phase I trial adding poly(ADP-ribose) polymerase inhibitor veliparib to induction carboplatin-paclitaxel in patients with head and neck squamous cell carcinoma: Alliance A091101. *Oral Oncol, 114*, 105171, <https://doi.org/10.1016/j.oraloncology.2020.105171>.

S104. Ayoub, J. P., Wildiers, H., Friedlander, M., Arun, B. K., Han, H. S., Puhalla, S., et al. (2021). Safety and efficacy of veliparib plus carboplatin/paclitaxel in patients with HER2-negative metastatic or locally advanced breast cancer: subgroup analyses by germline BRCA1/2 mutations and hormone receptor status from the phase-3 BROCADE3 trial. *Ther Adv Med Oncol, 13*, <https://doi.org/10.1177/17588359211059601>.

S105. Penson, R. T., Ambrosio, A. J., Whalen, C. A., Krasner, C. N., Konstantinopoulos, P. A., Bradley, C., et al. (2023). Phase II trials of iniparib (BSI-201) in combination with gemcitabine and carboplatin in patients with recurrent ovarian cancer. *Oncologist, 28*(3), 252-257, <https://doi.org/10.1093/oncolo/oyac275>.

S106. Beddowes, E., Spicer, J., Chan, P. Y., Khadeir, R., Corbacho, J. G., Repana, D., et al. (2017). Phase 1 Dose-Escalation Study of Pegylated Arginine Deiminase, Cisplatin, and Pemetrexed in Patients With Argininosuccinate Synthetase 1-Deficient Thoracic Cancers. *J Clin Oncol, 35*(16), 1778-1785, <https://doi.org/10.1200/jco.2016.71.3230>.

S107. Szlosarek, P. W., Baas, P., Ceresoli, G. L., Fennell, D. A., Gilligan, D., Johnston, A., et al. (2017). ATOMIC-Meso: A randomized phase 2/3 trial of ADI-PEG20 or placebo with pemetrexed and cisplatin in patients with argininosuccinate synthetase 1-deficient non-epithelioid mesothelioma. *Journal of Clinical Oncology, 35*(15_suppl), TPS8582-TPS8582, <https://doi.org/10.1200/JCO.2017.35.15_suppl.TPS8582>.

S108. Szlosarek, P. W., Creelan, B. C., Sarkodie, T., Nolan, L., Taylor, P., Olevsky, O., et al. (2024). Pegargiminase Plus First-Line Chemotherapy in Patients With Nonepithelioid Pleural Mesothelioma: The ATOMIC-Meso Randomized Clinical Trial. *JAMA Oncology*, <https://doi.org/10.1001/jamaoncol.2023.6789>.

S109. Hall, P. E., Lewis, R., Syed, N., Shaffer, R., Evanson, J., Ellis, S., et al. (2019). A Phase I Study of Pegylated Arginine Deiminase (Pegargiminase), Cisplatin, and Pemetrexed in Argininosuccinate Synthetase 1-Deficient Recurrent High-grade Glioma. *Clinical Cancer Research, 25*(9), 2708-2716, <https://doi.org/10.1158/1078-0432.Ccr-18-3729>.

S110. Moore, K. N., Gunderson, C. C., Sabbatini, P., McMeekin, D. S., Mantia-Smaldone, G., Burger, R. A., et al. (2019). A phase 1b dose escalation study of ipafricept (OMP54F28) in combination with paclitaxel and carboplatin in patients with recurrent platinum-sensitive ovarian cancer. *Gynecol Oncol, 154*(2), 294-301, <https://doi.org/10.1016/j.ygyno.2019.04.001>.

S111. Swiecicki, P. L., Bellile, E., Casper, K., Chinn, S. B., Dragovic, A. F., Jolly, S., et al. (2022). Randomized trial of laryngeal organ preservation evaluating two cycles of induction chemotherapy with platinum, docetaxel, and a novel Bcl-xL inhibitor. *Head Neck, 44*(7), 1509-1519, <https://doi.org/10.1002/hed.27043>.

S112. Swiecicki, P., Bellile, E., Casper, K., Malloy, K. M., Kupfer, R., Spector, M. E., et al. (2019). A randomized trial of laryngeal organ preservation evaluating two cycles of induction chemotherapy with platinum, docetaxel, and a novel Bcl-xL inhibitor. *Journal of Clinical Oncology, 37*(15_suppl), 6066-6066, <https://doi.org/10.1200/JCO.2019.37.15_suppl.6066>.

S113. Koczywas, M., Frankel, P. H., Riess, J. W., El-Khoueiry, A. B., Villaruz, L. C., Leong, S., et al. (2020). Phase I study of TRC102 in combination with cisplatin and pemetrexed in patients with advanced solid tumors/Phase II study of TRC102 with pemetrexed in patients with mesothelioma refractory to pemetrexed and cisplatin or carboplatin. *J Clin Oncol, 38*(15_suppl), 9055-9055, <https://doi.org/10.1200/JCO.2020.38.15_suppl.9055>.

S114. Thienelt, C. D., Bunn, P. A., Jr., Hanna, N., Rosenberg, A., Needle, M. N., Long, M. E., et al. (2005). Multicenter phase I/II study of cetuximab with paclitaxel and carboplatin in untreated patients with stage IV non-small-cell lung cancer. *J Clin Oncol, 23*(34), 8786-8793, <https://doi.org/10.1200/jco.2005.03.1997>.

S115. Secord, A. A., Blessing, J. A., Armstrong, D. K., Rodgers, W. H., Miner, Z., Barnes, M. N., et al. (2008). Phase II trial of cetuximab and carboplatin in relapsed platinum-sensitive ovarian cancer and evaluation of epidermal growth factor receptor expression: a Gynecologic Oncology Group study. *Gynecol Oncol, 108*(3), 493-499, <https://doi.org/10.1016/j.ygyno.2007.11.029>.

S116. Vermorken, J. B., Mesia, R., Rivera, F., Remenar, E., Kawecki, A., Rottey, S., et al. (2008). Platinum-Based Chemotherapy plus Cetuximab in Head and Neck Cancer. *New England Journal of Medicine, 359*(11), 1116-1127, <https://doi.org/10.1056/NEJMoa0802656>.

S117. Schwartzberg, L. S., Tauer, K., Atkins, J., Sivarajan, K., Patel, V., Bastos, B., et al. (2012). LBA30 - Elung: A Multicenter, Randomized Phase IIB Trial of “Standard” Platinum Doublets Plus Cetuximab (CET) as First-Line Treatment of Recurrent or Advanced Non-Small Cell Lung Cancer (NSCLC). *Annals of Oncology, 23*, ixe22, <https://doi.org/https://doi.org/10.1016/S0923-7534(20)34340-4>.

S118. Vansteenkiste, J., Barlesi, F., Waller, C. F., Bennouna, J., Gridelli, C., Goekkurt, E., et al. (2015). Cilengitide combined with cetuximab and platinum-based chemotherapy as first-line treatment in advanced non-small-cell lung cancer (NSCLC) patients: results of an open-label, randomized, controlled phase II study (CERTO). *Ann Oncol, 26*(8), 1734-1740, <https://doi.org/10.1093/annonc/mdv219>.

S119. Chibaudel, B., Lacave, R., Lefevre, M., Soussan, P., Antoine, M., Périé, S., et al. (2015). Induction therapy with cetuximab plus docetaxel, cisplatin, and 5-fluorouracil (ETPF) in patients with resectable nonmetastatic stage III or IV squamous cell carcinoma of the oropharynx. A GERCOR phase II ECHO-07 study. *Cancer Med, 4*(5), 721-731, <https://doi.org/10.1002/cam4.408>.

S120. Guigay, J., Fayette, J., Dillies, A. F., Sire, C., Kerger, J. N., Tennevet, I., et al. (2015). Cetuximab, docetaxel, and cisplatin as first-line treatment in patients with recurrent or metastatic head and neck squamous cell carcinoma: a multicenter, phase II GORTEC study. *Ann Oncol, 26*(9), 1941-1947, <https://doi.org/10.1093/annonc/mdv268>.

S121. Heigener, D. F., Pereira, J. R., Felip, E., Mazal, J., Manzyuk, L., Tan, E. H., et al. (2015). Weekly and every 2 weeks cetuximab maintenance therapy after platinum-based chemotherapy plus cetuximab as first-line treatment for non-small cell lung cancer: randomized non-comparative phase IIIb NEXT trial. *Target Oncol, 10*(2), 255-265, <https://doi.org/10.1007/s11523-014-0336-7>.

S122. Trieu, V., Pinto, H., Riess, J. W., Lira, R., Luciano, R., Coty, J., et al. (2018). Weekly docetaxel, cisplatin, and cetuximab in palliative treatment of patients with squamous cell carcinoma of the head and neck. *Oncologist, 23*(7), 764-e786, <https://doi.org/10.1634/theoncologist.2017-0618>.

S123. Guigay, J., Aupérin, A., Fayette, J., Saada-Bouzid, E., Lafond, C., Taberna, M., et al. (2021). Cetuximab, docetaxel, and cisplatin versus platinum, fluorouracil, and cetuximab as first-line treatment in patients with recurrent or metastatic head and neck squamous-cell carcinoma (GORTEC 2014-01 TPExtreme): a multicentre, open-label, randomised, phase 2 trial. *Lancet Oncol, 22*(4), 463-475, <https://doi.org/10.1016/s1470-2045(20)30755-5>.

S124. Wirth, L. J., Dakhil, S. R., Kornek, G., Axelrod, R., Adkins, D., Pant, S., et al. (2013). PARTNER: A randomized phase II study of docetaxel/cisplatin (doc/cis) chemotherapy with or without panitumumab (pmab) as first-line treatment (tx) for recurrent or metastatic squamous cell carcinoma of the head and neck (R/M SCCHN). *Journal of Clinical Oncology, 31*(15_suppl), 6029-6029, <https://doi.org/10.1200/jco.2013.31.15_suppl.6029>.

S125. Laurie, S. A., Gauthier, I., Arnold, A., Shepherd, F. A., Ellis, P. M., Chen, E., et al. (2008). Phase I and pharmacokinetic study of daily oral AZD2171, an inhibitor of vascular endothelial growth factor tyrosine kinases, in combination with carboplatin and paclitaxel in patients with advanced non-small-cell lung cancer: the National Cancer Institute of Canada clinical trials group. *J Clin Oncol, 26*(11), 1871-1878, <https://doi.org/10.1200/jco.2007.14.4741>.

S126. Socinski, M. A., Langer, C. J., Huang, J. E., Kolb, M. M., Compton, P., Wang, L., et al. (2009). Safety of Bevacizumab in Patients With Non–Small-Cell Lung Cancer and Brain Metastases. *Journal of Clinical Oncology, 27*(31), 5255-5261, <https://doi.org/10.1200/jco.2009.22.0616>.

S127. Crinò, L., Dansin, E., Garrido, P., Griesinger, F., Laskin, J., Pavlakis, N., et al. (2010). Safety and efficacy of first-line bevacizumab-based therapy in advanced non-squamous non-small-cell lung cancer (SAiL, MO19390): a phase 4 study. *Lancet Oncol, 11*(8), 733-740, <https://doi.org/10.1016/s1470-2045(10)70151-0>.

S128. Tsai, C.-M., Au, J. S.-k., Chang, G.-C., Cheng, A. C.-k., Zhou, C., & Wu, Y.-l. (2011). Safety and Efficacy of First-Line Bevacizumab with Chemotherapy in Asian Patients with Advanced Nonsquamous NSCLC: Results from the Phase IV MO19390 (SAiL) Study. *Journal of Thoracic Oncology, 6*(6), 1092-1097, <https://doi.org/10.1097/JTO.0b013e318216687d>.

S129. Laskin, J., Crinò, L., Felip, E., Franke, F., Gorbunova, V., Groen, H., et al. (2012). Safety and Efficacy of First-Line Bevacizumab Plus Chemotherapy in Elderly Patients with Advanced or Recurrent Nonsquamous Non-small Cell Lung Cancer: Safety of Avastin in Lung trial (MO19390). *Journal of Thoracic Oncology, 7*(1), 203-211, <https://doi.org/https://doi.org/10.1097/JTO.0b013e3182370e02>.

S130. Shah, M. A., Jhawer, M., Ilson, D. H., Lefkowitz, R. A., Robinson, E., Capanu, M., et al. (2011). Phase II study of modified docetaxel, cisplatin, and fluorouracil with bevacizumab in patients with metastatic gastroesophageal adenocarcinoma. *J Clin Oncol, 29*(7), 868-874, <https://doi.org/10.1200/jco.2010.32.0770>.

S131. Bertino, E., Villalona-Calero, M. A., Ross, P., Grever, M., & Otterson, G. A. (2011). Preoperative bevacizumab in combination with paclitaxel and carboplatin in surgically resectable non-small cell lung cancer. *Ann Thorac Surg, 91*(2), 640, <https://doi.org/10.1016/j.athoracsur.2010.07.047>.

S132. Schefter, T. E., Winter, K., Kwon, J. S., Stuhr, K., Balaraj, K., Yaremko, B. P., et al. (2012). A phase II study of bevacizumab in combination with definitive radiotherapy and cisplatin chemotherapy in untreated patients with locally advanced cervical carcinoma: preliminary results of RTOG 0417. *Int J Radiat Oncol Biol Phys, 83*(4), 1179-1184, <https://doi.org/10.1016/j.ijrobp.2011.10.060>.

S133. Colombo, N., Zaccarelli, E., Baldoni, A., Frezzini, S., Scambia, G., Palluzzi, E., et al. (2019). Multicenter, randomised, open-label, non-comparative phase 2 trial on the efficacy and safety of the combination of bevacizumab and trabectedin with or without carboplatin in women with partially platinum-sensitive recurrent ovarian cancer. *Br J Cancer, 121*(9), 744-750, <https://doi.org/10.1038/s41416-019-0584-5>.

S134. Moore, K. N., Miller, A., Bell-McGuinn, K. M., Schilder, R. J., Walker, J. L., O'Cearbhaill, R. E., et al. (2020). A phase I study of intravenous or intraperitoneal platinum based chemotherapy in combination with veliparib and bevacizumab in newly diagnosed ovarian, primary peritoneal and fallopian tube cancer. *Gynecol Oncol, 156*(1), 13-22, <https://doi.org/10.1016/j.ygyno.2019.10.012>.

S135. Thibault, C., Fléchon, A., Albiges, L., Joly, C., Barthelemy, P., Gross-Goupil, M., et al. (2023). Gemcitabine plus platinum-based chemotherapy in combination with bevacizumab for kidney metastatic collecting duct and medullary carcinomas: results of a prospective phase II trial (BEVABEL-GETUG/AFU24). *Eur J Cancer, 186*, 83-90, <https://doi.org/10.1016/j.ejca.2023.03.018>.

S136. Doebele, R. C., Spigel, D., Tehfe, M., Thomas, S., Reck, M., Verma, S., et al. (2015). Phase 2, randomized, open-label study of ramucirumab in combination with first-line pemetrexed and platinum chemotherapy in patients with nonsquamous, advanced/metastatic non-small cell lung cancer. *Cancer, 121*(6), 883-892, <https://doi.org/10.1002/cncr.29132>.

S137. Thomas, S., Doebele, R. C., Spigel, D., Tehfe, M., Reck, M., Verma, S., et al. (2017). A phase 2 randomized open-label study of ramucirumab (RAM) plus first-line platinum-based chemotherapy in patients (pts) with recurrent or advanced non-small cell lung cancer (NSCLC): final results from squamous pts. *Ann Oncol, 28*, ii42-ii43, <https://doi.org/10.1093/annonc/mdx091.039>.

S138. Kadowaki, S., Shitara, K., Sakai, D., Nishina, T., Yoshikawa, R., Piao, Y., et al. (2017). Abstract CT096: Phase 1b study of ramucirumab (RAM) in combination with fluoropyrimidine and platinum-based agents in Japanese patients (pts) with metastatic gastric/gastroesophageal junction adenocarcinoma (mGC). *Cancer Research, 77*(13_Supplement), CT096-CT096, <https://doi.org/10.1158/1538-7445.Am2017-ct096>.

S139. Wakelee, H., Yu, W., Rittweger, K., & Paton, V. E. (2012). 1364TiP - A Randomized, Phase II, Multicenter, Double-Blind, Placebo-Controlled Study of Onartuzumab (METMAB) with Either Bevacizumab + Platinum + Paclitaxel or Pemetrexed + Platinum as First-Line Treatment for Patients (PTS) with Stage IIIB or IV Non-Squamous Non-Small Cell Lung Cancer (NSCLC). *Annals of Oncology, 23*, ix445, <https://doi.org/https://doi.org/10.1016/S0923-7534(20)33827-8>.

S140. Hirsch, F. R., Govindan, R., Zvirbule, Z., Braiteh, F., Rittmeyer, A., Belda-Iniesta, C., et al. (2017). Efficacy and Safety Results From a Phase II, Placebo-Controlled Study of Onartuzumab Plus First-Line Platinum-Doublet Chemotherapy for Advanced Squamous Cell Non-Small-Cell Lung Cancer. *Clin Lung Cancer, 18*(1), 43-49, <https://doi.org/10.1016/j.cllc.2016.05.011>.

S141. Kaye, S. B., Poole, C. J., Dańska-Bidzińska, A., Gianni, L., Del Conte, G., Gorbunova, V., et al. (2013). A randomized phase II study evaluating the combination of carboplatin-based chemotherapy with pertuzumab versus carboplatin-based therapy alone in patients with relapsed, platinum-sensitive ovarian cancer. *Ann Oncol, 24*(1), 145-152, <https://doi.org/10.1093/annonc/mds282>.

S142. Kurokawa, Y., Sugimoto, N., Miwa, H., Tsuda, M., Nishina, S., Okuda, H., et al. (2014). Phase II study of trastuzumab in combination with S-1 plus cisplatin in HER2-positive gastric cancer (HERBIS-1). *British Journal of Cancer, 110*(5), 1163-1168, <https://doi.org/10.1038/bjc.2014.18>.

S143. Mondaca, S., Margolis, M., Sanchez-Vega, F., Jonsson, P., Riches, J. C., Ku, G. Y., et al. (2019). Phase II study of trastuzumab with modified docetaxel, cisplatin, and 5 fluorouracil in metastatic HER2-positive gastric cancer. *Gastric Cancer, 22*(2), 355-362, <https://doi.org/10.1007/s10120-018-0861-7>.

S144. von Minckwitz, G., Procter, M., de Azambuja, E., Zardavas, D., Benyunes, M., Viale, G., et al. (2017). Adjuvant Pertuzumab and Trastuzumab in Early HER2-Positive Breast Cancer. *New England Journal of Medicine, 377*(2), 122-131, <https://doi.org/10.1056/NEJMoa1703643>.

S145. Piccart, M., Procter, M., Fumagalli, D., Azambuja, E. d., Clark, E., Ewer, M. S., et al. (2021). Adjuvant pertuzumab and trastuzumab in early HER2-positive breast cancer in the APHINITY trial: 6 years' follow-up. *J Clin Oncol, 39*(13), 1448-1457, <https://doi.org/10.1200/jco.20.01204>.

S146. Azambuja, E. d., Eiger, D., Procter, M. J., Ponde, N. F., Guillaume, S., Parlier, D., et al. (2021). Cardiac safety of dual anti-HER2 blockade with pertuzumab plus trastuzumab (P+T) in the APHINITY trial. *Journal of Clinical Oncology, 39*(15_suppl), 510-510, <https://doi.org/10.1200/JCO.2021.39.15_suppl.510>.

S147. Wagner, A. D., Grabsch, H. I., Mauer, M., Romario, U. F., Kang, Y.-K., Bouche, O., et al. (2023). Integration of trastuzumab (T), with or without pertuzumab (P), into perioperative chemotherapy (CT) of HER-2 positive gastric (GC) and esophagogastric junction cancer (EGJC): first results of the EORTC 1203 INNOVATION study, in collaboration with the Korean Cancer Study Group, and the Dutch Upper GI Cancer group. *J Clin Oncol, 41*(16_suppl), 4057-4057, <https://doi.org/10.1200/JCO.2023.41.16_suppl.4057>.

S148. Wagner, A. D., Grabsch, H. I., Mauer, M., Marreaud, S., Caballero, C., Thuss-Patience, P., et al. (2019). EORTC-1203-GITCG - the "INNOVATION"-trial: Effect of chemotherapy alone versus chemotherapy plus trastuzumab, versus chemotherapy plus trastuzumab plus pertuzumab, in the perioperative treatment of HER2 positive, gastric and gastroesophageal junction adenocarcinoma on pathologic response rate: a randomized phase II-intergroup trial of the EORTC-Gastrointestinal Tract Cancer Group, Korean Cancer Study Group and Dutch Upper GI-Cancer group. *BMC Cancer, 19*(1), 494, <https://doi.org/10.1186/s12885-019-5675-4>.

S149. Belani, C. P., Dahlberg, S. E., Rudin, C. M., Fleisher, M., Chen, H. X., Takebe, N., et al. (2013). Three-arm randomized phase II study of cisplatin and etoposide (CE) versus CE with either vismodegib (V) or cixutumumab (Cx) for patients with extensive stage-small cell lung cancer (ES-SCLC) (ECOG 1508). *Journal of Clinical Oncology, 31*(15_suppl), 7508-7508, <https://doi.org/10.1200/jco.2013.31.15_suppl.7508>.

S150. Gaballa, S., Palmisiano, N. D., Asija, A., Chapman, A. E., Cloud, J. K., Filicko-O'Hara, J. E., et al. (2014). Results of a phase I study of bendamustine in combination with ofatumumab, carboplatin, and etoposide (BOCE) for refractory or relapsed aggressive B-cell non-Hodgkin lymphomas (NHL). *Journal of Clinical Oncology, 32*(15_suppl), 8556-8556, <https://doi.org/10.1200/jco.2014.32.15_suppl.8556>.

S151. Glisson, B., Besse, B., Dols, M. C., Dubey, S., Schupp, M., Jain, R., et al. (2017). A Randomized, Placebo-Controlled, Phase 1b/2 Study of Rilotumumab or Ganitumab in Combination With Platinum-Based Chemotherapy as First-Line Treatment for Extensive-Stage Small-Cell Lung Cancer. *Clinical Lung Cancer, 18*(6), 615-625.e618, <https://doi.org/https://doi.org/10.1016/j.cllc.2017.05.007>.

S152. Dillon, M. T., Grove, L., Newbold, K. L., Shaw, H., Brown, N. F., Mendell, J., et al. (2019). Patritumab with cetuximab plus platinum-containing therapy in recurrent or metastatic squamous cell carcinoma of the head and neck: An open-label, phase Ib study. *Clin Cancer Res, 25*(2), 487-495, <https://doi.org/10.1158/1078-0432.Ccr-18-1539>.

S153. Forster, M. D., Dillon, M. T., Kocsis, J., Remenár, É., Pajkos, G., Rolland, F., et al. (2019). Patritumab or placebo, with cetuximab plus platinum therapy in recurrent or metastatic squamous cell carcinoma of the head and neck: A randomised phase II study. *Eur J Cancer, 123*, 36-47, <https://doi.org/10.1016/j.ejca.2019.08.017>.

S154. Herzog, T. J., Pignata, S., Ghamande, S. A., Rubio, M. J., Fujiwara, K., Vulsteke, C., et al. (2023). Randomized phase II trial of farletuzumab plus chemotherapy versus placebo plus chemotherapy in low CA-125 platinum-sensitive ovarian cancer. *Gynecol Oncol, 170*, 300-308, <https://doi.org/10.1016/j.ygyno.2023.01.003>.

S155. Herzog, T., Pignata, S., Ghamande, S., Rubio, M.-J., Fujiwara, K., Vulsteke, C., et al. (2021). A randomized, double-blind, placebo-controlled, phase II study to assess the efficacy/safety of farletuzumab in combination with carboplatin plus paclitaxel or carboplatin plus pegylated liposomal doxorubicin (PLD) in women with low CA-125 platinum-sensitive ovarian cancer. *Gynecologic Oncology, 162*, S38-S39, <https://doi.org/https://doi.org/10.1016/S0090-8258(21)00717-4>.

S156. Peters, S., Danson, S., Hasan, B., Dafni, U., Reinmuth, N., Majem, M., et al. (2020). A Randomized Open-Label Phase III Trial Evaluating the Addition of Denosumab to Standard First-Line Treatment in Advanced NSCLC: The European Thoracic Oncology Platform (ETOP) and European Organisation for Research and Treatment of Cancer (EORTC) SPLENDOUR Trial. *Journal of Thoracic Oncology, 15*(10), 1647-1656, <https://doi.org/https://doi.org/10.1016/j.jtho.2020.06.011>.

S157. Peters, S., Danson, S. J., Hasan, B., Reinmuth, N., Majem, M., Tournoy, K. G., et al. (2018). A randomised phase III trial evaluating the addition of denosumab to standard first-line treatment in advanced NSCLC: The ETOP and EORTC SPLENDOUR trial. *Annals of Oncology, 29*, viii498, <https://doi.org/10.1093/annonc/mdy292.008>.

S158. Moore, K. N., O'Malley, D. M., Vergote, I., Martin, L. P., Gonzalez-Martin, A., Malek, K., et al. (2018). Safety and activity findings from a phase 1b escalation study of mirvetuximab soravtansine, a folate receptor alpha (FRα)-targeting antibody-drug conjugate (ADC), in combination with carboplatin in patients with platinum-sensitive ovarian cancer. *Gynecol Oncol, 151*(1), 46-52, <https://doi.org/10.1016/j.ygyno.2018.07.017>.

S159. Fang, W., Yang, Y., Ma, Y., Hong, S., Lin, L., He, X., et al. (2018). Camrelizumab (SHR-1210) alone or in combination with gemcitabine plus cisplatin for nasopharyngeal carcinoma: results from two single-arm, phase 1 trials. *Lancet Oncol, 19*(10), 1338-1350, <https://doi.org/10.1016/s1470-2045(18)30495-9>.

S160. Xu, N., Ying, K., Wang, Z., Liu, Y., Jiang, H., Zhou, H., et al. (2019). Phase Ib study of sintilimab in combination with chemotherapy for 1L advanced or metastatic non-small cell lung cancer (NSCLC). *J Clin Oncol, 37*(15_suppl), e20546-e20546, <https://doi.org/10.1200/JCO.2019.37.15_suppl.e20546>.

S161. Zhou, C., Wu, L., Fan, Y., Wang, Z., Liu, L., Chen, G., et al. (2021). Sintilimab plus platinum and gemcitabine as first-line treatment for advanced or metastatic squamous NSCLC: Results from a randomized, double-blind, phase 3 trial (ORIENT-12). *J Thorac Oncol, 16*(9), 1501-1511, <https://doi.org/10.1016/j.jtho.2021.04.011>.

S162. Rodríguez-Abreu, D., Powell, S. F., Hochmair, M. J., Gadgeel, S., Esteban, E., Felip, E., et al. (2021). Pemetrexed plus platinum with or without pembrolizumab in patients with previously untreated metastatic nonsquamous NSCLC: protocol-specified final analysis from KEYNOTE-189. *Ann Oncol, 32*(7), 881-895, <https://doi.org/10.1016/j.annonc.2021.04.008>.

S163. Garassino, M. C., Gadgeel, S., Speranza, G., Felip, E., Esteban, E., Dómine, M., et al. (2023). Pembrolizumab plus pemetrexed and platinum in nonsquamous non-small-cell lung cancer: 5-Year outcomes from the phase 3 KEYNOTE-189 study. *J Clin Oncol, 41*(11), 1992-1998, <https://doi.org/10.1200/jco.22.01989>.

S164. Garassino, M. C., Gadgeel, S., Esteban, E., Felip, E., Speranza, G., Domine, M., et al. (2020). Patient-reported outcomes following pembrolizumab or placebo plus pemetrexed and platinum in patients with previously untreated, metastatic, non-squamous non-small-cell lung cancer (KEYNOTE-189): a multicentre, double-blind, randomised, placebo-controlled, phase 3 trial. *Lancet Oncol, 21*(3), 387-397, <https://doi.org/10.1016/s1470-2045(19)30801-0>.

S165. Gadgeel, S., Rodríguez-Abreu, D., Speranza, G., Esteban, E., Felip, E., Dómine, M., et al. (2020). Updated Analysis From KEYNOTE-189: Pembrolizumab or Placebo Plus Pemetrexed and Platinum for Previously Untreated Metastatic Nonsquamous Non-Small-Cell Lung Cancer. *J Clin Oncol, 38*(14), 1505-1517, <https://doi.org/10.1200/jco.19.03136>.

S166. Menis, J., Bironzo, P., Radj, G., Greillier, L., Monnet, I., Livi, L., et al. (2020). 9P Circulating tumour cells (CTCs) count and PD-L1 expression in untreated extensive small cell lung cancer patients treated in the REACTION trial, a phase II study of etoposide and cis/carboplatin with or without pembrolizumab (NCT02580994). *Ann Oncol, 31*, S1420, <https://doi.org/10.1016/j.annonc.2020.10.494>.

S167. Janjigian, Y. Y., Maron, S. B., Chatila, W. K., Millang, B., Chavan, S. S., Alterman, C., et al. (2020). First-line pembrolizumab and trastuzumab in HER2-positive oesophageal, gastric, or gastro-oesophageal junction cancer: an open-label, single-arm, phase 2 trial. *Lancet Oncol, 21*(6), 821-831, <https://doi.org/10.1016/s1470-2045(20)30169-8>.

S168. Rudin, C. M., Awad, M. M., Navarro, A., Gottfried, M., Peters, S., Csőszi, T., et al. (2020). Pembrolizumab or placebo plus etoposide and platinum as first-Line therapy for extensive-stage small-cell lung cancer: randomized, double-blind, phase III KEYNOTE-604 study. *J Clin Oncol, 38*(21), 2369-2379, <https://doi.org/10.1200/jco.20.00793>.

S169. Horinouchi, H., Nogami, N., Saka, H., Nishio, M., Tokito, T., Takahashi, T., et al. (2021). Pembrolizumab plus pemetrexed-platinum for metastatic nonsquamous non-small-cell lung cancer: KEYNOTE-189 Japan Study. *Cancer Sci, 112*(8), 3255-3265, <https://doi.org/10.1111/cas.14980>.

S170. Walsh, C. S., Kamrava, M., Rogatko, A., Kim, S., Li, A., Cass, I., et al. (2021). Phase II trial of cisplatin, gemcitabine and pembrolizumab for platinum-resistant ovarian cancer. *PLoS One, 16*(6), e0252665, <https://doi.org/10.1371/journal.pone.0252665>.

S171. Powles, T., Csőszi, T., Özgüroğlu, M., Matsubara, N., Géczi, L., Cheng, S. Y., et al. (2021). Pembrolizumab alone or combined with chemotherapy versus chemotherapy as first-line therapy for advanced urothelial carcinoma (KEYNOTE-361): a randomised, open-label, phase 3 trial. *Lancet Oncol, 22*(7), 931-945, <https://doi.org/10.1016/s1470-2045(21)00152-2>.

S172. Liao, J. B., Gwin, W. R., Urban, R. R., Hitchcock-Bernhardt, K. M., Coveler, A. L., Higgins, D. M., et al. (2021). Pembrolizumab with low-dose carboplatin for recurrent platinum-resistant ovarian, fallopian tube, and primary peritoneal cancer: survival and immune correlates. *J Immunother Cancer, 9*(9), e003122, <https://doi.org/10.1136/jitc-2021-003122>.

S173. Barber, E. L., Chen, S., Pineda, M. J., Robertson, S. E., Hill, E. K., Teoh, D., et al. (2022). Clinical and biological activity of chemoimmunotherapy in advanced endometrial adenocarcinoma: a phase II trial of the Big Ten Cancer Research Consortium. *Cancer Res Commun, 2*(10), 1293-1303, <https://doi.org/10.1158/2767-9764.Crc-22-0147>.

S174. Chin, A. I., Ly, A., Rodriguez, S., Sachdeva, A., Zomorodian, N., Zhang, H., et al. (2023). Updated results of a phase Ib single-center study of pembrolizumab in combination with chemotherapy in patients with locally advanced or metastatic small cell/neuroendocrine cancers of the prostate and urothelium. *J Clin Oncol, 41*(6_suppl), 165-165, <https://doi.org/10.1200/JCO.2023.41.6_suppl.165>.

S175. Bar, J., Esteban, E., Rodríguez-Abreu, D., Aix, S. P., Szalai, Z., Felip, E., et al. (2023). Abstract CT216: Response to first-line (1L) pembrolizumab (pembro) + chemotherapy (chemo) in non-small cell lung cancer (NSCLC) by blood tumor mutational burden (bTMB): the phase 2 KEYNOTE-782 trial. *Cancer Research, 83*(8_Supplement), CT216-CT216, <https://doi.org/10.1158/1538-7445.Am2023-ct216>.

S176. Kelley, R. K., Ueno, M., Yoo, C., Finn, R. S., Furuse, J., Ren, Z., et al. (2023). Pembrolizumab in combination with gemcitabine and cisplatin compared with gemcitabine and cisplatin alone for patients with advanced biliary tract cancer (KEYNOTE-966): a randomised, double-blind, placebo-controlled, phase 3 trial. *Lancet, 401*(10391), 1853-1865, <https://doi.org/10.1016/s0140-6736(23)00727-4>.

S177. Rha, S. Y., Oh, D. Y., Yañez, P., Bai, Y., Ryu, M. H., Lee, J., et al. (2023). Pembrolizumab plus chemotherapy versus placebo plus chemotherapy for HER2-negative advanced gastric cancer (KEYNOTE-859): a multicentre, randomised, double-blind, phase 3 trial. *Lancet Oncol, 24*(11), 1181-1195, <https://doi.org/10.1016/s1470-2045(23)00515-6>.

S178. Zhang, Y., Zeng, L., Zhang, X., Zhou, Y., Zhang, B., Jiang, W., et al. (2021). P15.02 Toripalimab and platinum-doublet chemotherapy as neoadjuvant therapy for potentially resectable non-small cell lung cancer. *J Thorac Oncol, 16*(10), S1014-S1015, <https://doi.org/10.1016/j.jtho.2021.08.339>.

S179. Clarke, J. M., Patel, J. D., Robert, F., Kio, E. A., Thara, E., Ross Camidge, D., et al. (2021). Veliparib and nivolumab in combination with platinum doublet chemotherapy in patients with metastatic or advanced non-small cell lung cancer: a phase 1 dose escalation study. *Lung Cancer, 161*, 180-188, <https://doi.org/10.1016/j.lungcan.2021.09.004>.

S180. Hui, R., Munteanu, M., Zhao, Y., Luo, Y., Samkari, A., & Garassino, M. C. (2018). ECHO-306/KEYNOTE-715: A phase 3 study of first-line epacadostat plus pembrolizumab with or without platinum-based chemotherapy vs pembrolizumab plus platinum-based chemotherapy plus placebo for metastatic non–small cell lung cancer (mNSCLC). *Journal of Clinical Oncology, 36*(15_suppl), TPS9104-TPS9104, <https://doi.org/10.1200/JCO.2018.36.15_suppl.TPS9104>.

S181. Powderly, J. D., Klempner, S. J., Naing, A., Bendell, J., Garrido-Laguna, I., Catenacci, D. V. T., et al. (2022). Epacadostat Plus Pembrolizumab and Chemotherapy for Advanced Solid Tumors: Results from the Phase I/II ECHO-207/KEYNOTE-723 Study. *Oncologist, 27*(11), 905-e848, <https://doi.org/10.1093/oncolo/oyac174>.

S182. Ready, N., Hellmann, M. D., Awad, M. M., Otterson, G. A., Gutierrez, M., Gainor, J. F., et al. (2019). First-Line Nivolumab Plus Ipilimumab in Advanced Non-Small-Cell Lung Cancer (CheckMate 568): Outcomes by Programmed Death Ligand 1 and Tumor Mutational Burden as Biomarkers. *J Clin Oncol, 37*(12), 992-1000, <https://doi.org/10.1200/jco.18.01042>.

S183. Banerjee, S., Vergotte, I., Colombo, N., Barve, M., Grisham, R., Mehr, K. T., et al. (2019). 1060TiP - Randomized, phase Ib/II study of M6620 + avelumab + carboplatin vs standard care (sc) in patients (pts) with platinum-sensitive poly (ADP-ribose) polymerase inhibitor-(PARPi)-resistant ovarian cancer. *Annals of Oncology, 30*, v431-v432, <https://doi.org/https://doi.org/10.1093/annonc/mdz250.068>.

S184. Isla, D., Arriola, E., Garcia Campelo, M. R., Diz Tain, P., Marti Blanco, C., Lopez-Brea Piqueras, M. M., et al. (2022). 1532P Phase IIIb study of durvalumab plus platinum-etoposide in first-line treatment of extensive-stage small cell lung cancer (CANTABRICO): Preliminary efficacy results. *Annals of Oncology, 33*, S1247-S1248, <https://doi.org/10.1016/j.annonc.2022.07.1627>.

S185. Oh, D.-Y., He, A. R., Qin, S., Chen, L.-T., Okusaka, T., Vogel, A., et al. (2022). Durvalumab plus Gemcitabine and Cisplatin in Advanced Biliary Tract Cancer. *NEJM Evidence, 1*(8), EVIDoa2200015, <https://doi.org/doi:10.1056/EVIDoa2200015>.

S186. Lee, D. H., Kim, H. R., Keam, B., Kato, K., Kuboki, Y., Gao, H., et al. (2023). Safety and tolerability of first-line durvalumab with tremelimumab and chemotherapy in esophageal squamous cell carcinoma. *Cancer Med, 12*(15), 16066-16075, <https://doi.org/10.1002/cam4.6260>.

S187. Rolfo, C., Greillier, L., Veillon, R., Badin, F., Ghiringhelli, F., Isambert, N., et al. (2021). 465 Bintrafusp alfa in combination with chemotherapy in patients with stage IV NSCLC: safety and pharmacokinetic results of the INTR@PID LUNG 024 study. *Journal for ImmunoTherapy of Cancer, 9*(Suppl 2), A494-A494, <https://doi.org/10.1136/jitc-2021-SITC2021.465>.

S188. Vugmeyster, Y., Grisic, A. M., Wilkins, J. J., Loos, A. H., Hallwachs, R., Osada, M., et al. (2022). Model-informed approach for risk management of bleeding toxicities for bintrafusp alfa, a bifunctional fusion protein targeting TGF-β and PD-L1. *Cancer Chemother Pharmacol, 90*(4), 369-379, <https://doi.org/10.1007/s00280-022-04468-6>.

S189. Karapanagiotou, E. M., Chester, J. D., Pandha, H. S., Gill, G. M., Coffey, M. C., Mettinger, K., et al. (2010). A phase I/II study of oncolytic reovirus plus carboplatin/paclitaxel in patients with advanced solid cancers with emphasis on squamous cell carcinoma of the head and neck (SCCHN). *Journal of Clinical Oncology, 28*(15_suppl), 3080-3080, <https://doi.org/10.1200/jco.2010.28.15_suppl.3080>.

S190. Karapanagiotou, E. M., Roulstone, V., Twigger, K., Ball, M., Tanay, M., Nutting, C., et al. (2012). Phase I/II trial of carboplatin and paclitaxel chemotherapy in combination with intravenous oncolytic reovirus in patients with advanced malignancies. *Clin Cancer Res, 18*(7), 2080-2089, <https://doi.org/10.1158/1078-0432.Ccr-11-2181>.

S191. Harrington, K. J., Karapanagiotou, E. M., Hardev, P., Nutting, C. M., Gore, M., Karl, M., et al. (2009). Abstract B242: A phase I/II study of oncolytic reovirus plus chemotherapy (carboplatin/paclitaxel) in patients with advanced solid tumors (with emphasis on patients with squamous cell carcinoma of the head and neck (SCCHN). *Molecular Cancer Therapeutics, 8*(12_Supplement), B242-B242, <https://doi.org/10.1158/1535-7163.Targ-09-b242>.

S192. Noonan, A. M., Farren, M. R., Geyer, S. M., Huang, Y., Tahiri, S., Ahn, D., et al. (2016). Randomized phase 2 trial of the oncolytic virus pelareorep (Reolysin) in upfront treatment of metastatic pancreatic adenocarcinoma. *Mol Ther, 24*(6), 1150-1158, <https://doi.org/10.1038/mt.2016.66>.

S193. Mahalingam, D., Fountzilas, C., Moseley, J., Noronha, N., Tran, H., Chakrabarty, R., et al. (2017). A phase II study of REOLYSIN(®) (pelareorep) in combination with carboplatin and paclitaxel for patients with advanced malignant melanoma. *Cancer Chemother Pharmacol, 79*(4), 697-703, <https://doi.org/10.1007/s00280-017-3260-6>.

S194. Anwer, K., Kelly, F. J., Chu, C., Fewell, J. G., Lewis, D., & Alvarez, R. D. (2013). Phase I trial of a formulated IL-12 plasmid in combination with carboplatin and docetaxel chemotherapy in the treatment of platinum-sensitive recurrent ovarian cancer. *Gynecol Oncol, 131*(1), 169-173, <https://doi.org/10.1016/j.ygyno.2013.07.081>.

S195. Paik, P. K., Luo, J., Ai, N., Kim, R., Ahn, L., Biswas, A., et al. (2022). Phase I trial of the TNF-α inhibitor certolizumab plus chemotherapy in stage IV lung adenocarcinomas. *Nat. Commun., 13*(1), 6095, <https://doi.org/10.1038/s41467-022-33719-6>.

S196. Cibula, D., Rob, L., Mallmann, P., Knapp, P., Klat, J., Chovanec, J., et al. (2021). Dendritic cell-based immunotherapy (DCVAC/OvCa) combined with second-line chemotherapy in platinum-sensitive ovarian cancer (SOV02): a randomized, open-label, phase 2 trial. *Gynecol Oncol, 162*(3), 652-660, <https://doi.org/10.1016/j.ygyno.2021.07.003>.

S197. Koeneman, B. J., Schreibelt, G., Gorris, M. A. J., Hins-de Bree, S., Westdorp, H., Ottevanger, P. B., et al. (2024). Dendritic cell vaccination combined with carboplatin/paclitaxel for metastatic endometrial cancer patients: results of a phase I/II trial. *Front Immunol, 15*, 1368103, <https://doi.org/10.3389/fimmu.2024.1368103>.
